# Genomic Determinants of Protein Abundance Variation in Colorectal Cancer Cells

**DOI:** 10.1016/j.celrep.2017.08.010

**Published:** 2017-08-29

**Authors:** Theodoros I. Roumeliotis, Steven P. Williams, Emanuel Gonçalves, Clara Alsinet, Martin Del Castillo Velasco-Herrera, Nanne Aben, Fatemeh Zamanzad Ghavidel, Magali Michaut, Michael Schubert, Stacey Price, James C. Wright, Lu Yu, Mi Yang, Rodrigo Dienstmann, Justin Guinney, Pedro Beltrao, Alvis Brazma, Mercedes Pardo, Oliver Stegle, David J. Adams, Lodewyk Wessels, Julio Saez-Rodriguez, Ultan McDermott, Jyoti S. Choudhary

**Affiliations:** 1 Wellcome Trust Sanger Institute, Wellcome Genome Campus, Cambridge CB10 1SA, UK; 2 European Molecular Biology Laboratory, European Bioinformatics Institute (EMBL-EBI), Wellcome Genome Campus, Cambridge CB10 1SD, UK; 3 Faculty of Medicine, Joint Research Center for Computational Biomedicine, RWTH Aachen University, Aachen 52057, Germany; 4 Division of Molecular Carcinogenesis, Computational Cancer Biology, the Netherlands Cancer Institute, Amsterdam 1066, the Netherlands; 5 Faculty of EEMCS, Delft University of Technology, Delft 2628, the Netherlands; 6 Computational Oncology, Sage Bionetworks, Fred Hutchinson Cancer Research Center, Seattle, WA 98109-1024, USA; 7 Oncology Data Science Group, Vall d’Hebron Institute of Oncology, Barcelona 08035, Spain; 8 Functional Proteomics Group, Chester Beatty Laboratories, The Institute of Cancer Research, London SW3 6JB, UK

**Keywords:** proteomics, TMT, protein complexes, networks, phosphorylation, mutations, CRISPR/cas9, colorectal cancer, cell lines, drug response

## Abstract

Assessing the impact of genomic alterations on protein networks is fundamental in identifying the mechanisms that shape cancer heterogeneity. We have used isobaric labeling to characterize the proteomic landscapes of 50 colorectal cancer cell lines and to decipher the functional consequences of somatic genomic variants. The robust quantification of over 9,000 proteins and 11,000 phosphopeptides on average enabled the de novo construction of a functional protein correlation network, which ultimately exposed the collateral effects of mutations on protein complexes. CRISPR-cas9 deletion of key chromatin modifiers confirmed that the consequences of genomic alterations can propagate through protein interactions in a transcript-independent manner. Lastly, we leveraged the quantified proteome to perform unsupervised classification of the cell lines and to build predictive models of drug response in colorectal cancer. Overall, we provide a deep integrative view of the functional network and the molecular structure underlying the heterogeneity of colorectal cancer cells.

## Highlights

• The cancer cell “co-variome” recapitulates functional protein associations
• Loss-of-function mutations can impact protein levels beyond mRNA regulation
• The consequences of genomic alterations can propagate through protein interactions
• We provide insight into the molecular organization of colorectal cancer cells

## Graphical Abstract

**Figure:**
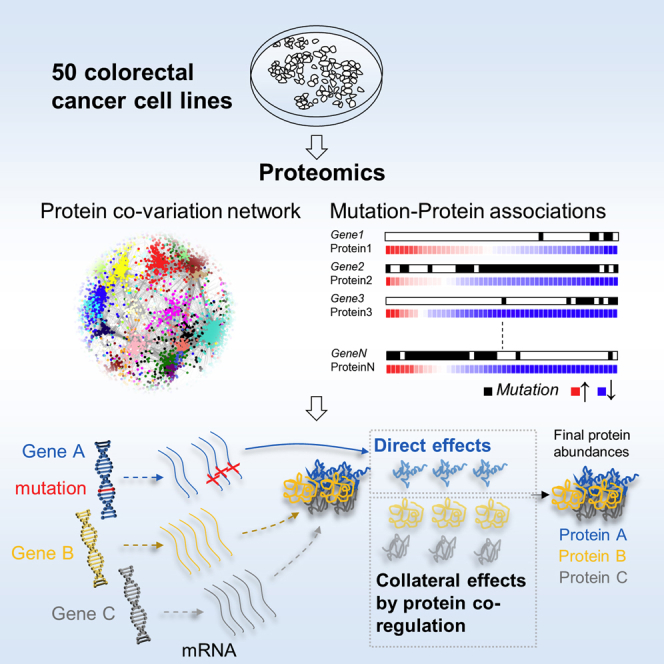

## Teaser

Roumeliotis et al. use in-depth proteomics to assess the impact of genomic alterations on protein networks in colorectal cancer cell lines. Cell-line-specific network signatures are inferred de novo by protein quantification profiles and ultimately expose the collateral and transcript-independent effects of detrimental mutations on protein complexes.

## Introduction

Tumors exhibit a high degree of molecular and cellular heterogeneity due to the impact of genomic aberrations on protein networks underlying physiological cellular activities. Modern mass-spectrometry-based proteomic technologies have the capacity to perform highly reliable analytical measurements of proteins in large numbers of subjects and analytes, providing a powerful tool for the discovery of regulatory associations between genomic features, gene expression patterns, protein networks, and phenotypic traits (Mertins et al., 2016, Zhang et al., 2014, Zhang et al., 2016). However, understanding how genomic variation affects protein networks and leads to variable proteomic landscapes and distinct cellular phenotypes remains challenging due to the enormous diversity in the biological characteristics of proteins. Studying protein co-variation holds the promise to overcome the challenges associated with the complexity of proteomic landscapes as it enables grouping of multiple proteins into functionally coherent groups and is now gaining ground in the study of protein associations (Stefely et al., 2016, Wang et al., 2017). Colorectal cancer cell lines are widely used as cancer models; however, their protein and phosphoprotein co-variation networks and the genomic factors underlying their regulation remain largely unexplored.

Here, we studied a panel of 50 colorectal cancer cell lines (colorectal adenocarcinoma [COREAD]) using isobaric labeling and tribrid mass spectrometry proteomic analysis in order to assess the impact of somatic genomic variants on protein networks. This panel has been extensively characterized by whole-exome sequencing, gene expression profiling, copy number and methylation profiling, and the frequency of molecular alterations is similar to that seen in clinical colorectal cohorts (Iorio et al., 2016). First, we leveraged the robust quantification of over 9,000 proteins to build de novo protein co-variation networks, and we show that they are highly representative of known protein complexes and interactions. Second, we rationalize the impact of genomic variation in the context of the cancer cell protein co-variation network (henceforth, “co-variome”) to uncover protein network vulnerabilities. Proteomic and RNA sequencing (RNA-seq) analysis of human induced pluripotent stem cells (iPSCs) engineered with gene knockouts of key chromatin modifiers confirmed that genomic variation can be transmitted from directly affected proteins to tightly co-regulated distant gene products through protein interactions. Overall, our results constitute an in-depth view of the molecular organization of colorectal cancer cells widely used in cancer research.

## Results

### Quantified Proteome and Phosphoproteome Coverage and Correlation with Gene Expression

To assess the variation in protein abundance and phosphorylation within a panel of 50 colorectal cancer cell lines (COREAD), we utilized isobaric peptide labeling (TMT-10plex) and MS3 quantification (Figure S1A). We obtained relative quantification between the different cell lines (scaled intensities range: 0–1,000) for an average of 9,410 proteins and 11,647 phosphopeptides (Tables S1 and S2; Figure S1B). To assess the reproducibility of our data, we computed the coefficient of variation (CV) (CV = SD/mean) of protein abundances for 11 cell lines measured as biological replicates. The median CV in our study was 10.5%, showing equivalent levels of intra-laboratory biological variation with previously published TMT data for seven colorectal cancer cell lines (McAlister et al., 2014; Figure S1C). Inter-laboratory comparison for the 7 cell lines common in both studies showed median CV = 13.9% (Figure S1C). The additional variation encompasses differences in sample preparation methods (e.g., digestion enzymes), mass spectrometry instrumentation, and raw signal processing. The same SW48 protein digest aliquoted in two parts and labeled with two different TMT labels within the same 10plex experiment displayed a median CV = 1.9% (Figure S1C), indicating that the labeling procedure and the mass spectrometry (MS) signal acquisition noise have very small contribution to the total variation. The protein abundance profiles for 11 cell lines measured as biological replicates in two separate sets are shown as a heatmap in Figure S1D, revealing the high heterogeneity of the COREAD proteomic landscapes. The variation between different cell lines was on average 3 times higher than the variation between replicates (Figure S1E), with 93% of the proteins exhibiting an inter-sample variation greater than the respective baseline variation between replicates. For proteins participating in basic cellular processes (selected Kyoto encyclopedia of genes and genomes [KEGG] pathways), the median CV between biological replicates was as low as 8% (Figure S1F). At the phosphopeptide level, the SW48 biological replicates across all multiplex sets displayed a median CV = 22% (Figure S1G), reflecting the generally higher uncertainty of the peptide level measurements compared to the protein level measurements. Taken together, our results show that protein abundance differences as low as 50% or 1.5-fold (>2 × CV%) can be reliably detected using our proteomics approach at both the proteome and phosphoproteome level.

Qualitatively, phosphorylated proteins (n = 3,565) were highly enriched for spliceosomal and cell cycle functions and covered a wide range of cancer-related pathways (Figure S2A). The phosphosites were comprised of 86% serine, 13% threonine, and <1% tyrosine phosphorylation (Figure S2B), and the most frequent motifs identified were pS-P (47% of all pS) and pT-P (63% of all pT) (Figure S2C). Approximately 70% of the quantified phosphorylation sites are cataloged in Uniprot, and 751 of these represent known kinase substrates in the PhosphoSitePlus database (Hornbeck et al., 2015). In terms of phosphorylation quantification, we observed that phosphorylation profiles were strongly correlated with the respective protein abundances (Figure S2D), and therefore, to detect net phosphorylation changes, we corrected the phosphorylation levels for total protein changes by linear regression.

Correlation analysis between mRNA (publicly available microarray data) and relative protein abundances for each gene across the cell lines indicated a pathway-dependent concordance of protein/mRNA expression with median Pearson’s r = 0.52 (Figure S2E). Highly variable mRNAs tend to correspond to highly variable proteins (Spearman’s r = 0.62), although with a wide distribution (Figure S2F). Notably, several genes, including *TP53*, displayed high variation at the protein level despite the low variation at the mRNA level, implicating significant post-transcriptional modulation of their abundance.

Our COREAD proteomics and phosphoproteomics data can be downloaded from ftp://ngs.sanger.ac.uk/production/proteogenomics/WTSI_proteomics_COREAD/ in annotated ^∗^.gct, ^∗^.gtf, and ^∗^.bb file formats compatible with the Integrative Genomics Viewer (Robinson et al., 2011), the Morpheus clustering web tool (https://software.broadinstitute.org/morpheus/), or the Ensembl (Aken et al., 2017) and University of California Santa Cruz (UCSC) (Kent et al., 2002) genome browsers. Our proteomics data can also be viewed through the Expression Atlas database (Petryszak et al., 2016).

### The Subunits of Protein Complexes Tightly Maintain Their Total Abundance Ratios Post-transcriptionally

**Figure 1:**
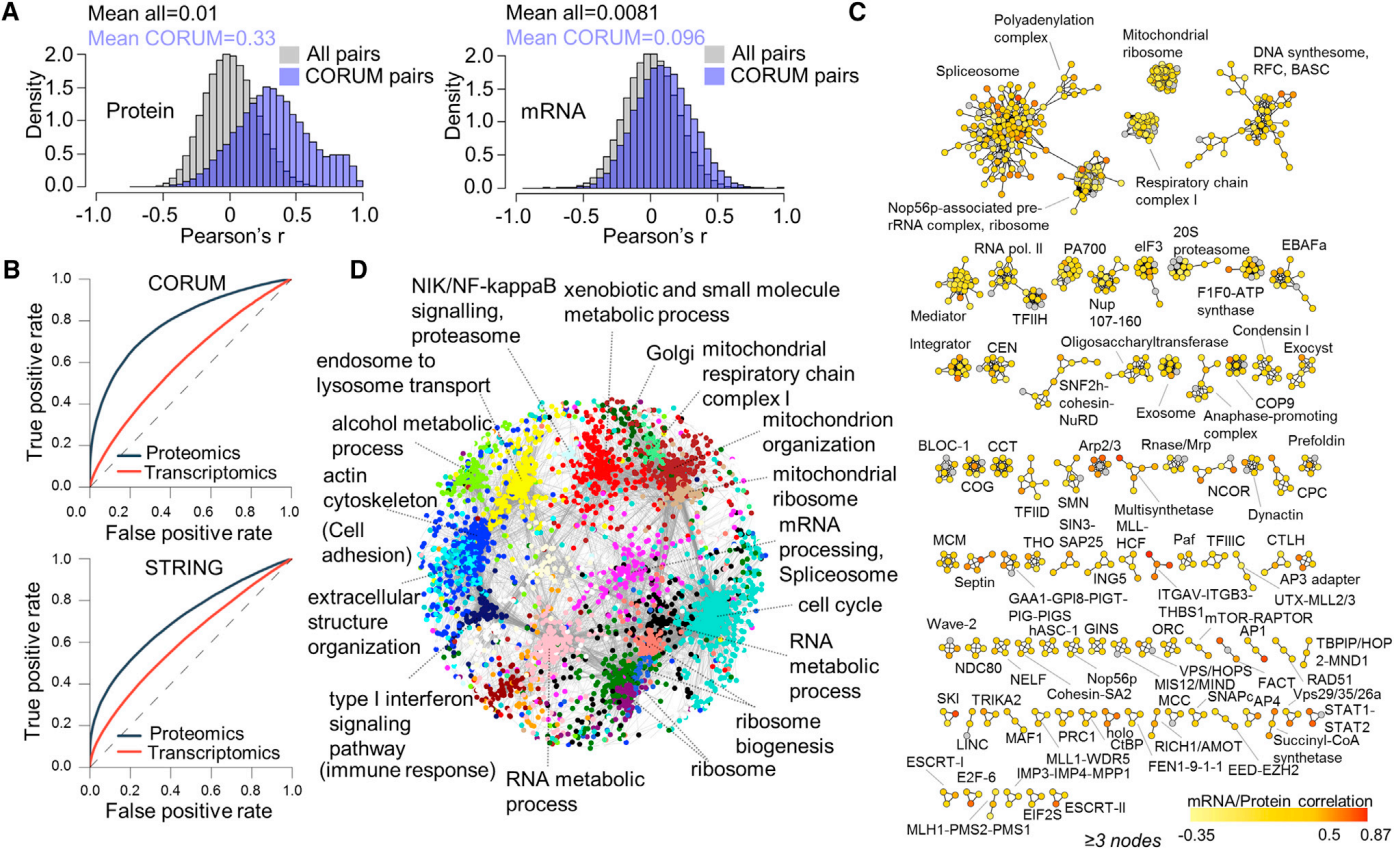
Global Distributions of Gene-to-Gene Correlations and Protein Co-variation Networks in Colorectal Cancer Cell Lines (A) Distributions of Pearson’s correlation coefficients between protein-protein pairs (left panel) and mRNA-mRNA pairs (right panel) for all pairs (gray) and for pairs with known interactions in the CORUM database (blue). (B) Receiver operating characteristic (ROC) curves illustrating the performance of proteomics- and transcriptomics-based correlations to predict CORUM and high-confident STRING interactions. (C) Protein abundance correlation networks derived from WGCNA analysis for enriched CORUM complexes. The nodes are color-coded according to mRNA-to-protein Pearson correlation. (D) The global structure of the WGCNA network using modules with more than 50 nodes. Protein modules are color coded according to the WGCNA module default name, and representative enriched terms are used for the annotation of the network. See also Figure S3.

The protein abundance measurements allowed us to study the extent to which proteins tend to be co-regulated in abundance across the colorectal cancer cell lines. We first computed the Pearson’s correlation coefficients between proteins with known physical interactions in protein complexes cataloged in the CORUM database (Ruepp et al., 2010). We found that the distribution of correlations between CORUM protein pairs was bimodal and clearly shifted to positive values (Wilcoxon test; p value < 2.2e−16) with mean 0.33 (Figure 1A, left panel), whereas all pairwise protein-to-protein correlations displayed a normal distribution with mean 0.01 (Figure 1A, left panel). Specifically, 290 partially overlapping CORUM complexes showed a greater than 0.5 median correlation between their subunits (Table S3). It has been shown that high-stoichiometry interactors are more likely to be coherently expressed across different cell types (Hein et al., 2015); therefore, our correlation data offer an assessment of the stability of known protein complexes in the context of colorectal cancer cells. Moreover, less stable or context-dependent interactions in known protein complexes may be identified by outlier profiles. Such proteins, with at least 50% lower individual correlation compared to the average complex correlation, are highlighted in Table S3. For example, the ORC1 and ORC6 proteins displayed a divergent profile from the average profile of the ORC complex, which is in line with their distinct roles in the replication initiation processes (Ohta et al., 2003, Prasanth et al., 2002).

In contrast, the distribution of Pearson’s coefficients between CORUM pairs based on mRNA co-variation profiles was only slightly shifted toward higher correlations with mean = 0.096 (Figure 1A, right panel). Interestingly, proteins with strong correlations within protein complexes showed low variation across the COREAD panel (Figure S2G) and have poor correspondence to mRNA levels (Figure S2H). Together, these suggest that the subunits of most of the known protein complexes are regulated post-transcriptionally to accurately maintain stable ratio of total abundance. Receiver operating characteristic (ROC) analyses confirmed that our proteomics data outperformed mRNA data in predicting protein complexes as well as high confident STRING interactions (Szklarczyk et al., 2015; CORUM ROC area under the curve [AUC]: 0.79 versus 0.61; STRING ROC AUC: 0.71 versus 0.61; for proteomics and gene expression, respectively; Figure 1B). The ability to also predict any type of STRING interaction suggests that protein co-variation also encompasses a broader range of functional relationships beyond structural physical interactions. Overall, our results demonstrate that correlation analysis of protein abundances across a limited set of cellular samples with variable genotypes can generate co-variation signatures for many known protein-protein interactions and protein complexes.

### The Colorectal Cancer Cell Protein Correlation Network

We conducted a systematic un-biased genome-wide analysis to characterize the colorectal cancer cell protein-protein correlation network and to identify de novo modules of interconnected proteins. To this end, we performed a weighted correlation network analysis (WGCNA) (Langfelder and Horvath, 2008) using 8,295 proteins quantified in at least 80% of the cell lines. A total of 284 protein modules ranging in size from 3 to 1,012 proteins (Q_1_ = 6; Q_3_ = 18) were inferred covering the entire input dataset. An interaction weight was assigned to each pair of correlating proteins based on their profile similarities and the properties of the network. We performed Gene Ontology annotation of the modules with the WGCNA package as well as using additional terms from CORUM, KEGG, GOBP-slim, GSEA, and Pfam databases with a Fisher’s exact test (Benjamini-Hochberg [Benj. Hoch.] false discovery rate [FDR] < 0.05). We found significantly enriched terms for 235 modules (Table S4) with an average annotation coverage of 40%. Specifically, 111 modules displayed overrepresentation of CORUM protein complexes. For 29 of the 49 not-annotated modules, we detected known STRING interactions within each module, suggesting that these also capture functional associations that do not converge to specific terms.

The correlation networks of protein complexes with more than 2 nodes are shown in Figure 1C. The global structure of the colorectal cancer network comprised of modules with at least 50 proteins is depicted in Figure 1D and is annotated by significant terms. The entire WGCNA network contains 87,420 interactions (weight > 0.02; 96% positive; mean Pearson’s r = 0.61), encompassing 7,248 and 20,969 known CORUM and STRING interactions of any confidence, respectively. Overlaying the protein abundance levels on the network generates a unique quantitative map of the cancer cell co-variome, which can help discriminate the different biological characteristics of the cell lines (Figure S3A). For instance, it can be inferred that the CL-40 cell line is mainly characterized by low abundances of cell cycle, ribosomal, and RNA metabolism proteins, which uniquely coincide with increased abundances of immune response proteins (Figure S3A). The full WGCNA network with weights greater than 0.02 is provided in Table S5.

As most of the proteins in modules representing protein complexes are poorly correlated with mRNA levels, we next sought to understand the transcriptional regulation of the modules with the highest mean mRNA-to-protein correlations (5^th^ quantile; mean Pearson’s r > 0.57; 41 modules; 1,497 proteins). These included several large components of the co-variome (e.g., “cell adhesion,” “small molecule metabolic process,” and “innate immune response”), modules showing enrichment for experimental gene sets (based on gene set enrichment analysis [GSEA]), and modules containing proteins encoded by common chromosomal regions, implicating the effects of DNA copy number variations (Figure S3B). In order to further annotate the modules with potential transcriptional regulators, we examined whether transcription factors that are members of the large transcriptionally regulated modules are co-expressed along with their target genes at the protein level. Transcription factor enrichment analysis (Kuleshov et al., 2016) indicated that the “xenobiotic and small molecule metabolic process” module was enriched for the transcription factors HNF4A and CDX2 and that STAT1/STAT2 were the potential master regulators of the “immune response” module (Figure S3B, top left panel). HNF4A (hepatocyte nuclear factor 4-alpha) is an important regulator of metabolism, cell junctions, and the differentiation of intestinal epithelial cells (Garrison et al., 2006) and has been previously associated with colorectal cancer proteomic subtypes in human tumors analyzed by the CPTAC consortium (Zhang et al., 2014). Here, we were able to further characterize the consequences of HNF4A variation through its proteome regulatory network.

To globally understand the interdependencies of protein complexes in the colorectal cancer cells, we plotted the module-to-module relationships as a correlation heatmap using only modules enriched for protein complexes. The representative profile of each module (eigengene or first principal component; Langfelder and Horvath, 2007) was used as a metric (Figure S3C). This analysis captures known functional associations between protein complexes (e.g., MCM-ORC, spliceosome-polyadenylation, and THO-nuclear pore; Lei and Tye, 2001, Millevoi et al., 2006, Wickramasinghe and Laskey, 2015) and reveals the higher order organization of the proteome. The major clusters of the correlation map can be categorized into three main themes: (1) gene expression/splicing/translation/cell cycle; (2) protein processing and trafficking; and (3) mitochondrial functions. This demonstrates that such similarity profiling of abundance signatures has the potential to uncover novel instances of cross-talk between protein complexes and also to discriminate sub-complexes within larger protein assemblies.

In addition to protein abundance co-variation, the scale of global phosphorylation survey accomplished here offers the opportunity for the de novo prediction of kinase-substrate associations inferred by co-varying phosphorylation patterns that involve kinases (Ochoa et al., 2016, Petsalaki et al., 2015). Correlation analysis among 436 phosphopeptides attributed to 137 protein kinases and 29 protein phosphatases yielded 186 positive and 40 negative associations at Benj. Hoch. FDR < 0.1 (Figure S4A), representing the co-phosphorylation signature of kinases and phosphatases in the COREAD panel. Using this high-confidence network as the baseline, we next focused on co-phosphorylation profiling of kinases and phosphatases involved in KEGG signaling pathways (Figure S4B), where known kinase relationships can be used to assess the validity of the predictions. We found co-regulated phosphorylation between RAF1, MAPK1, MAPK3, and RPS6KA3, which were more distantly correlated with the co-phosphorylated BRAF and ARAF protein kinases, all members of the mitogen-activated protein kinase (MAPK) pathway core axis (Figure S4B). MAP2K1 (or MEK1) was found phosphorylated at T388 (unknown kinase substrate), which was not correlating with the above profile. The S222 phosphorylation site of MAP2K2 (or MEK2), regulated by RAF kinase, was not detected possibly due to limitations related to the lengthy (22 amino acids) theoretical overlapping peptide. Strongly maintained co-phosphorylation between CDK1, CDK2, and CDK7 of the cell cycle pathway was another true positive example (Figure S4B). The correlation plots of MAPK1 and MAPK3 phosphorylation and total protein are depicted in Figure S4C, top panel. The co-phosphorylation of BRAF and ARAF is depicted in Figure S4C, bottom left panel. A negative correlation example (between CDK1 kinase and PPP2R5D phosphatase), reflecting the known role of PPP2R5D as an upstream negative regulator of CDK1 (Forester et al., 2007), is shown in Figure S4C, bottom right panel.

Taken together, our correlation analyses reveal the higher-order organization of cellular functions. This well-organized structure is shaped by the compartmental interactions between protein complexes, and it is clearly divided into transcriptionally and post-transcriptionally regulated sectors. The analysis performed here constitutes a reference point for the better understanding of the underlying biological networks in the COREAD panel. The resolution and specificity of the protein clusters can be further improved by the combinatorial use of alternative algorithms for construction of biological networks (Allen et al., 2012). Similarly, correlation analysis of protein phosphorylation data demonstrates that functional relationships are encrypted in patterns of co-regulated or anti-regulated phosphorylation events.

### The Impact of Genomic Alterations on Protein Abundance

**Figure 2:**
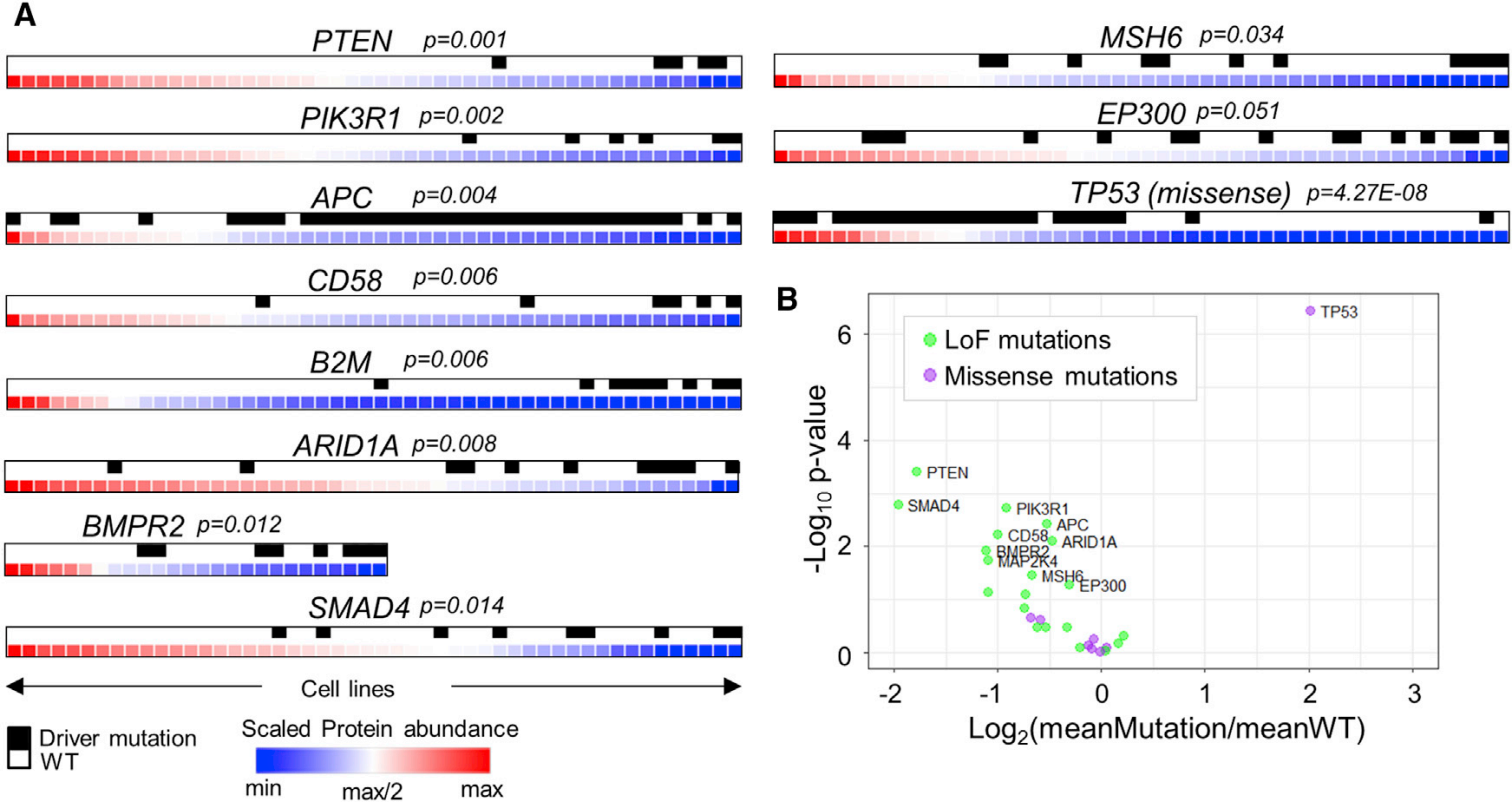
The Effect of Colorectal Cancer Driver Mutations on Protein Abundances (A) Association of driver mutations in colorectal cancer genes with the respective protein abundance levels (ANOVA test; permutation-based FDR < 0.1). The cell lines are ranked by highest (left) to lowest (right) protein abundance, and the bar on the top indicates the presence of driver mutations with black marks. (B) Volcano plot summarizing the effect of loss of function (LoF) and missense driver mutations on the respective protein abundances.

Assessing the impact of non-synonymous protein coding variants and copy number alterations on protein abundance is fundamental in understanding the link between cancer genotypes and dysregulated biological processes. To characterize the impact of genomic alterations on the proteome of the COREAD panel, we first examined whether driver mutations in the most frequently mutated colorectal cancer driver genes (Iorio et al., 2016) could alter the levels of their protein products. For 10 out of 18 such genes harboring driver mutations in at least 5 cell lines (*PTEN*, *PIK3R1*, *APC*, *CD58*, *B2M*, *ARID1A*, *BMPR2*, *SMAD4*, *MSH6*, and *EP300*), we found a significant negative impact on the respective protein abundances, in line with their function as tumor suppressors, whereas missense mutations in *TP53* were associated with elevated protein levels as previously reported (Bertorelle et al., 1996, Dix et al., 1994; ANOVA test; permutation-based FDR < 0.1; Figure 2A). For the majority of driver mutations in oncogenes, there was no clear relationship between the presence of mutations and protein expression (Figure 2B). From these observations, we conclude that mutations in canonical tumor suppressor genes predicted to cause nonsense-mediated decay of transcript generally result in a decrease of protein abundance. This effect, however, varies between the cell lines.

**Figure 3:**
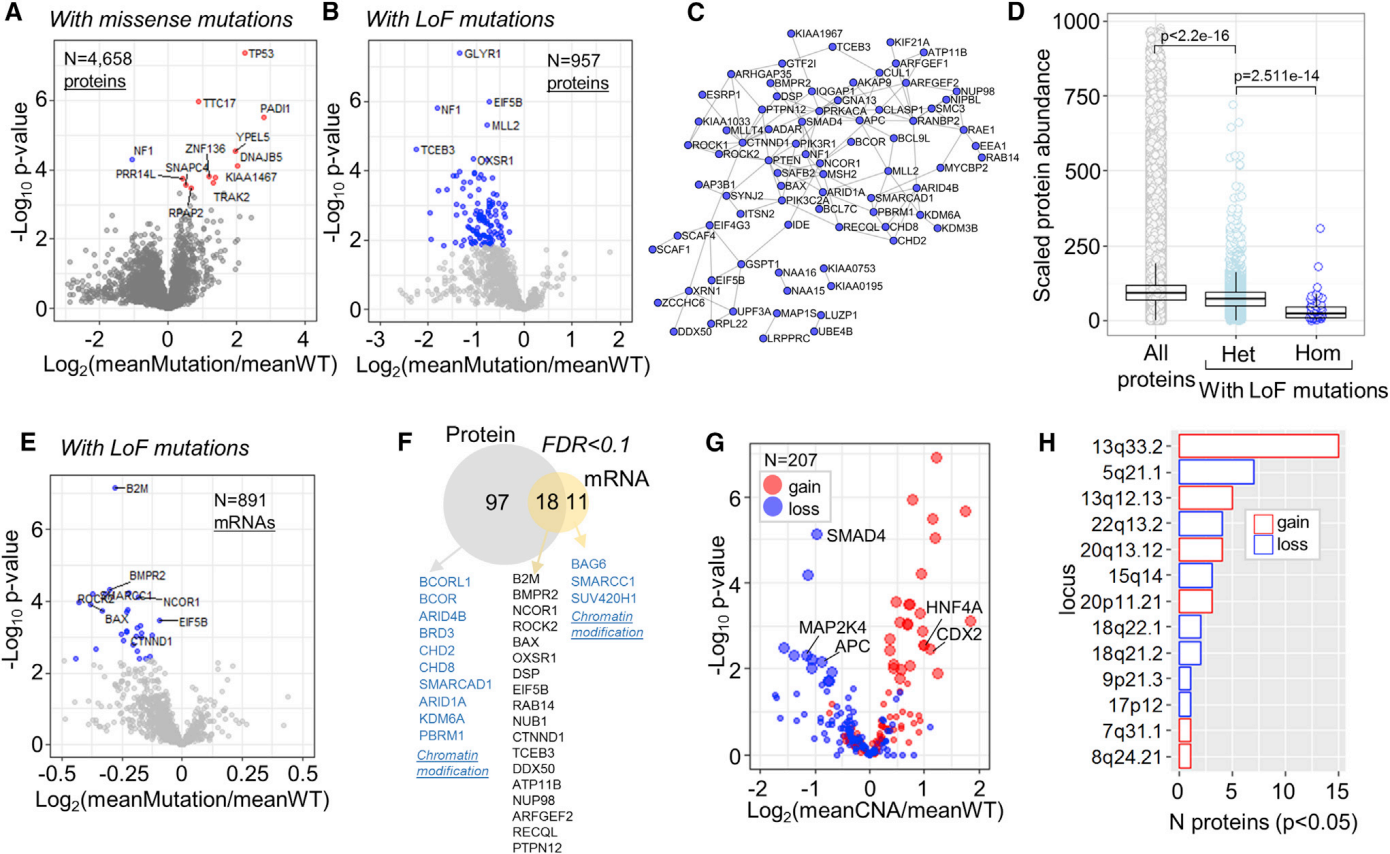
The Global Effects of Genomic Alterations on Protein and mRNA Abundances (A) Volcano plot summarizing the effect of missense mutations on the respective protein abundances (ANOVA test). Hits at permutation-based FDR < 0.1 are colored. (B) Volcano plot summarizing the effect of LoF mutations on the respective protein abundances (ANOVA test). Hits at permutation-based FDR < 0.1 are colored. (C) STRING network of the proteins downregulated by LoF mutations at FDR < 0.1. (D) Boxplots illustrating the protein abundance differences between all proteins and proteins with heterozygous or homozygous LoF mutations. (E) Volcano plot summarizing the effect of LoF mutations with both mRNA and protein measurements on the respective mRNA abundances (ANOVA test). Hits at permutation-based FDR < 0.1 are colored. (F) Venn diagram displaying the overlap between proteins and mRNAs affected by LoF mutations. Selected unique and overlapping proteins are displayed. (G) Volcano plot summarizing the effect of recurrent copy number alterations on the protein abundances of the contained genes (binary data; ANOVA test). Red and blue points highlight genes with amplifications and losses, respectively. Enlarged points highlight genes at permutation-based FDR < 0.1. (H) Bar plot illustrating the number of affected proteins by CNAs per genomic locus. See also Figure S5.

We extended our analysis to globally assess the effect of mutations on protein abundances. For 4,658 genes harboring somatic single-amino-acid substitutions in at least three cell lines, only 12 proteins exhibited differential abundances in the mutated versus the wild-type cell lines at ANOVA test FDR < 0.1 (Figure 3A). Performing the analysis in genes with loss-of-function (LoF) mutations (frameshift, nonsense, in-frame, splice site, and start-stop codon loss) showed that 115 out of the 957 genes tested presented lower abundances in the mutated versus the wild-type cell lines at ANOVA test FDR < 0.1 (Figure 3B). The STRING network of the top significant hits is depicted in Figure 3C and indicates that many of the affected proteins are functionally related. Overall, almost all proteins in a less stringent set with p value < 0.05 (n = 217) were found to be downregulated by LoF mutations, confirming the general negative impact on protein abundances. As expected, zygosity of LoF mutations was a major determinant of protein abundance, with homozygous mutations imposing a more severe downregulation compared to heterozygous mutations (Figure 3D). Whereas the negative impact of LoF mutations was not biased toward their localization in specific protein domains (Figure S5A), we found that mutations localized closer to the protein C terminus were slightly less detrimental (Figure S5B). Notably, genes with LoF mutations and subsequently the significantly affected proteins displayed an overrepresentation of chromatin modification proteins over the identified proteome as the reference set (Fisher’s exact test; Benj. Hoch. FDR < 0.05). Chromatin modifiers play an important role in the regulation of chromatin structure during transcription, DNA replication, and DNA repair (Narlikar et al., 2013). Impaired function of chromatin modifiers can lead to dysregulated gene expression and cancer (Cairns, 2001). Our results show that loss of chromatin modification proteins due to the presence of LoF mutations is frequent among the COREAD cell lines and represents a major molecular phenotype.

A less-pronounced impact of LoF mutations was found at the mRNA level, where only 29 genes (out of 891 with both mRNA and protein data) exhibited altered mRNA abundances in the mutated versus the wild-type cell lines at ANOVA test FDR < 0.1 (Figure 3E). The overlap between the protein and mRNA level analyses is depicted in Figure 3F. Even when we regressed out the mRNA levels from the respective protein levels, almost 40% of the proteins previously found to be significantly downregulated were recovered at ANOVA test FDR < 0.1 and the general downregulation trend was still evident (Figure S5C). On the contrary, regression of protein values out of the mRNA values strongly diminished the statistical significance of the associations between mutations and mRNA levels (Figure S5D). The fact that LoF mutations have a greater impact on protein abundances compared to the mRNA levels suggests that an additional post-transcriptional (e.g., translation efficiency) or a post-translational mechanism (e.g., protein degradation) is involved in the regulation of the final protein abundances. Lastly, 24 of the genes downregulated at the protein level by LoF mutations have been characterized as essential genes in human colon cancer cell lines (OGEE database; Chen et al., 2017). Such genes may be used as targets for negative regulation of cancer cell fitness upon further inhibition.

We also explored the effect of 20 recurrent copy number alterations (CNAs), using binary-type data, on the abundances of 207 quantified proteins falling within these intervals (total coverage 56%). Amplified genes tended to display increased protein levels, whereas gene losses had an overall negative impact on protein abundances with several exceptions (Figure 3G). The 49 genes for which protein abundance was associated with CNAs at ANOVA p value < 0.05 (37 genes at FDR < 0.1) were mapped to 13 genomic loci (Figure 3H), with 13q33.2 amplification encompassing the highest number of affected proteins. Losses in 18q21.2, 5q21.1, and 17p12 loci were associated with reduced protein levels of three important colorectal cancer drivers: SMAD4; APC; and MAP2K4, respectively (FDR < 0.1). Increased levels of CDX2 and HNF4A transcription factors were significantly associated with 13q12.13 and 20q13.12 amplifications (FDR < 0.1). The association of these transcription factors with a number of targets and metabolic processes as found by the co-variome further reveals the functional consequences of the particular amplified loci. All proteins affected by LoF mutations and recurrent CNAs are annotated in Table S1.

Overall, we show that the protein abundance levels of genes with mutations predicted to cause nonsense-mediated mRNA decay are likely to undergo an additional level of negative regulation, which involves translational and/or post-translational events. The extent of protein downregulation heavily depends on zygosity and appears to be independent from secondary structure features and without notable dependency on the position of the mutation on the encoded product. Missense mutations rarely affect the protein abundance levels with the significant exception of TP53. We conclude that only for a small portion of the proteome can the variation in abundance be directly explained by mutations and DNA copy number variations.

### The Consequences of Genomic Alterations Extend to Protein Complexes

**Figure 4:**
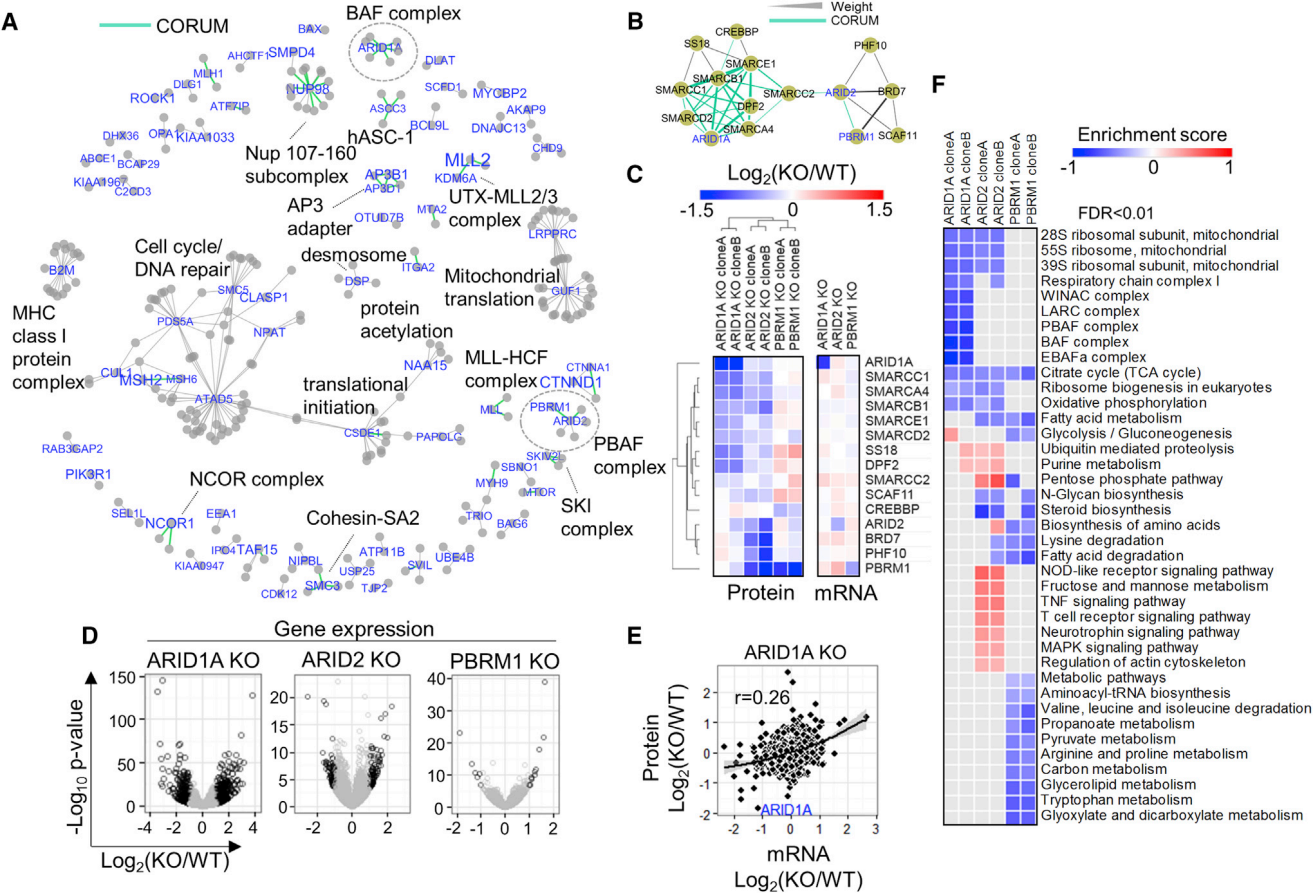
The Consequences of Mutations on Protein Complexes (A) Correlations networks filtered for known STRING interactions of proteins downregulated by LoF mutations at p value < 0.05. The font size is proportional to the −log_10_(p value). CORUM interactions are highlighted as green thick edges, and representative protein complexes are labeled. (B) Protein abundance correlation network of the ARID1A, ARID2, and PBRM1 modules. Green edges denote known CORUM interactions, and the edge thickness is increasing proportionally to the WGCNA interaction weight. (C) Heatmap summarizing the protein and mRNA abundance log_2_fold-change values in the knockout clones compared to the wild-type (WT) clones for the proteins in the ARID1A, ARID2, and PBRM1 modules. (D) Volcano plots highlighting the differentially regulated mRNAs in the KO samples. (E) Scatterplot illustrating the correlation between protein and mRNA abundance changes in the ARID1A KO. (F) KEGG pathway and CORUM enrichment analysis for the proteomic analysis results of ARID1A, ARID2, and PBRM1 CRISPR-cas9 knockouts in human iPSCs.

As tightly controlled maintenance of protein abundance appears to be pivotal for many protein complexes and interactions, we hypothesize that genomic variation can be transmitted from directly affected genes to distant gene protein products through protein interactions, thereby explaining another layer of protein variation. To assess the frequency of such events, we retrieved strongly co-varying interactors of the proteins downregulated by LoF mutations to construct mutation-vulnerable protein networks. For stringency, we filtered for known STRING interactions additionally to the required co-variation. We hypothesize that, in these subnetworks, the downregulation of a protein node due to LoF mutations can also lead to the downregulation of interacting partners. These sub-networks were comprised of 306 protein nodes and 278 interactions and included at least 10 well-known protein complexes (Figure 4A). Two characteristic examples were the BAF and PBAF complexes (Hodges et al., 2016), characterized by disruption of ARID1A, ARID2, and PBRM1 protein abundances. To confirm whether the downregulation of these chromatin-remodeling proteins can affect the protein abundance levels of their co-varying interactors (Figure 4B) post-transcriptionally, we performed proteomics and RNA-seq analysis on CRISPR-Cas9 knockout (KO) clones of these genes in isogenic human iPSCs (Table S6). We found that downregulation of ARID1A protein coincided with diminished protein levels of 7 partners in the predicted network (Figure 4C, left panel). These show the strongest correlations and are known components of the BAF complex (Hodges et al., 2016). In addition, reduced levels of ARID2 resulted in the downregulation of three partners unique to the PBAF complex, with significant loss of PBRM1 protein (Figure 4C, left panel). Several components of the BAF complex were also compromised in the ARID2 KO, reflecting shared components of the BAF and PBAF complexes. Conversely, loss of PBRM1 had no effect on ARID2 protein abundance or any of its module components, in line with the role of PBRM1 in modifying PBAF targeting specificity (Thompson, 2009). The latter demonstrates that collateral effects transmitted through protein interactions can be directional. ARID1A, ARID2, and PBRM1 protein abundance reduction was clearly driven by their respective low mRNA levels; however, the effect was not equally strong in all three genes (Figure 4C, right panel). Strikingly, the interactors that were affected at the protein level were not regulated at the mRNA level, confirming that the regulation of these protein complexes is transcript independent (Figure 4C, right panel). *ARID1A* KO yielded the highest number of differentially expressed genes (Figure 4D); however, these changes were poorly represented in the proteome (Figure 4E). Although pathway-enrichment analysis in all KOs revealed systematic regulation of a wide range of pathways at the protein level, mostly affecting cellular metabolism (Figure 4F), we didn’t identify such regulation at the mRNA level. This suggests that the downstream effects elicited by the acquisition of genomic alterations in the particular genes are distinct between gene expression and protein regulation.

**Figure 5:**
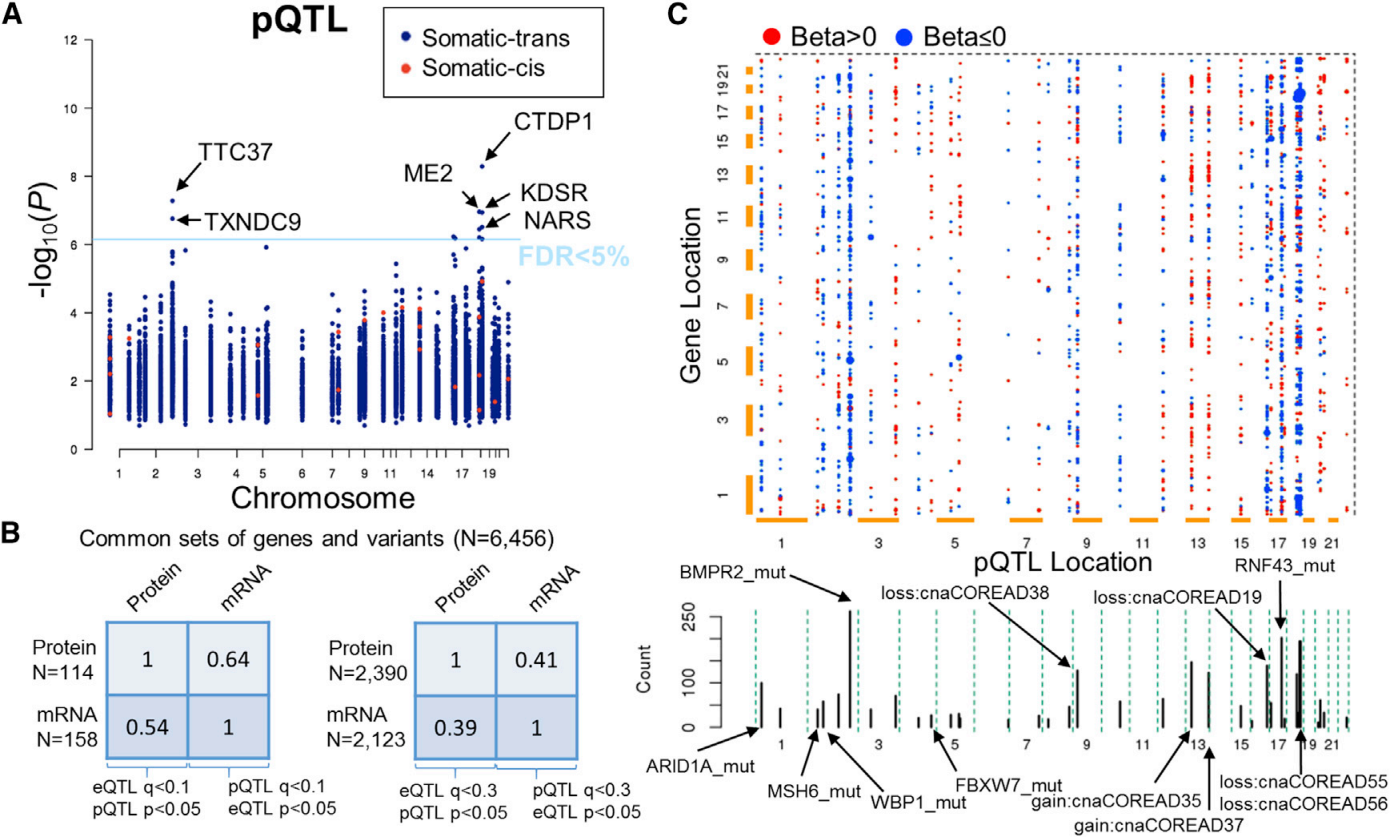
Proteome-wide Quantitative Trait Loci Analysis of Cancer Driver Genomic Alterations (A) Identification of *cis* and *trans* proteome-wide quantitative trait loci (pQTL) in colorectal cancer cell lines considering colorectal cancer driver variants. The p value and genomic coordinates for the most confident non-redundant protein-variant association tests are depicted in the Manhattan plot. (B) Replication rates between independently tested QTL for each phenotype pair using common sets of genes and variants (n = 6,456 genes). (C) Representation of pQTL as 2D plot of variants (x axis) and associated genes (y axis). Associations with q < 0.3 are shown as dots colored by the beta value (blue, negative association; red, positive association) while the size is increasing with the confidence of the association. Cumulative plot of the number of associations per variant is shown below the 2D matrix. See also Figure S5.

The latter prompted us to systematically interrogate the distant effects of all frequent colorectal cancer driver genomic alterations on protein and mRNA abundances by protein and gene expression quantitative trait loci analyses (pQTL and eQTL). We identified 86 proteins and 196 mRNAs with at least one pQTL and eQTL, respectively, at 10% FDR (Figures 5A and S5E). To assess the replication rates between independently tested QTL for each phenotype pair, we also performed the mapping using 6,456 commonly quantified genes at stringent (FDR < 10%) and more relaxed (FDR < 30%) significance cutoffs. In both instances, we found moderate overlap, with 41%–64% of the pQTL validating as eQTLs and 39%–54% of the eQTLs validating as pQTL (Figure 5B). Ranking the pQTL by the number of associations (FDR < 30%) showed that mutations in *BMPR2*, *RNF43*, and *ARID1A*, as well as CNAs of regions 18q22.1, 13q12.13, 16q23.1, 9p21.3, 13q33.2, and 18q21.2 accounted for 62% of the total variant-protein pairs (Figure 5C). The above-mentioned genomic loci were also among the top 10 eQTL hotspots (Figure S5F). High-frequency hotspots in chromosomes 13, 16, and 18 associated with CNAs are consistent with previously identified regions in colorectal cancer tissues (Zhang et al., 2014). We next investigated the pQTL for known associations between the genomic variants and the differentially regulated proteins. Interestingly, increased protein, but not mRNA, levels of the mediator complex subunits were associated with *FBXW7* mutations (Figure S5G), an ubiquitin ligase that targets MED13/13L for degradation (Davis et al., 2013).

Overall, our findings indicate that an additional layer of protein variation can be explained by the collateral effects of mutations on tightly co-regulated partners in protein co-variation networks. Moreover, we show that a large portion of genomic variation affecting gene expression is not directly transmitted to the proteome. Finally, distant protein changes attributed to variation in cancer driver genes can be regulated directly at the protein level with indication of causal effects involving enzyme-substrate relationships.

### Proteomic Subtypes of Colorectal Cancer Cell Lines

To explore whether our deep proteomes recapitulate tissue level subtypes of colorectal cancer and to provide insight into the cellular and molecular heterogeneity of the colorectal cancer cell lines, we performed unsupervised clustering based on the quantitative profiles of the top 30% most variable proteins without missing values (n = 2,161) by class discovery using the ConsensusClusterPlus method (Wilkerson and Hayes, 2010). Optimal separation by k-means clustering was reached using 5 colorectal proteomic subtypes (CPSs) (Figures S6A and S6B).

**Figure 6:**
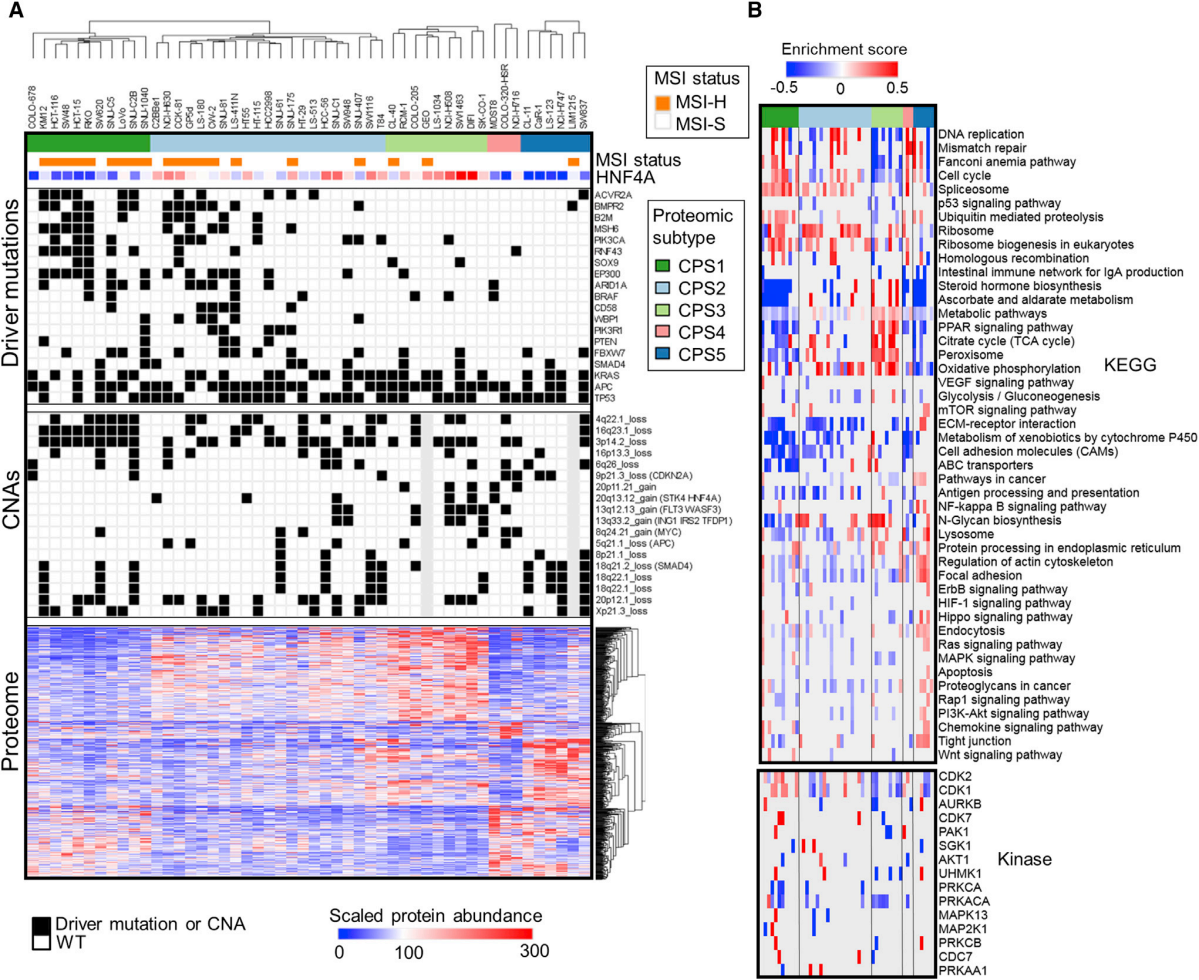
Proteomics Subtypes of Colorectal Cancer Cell Lines and Pathway Analysis (A) Cell lines are represented as columns, horizontally ordered by five color-coded proteomics consensus clusters and aligned with microsatellite instability (MSI), HNF4A protein abundance, cancer driver genomic alterations, and differentially regulated proteins. (B) KEGG pathway and kinase enrichment analysis per cell line. See also Figure S6.

Our proteomic clusters overlapped very well with previously published tissue subtypes and annotations (Medico et al., 2015; Figure S6C), especially with the classification described by De Sousa E Melo et al. (2013). Previous classifiers have commonly subdivided samples along the lines of “epithelial” (lower crypt and crypt top), “microsatellite instability (MSI)-H,” and “stem-like,” with varying descriptions (Guinney et al., 2015). Our in-depth proteomics dataset not only captures the commonly identified classification features but provides increased resolution to further subdivide these groups. The identification of unique proteomic features pointing to key cellular functions gives insight into the molecular basis of these subtypes and provides clarity as to the differences between them (Figures 6A and 6B).

The CPS1 subtype is the canonical MSI-H cluster, overlapping with the CCS2 cluster identified by De Sousa E Melo et al., (2013), CMS1 from Guinney et al., (2015), and CPTAC subtype B (Zhang et al., 2014). Significantly, CPS1 displays low expression of ABC transporters, which may lead to low drug efflux and contribute to the better response rates seen in MSI-H patients (Popat et al., 2005).

Cell lines with a canonical epithelial phenotype (previously classified as CCS1 by De Sousa E Melo et al., 2013) clustered together but are subdivided into 2 subtypes (CPS2 and CPS3). These subtypes displayed higher expression of HNF4A, indicating a more differentiated state. Whereas subtype CPS3 is dominated by transit-amplifying cell phenotypes (Sadanandam et al., 2013), CPS2 is a more heterogeneous group characterized by a mixed TA and goblet cell signature (Figure S6C). CPS2 is also enriched in lines that are hypermutated, including MSI-negative/hypermutated lines (HT115, HCC2998, and HT55; Medico et al., 2015; COSMIC; Figure S6C). However, lower activation of steroid biosynthesis and ascorbate metabolism pathways as well as lower levels of ABC transporters in CPS1 render this group clearly distinguishable from CPS2 (Figure 6B). We also observed subtle differences in the genes mutated between the two groups. *RNF43* mutations and loss of 16q23.1 (including *WWOX* tumor suppressor) are common in CPS1. The separation into two distinct MSI-H/hypermutated classifications was also observed by Guinney et al., (2015) and may have implications for patient therapy and prognosis.

Transit-amplifying subtype CPS3 can be distinguished from CPS2 by lower expression of cell cycle proteins (e.g., CDC20, KIF11, and BUB1); predicted low CDK1, CDK2, and PRKACA kinase activities based on the quantitative profile of known substrates from the PhosphoSitePlus database (Hornbeck et al., 2015); and high PPAR signaling pathway activation (Figure 6B). Common amplifications of 20q13.12 and subsequent high HNF4A levels indicate this cluster corresponds well with CPTAC subtype E (Figure S6D; Zhang et al., 2014). CPS3 also contains lines (DIFI and NCI-H508) that are most sensitive to the anti-epidermal growth factor receptor (EGFR) antibody cetuximab (Medico et al., 2015).

The commonly observed colorectal stem-like subgroup is represented by subtypes CPS4 and CPS5 (Figures 6A and S6C). These cell lines have also been commonly associated with a less-differentiated state by other classifiers, and this is reinforced by our dataset; subtype CPS4 and CPS5 have low levels of HNF4A and CDX1 transcription factors (Chan et al., 2009, Garrison et al., 2006, Jones et al., 2015) and correlate well with CMS4 (Guinney et al., 2015) and CCS3 (De Sousa E Melo et al., 2013). Cells in CPS4 and CPS5 subtypes commonly exhibit loss of the 9p21.3 region, including *CDKN2A* and *CDKN2B*, whereas this is rarely seen in other subtypes. Interestingly, whereas CPS5 displays activation of the Hippo signaling pathway, inflammatory/wounding response, and loss of 18q21.2 (*SMAD4*), CPS4 has a mesenchymal profile, with low expression of CDH1 and JUP similarly to CPTAC subtype C and high Vimentin. Finally, we found common systematic patterns between the COREAD proteomic subtypes and the CPTAC colorectal cancer proteomic subtypes (Zhang et al., 2014) in a global scale (Figures S6D and S6E) using the cell line signature proteins. The overlap between the cell lines and the CPTAC colorectal tissue proteomic subtypes is summarized in Figure S6F.

Lastly, we detected 206 differentially regulated proteins between the MSI-high and MSI-low cell lines (Welch’s t test; permutation based FDR < 0.1; Figure S7A), which were mainly converging to downregulation of DNA repair and chromosome organization as well as to upregulation of proteasome and Lsm2-8 complex (RNA degradation; Figure S7B). Whereas loss of DNA repair and organization functions are the underlying causes of MSI (Boland and Goel, 2010), the upregulation of RNA and protein degradation factors indicate the activation of a scavenging mechanism that regulates the abundance of mutated gene products.

### Pharmacoproteomic Models Significantly Contribute to Drug Response Prediction

Although a number of recent studies have investigated the power of different combinations of molecular data to predict drug response in colorectal cancer cell lines, these have been limited to using genomic (mutations and copy number), transcriptomic, and methylation datasets (Iorio et al., 2016). We have shown above that the DNA and gene expression variations are not directly consistent with the protein measurements. Also, it has been shown that there is a gain in predictive power for some phenotypic associations when also using protein abundance and phosphorylation changes (Costello et al., 2014, Gholami et al., 2013, Li et al., 2017). To date, there has not been a comprehensive analysis of the effect on the predictive power from the addition of proteomics datasets in colorectal cancer. All of the colorectal cell lines included in this study have been extensively characterized by sensitivity data (half maximal inhibitory concentration [IC_50_] values) for 265 compounds (Iorio et al., 2016). These include clinical drugs (n = 48), drugs currently in clinical development (n = 76), and experimental compounds (n = 141).

**Figure 7:**
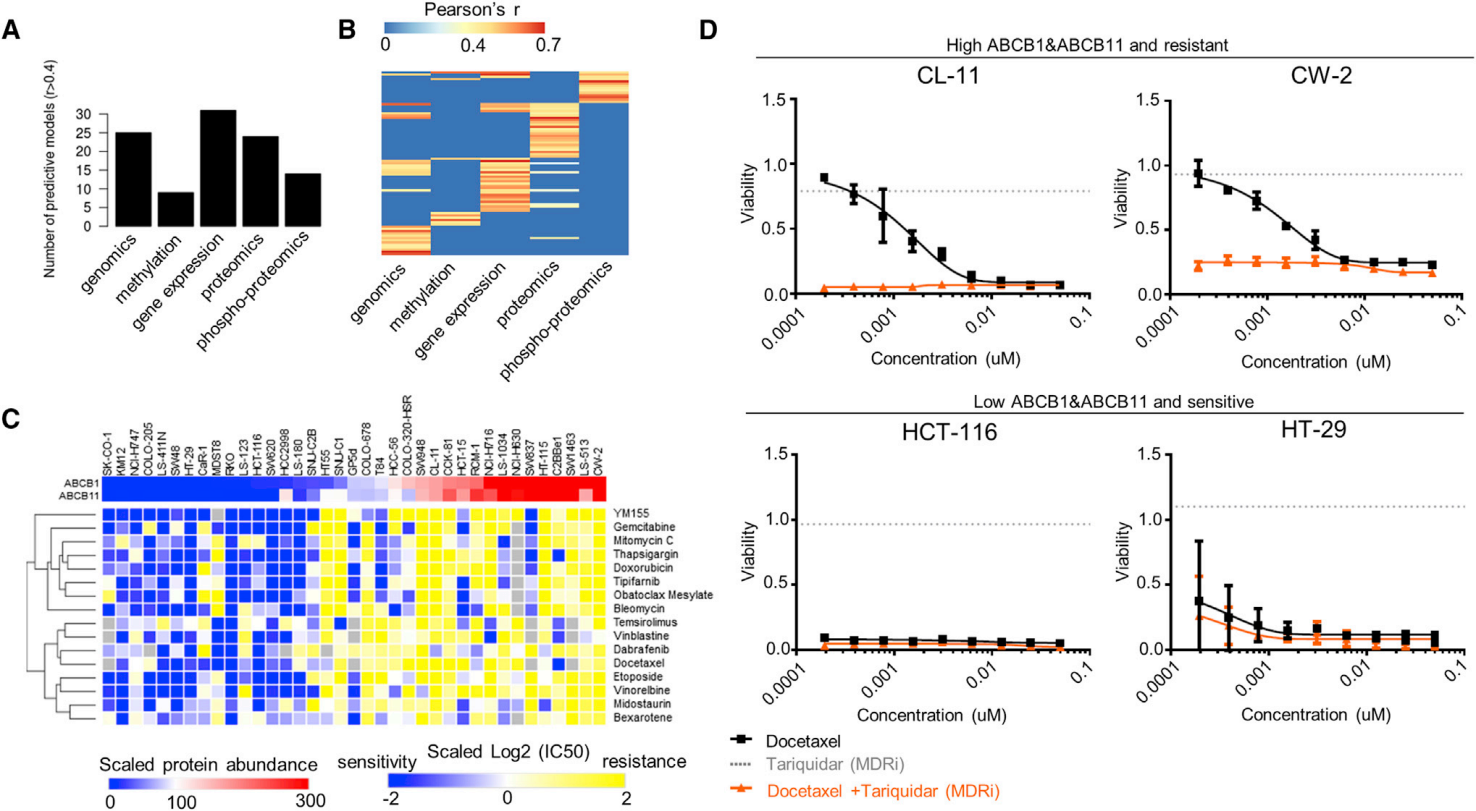
Pharmacoproteomic Models (A) The number of drugs for which predictive models (i.e., models where the Pearson correlation between predicted and observed IC_50_s exceeds r > 0.4) could be fitted is stratified per data type. (B) Heatmap indicating for each drug and each data type whether a predictive model could be fitted. Most drugs were specifically predicted by one data type. (C) Heatmap of scaled log2 IC_50_ values for selected drugs displaying significant association (ANOVA FDR < 0.05) between protein abundance of ABCB1, ABCB11, and drug response. (D) Dose-response profiles for colorectal cancer cell lines treated with docetaxel (black line), 2.5 μM tariquidar alone (gray dotted line), or the combination of docetaxel and 2.5 μM tariquidar (orange line). Error bars represent mean ± SEM. See also Figure S7.

We built Elastic Net models that use as input features genomic (mutations and copy number gains/losses), methylation (CpG islands in gene promoters), gene expression, proteomics, and phosphoproteomics datasets. We were able to generate predictive models where the Pearson correlation between predicted and observed IC_50_ was greater than 0.4 in 81 of the 265 compounds (Table S7). Response to most drugs was often specifically predicted by one data type, with very little overlap (Figures 7A and 7B, respectively). The number of predictive models per drug target pathway and data type is depicted in Figure S7C, highlighting the contribution of proteomics and phosphoproteomics datasets in predicting response to certain drug classes.

Within the proteomics-based signatures found to be predictive for drug response, we frequently observed the drug efflux transporters ABCB1 and ABCB11 (6 and 6 out of 24, respectively; 8 non-redundant; Table S7). In all models containing these proteins, elevated expression of the drug transporter was associated with drug resistance, in agreement with previous results (Garnett et al., 2012). Notably, protein measurements of these transporters correlated more strongly with response to these drugs than the respective mRNA measurements (mean Pearson’s r = 0.61 and r = 0.31, respectively; Wilcoxon test p value = 0.016). Interestingly, ABCB1 and ABCB11 are tightly co-regulated (Pearson’s r = 0.92), suggesting a novel protein interaction. Classifying the cell lines into two groups with low and high mean protein abundance of ABCB1 and ABCB11 revealed a strong overlap with drug response for 54 compounds (ANOVA test; permutation-based FDR < 0.05). Representative examples of these drug associations are shown in Figure 7C. To confirm the causal association between the protein abundance levels of ABCB1, ABCB11, and drug response, we performed viability assays in four cell lines treated with docetaxel, a chemotherapeutic agent broadly used in cancer treatment. The treatments were performed in the presence or absence of an ABCB1 inhibitor (tariquidar) and confirmed that ABCB1 inhibition increases sensitivity to docetaxel (Figure 7D) in the cell lines with high ABCB1 and ABCB11 levels. Given the dominant effect of the drug efflux proteins in drug response, we next tested whether additional predictive models could be identified by correcting the drug response data for the mean protein abundance of ABCB1 and ABCB11 using linear regression. With this analysis, we were able to generate predictive models for 41 additional drugs (total 57) from all input datasets combined (Figure S7D; Table S7). Taken together, our results show that the protein expression levels of drug efflux pumps play a key role in determining drug response, and whereas predictive genomic biomarkers may still be discovered, the importance of proteomic associations with drug response should not be underestimated.

## Discussion

Our analysis of colorectal cancer cells using in-depth proteomics has yielded several significant insights into both fundamental molecular cell biology and the molecular heterogeneity of colorectal cancer subtypes. Beyond static measurements of protein abundances, the quality of our dataset enabled the construction of a reference proteomic co-variation map with topological features capturing the interplay between known protein complexes and biological processes in colorectal cancer cells. We show that the subunits of protein complexes tend to tightly maintain their total abundance ratios post-transcriptionally, and this is a fundamental feature of the co-variation network. The primary level of co-variation between proteins enables the generation of unique abundance profiles of known protein interactions, and the secondary level of co-regulation between protein complexes can indicate the formation of multi-complex protein assemblies. Moreover, the identification of proteins with outlier profiles from the conserved profile of their known interactors within a given complex can point to their pleiotropic roles in the associated processes. Notably, our approach can be used in combination with high-throughput pull-down assays (Hein et al., 2015, Huttlin et al., 2015) for further refinement of large-scale protein interactomes based on co-variation signatures that appear to be pivotal for many protein interactions. Additionally, our approach can serve as a time-effective tool for the identification of tissue-specific co-variation profiles in cancer that may reflect tissue-specific associations. As a perspective, our data may be used in combination with genetic interaction screens (Costanzo et al., 2016) to explore whether protein co-regulation can explain or predict synthetic lethality (Kaelin, 2005). Another novel aspect that emerged from our analysis is the maintenance of co-regulation at the level of net protein phosphorylation. This seems to be more pronounced in signaling pathways, where the protein abundances are insufficient to indicate functional associations. Analogous study of co-regulation between different types of protein modifications could also enable the identification of modification cross-talk (Beltrao et al., 2013). This framework also enabled the identification of upstream regulatory events that link transcription factors to their transcriptional targets at the protein level and partially explained the components of the co-variome that are not strictly shaped by physical protein interactions. To a smaller degree, the module-based analysis was predictive of DNA copy number variations, exposing paradigms of simple cause-and-effect proteogenomic features of the cell lines. Such associations should be carefully taken into consideration in large-scale correlation analyses, as they do not necessarily represent functional relationships.

The simplification of the complex proteomic landscapes into co-variation modules enables a more direct alignment of genomic features with cellular functions and delineates how genomic alterations affect the proteome directly and indirectly. We show that LoF mutations can have a direct negative impact on protein abundances further to mRNA regulation. Targeted deletion of key chromatin modifiers by CRISPR/cas9 followed by proteomics and RNA-seq analysis confirmed that the effects of genomic alterations can propagate through physical protein interactions, highlighting the role of translational or post-translational mechanisms in modulating protein co-variation. Additionally, our analysis indicated that directionality can be another characteristic of such interactions.

We provide evidence that colorectal cancer subtypes derived from tissue level gene expression and proteomics datasets are largely recapitulated in cell-based model systems at the proteome level, which further resolves the main subtypes into groups. This classification reflects a possible cell type of origin and the underlying differences in genomic alterations. This robust functional characterization of the COREAD cell lines can guide cell line selection in targeted cellular and biochemical experimental designs, where cell-line-specific biological features can have an impact on the results. Proteomic analysis highlighted that the expression of key protein components, such as ABC transporters, is critical in predicting drug response in colorectal cancer. Whereas further work is required to establish these as validated biomarkers of patient response in clinical trials, numerous studies have noted the role of these channels in aiding drug efflux (Chen et al., 2016). In summary, this study demonstrates the utility of proteomics in different aspects of systems biology and provides a valuable insight into the regulatory variation in colorectal cancer cells.

## Experimental Procedures

### Sample Preparation and Analysis

Cell pellets were lysed by probe sonication/boiling, and protein extracts were subjected to trypsin digestion. The tryptic peptides were labeled with the TMT10plex reagents, combined at equal amounts, and fractionated with high-pH C18 high-performance liquid chromatography (HPLC). Phosphopeptide enrichment was performed with immobilized metal ion affinity chromatography (IMAC). LC-MS analysis was performed on the Dionex Ultimate 3000 system coupled with the Orbitrap Fusion Mass Spectrometer. MS3 level quantification with Synchronous Precursor Selection was used for total proteome measurements, whereas phosphopeptide measurements were obtained with a collision-induced dissociation-higher energy collisional dissociation (CID-HCD) method at the MS2 level. Raw mass spectrometry files were subjected to database search and quantification in Proteome Discoverer 1.4 or 2.1 using the SequestHT node followed by Percolator validation. Protein and phosphopeptide quantification was obtained by the sum of column-normalized TMT spectrum intensities followed by row-mean scaling.

### Statistical Analysis

Enrichment for biological terms, pathways, and kinases was performed in Perseus 1.4 software with Fisher’s test or with the 1D-annotation-enrichment method. Known kinase-substrate associations were downloaded from the PhosphoSitePlus database. All terms were filtered for Benjamini-Hochberg FDR < 0.05 or FDR < 0.1. Correlation analyses were performed in RStudio with Benjamini-Hochberg multiple testing correction. ANOVA and Welch’s tests were performed in Perseus 1.4 software. Permutation-based FDR correction was applied to the ANOVA test p values for the assessment of the impact of mutations and copy number variations on protein and mRNA abundances. Volcano plots, boxplots, distribution plots, scatterplots, and bar plots were drawn in RStudio with the ggplot2 and ggrepel packages. All QTL associations were implemented by LIMIX using a linear regression test.

## Author Contributions

Conceptualization, J.S.C. and U.M.; Methodology, T.I.R., S.P.W., and J.S.C.; Cell Lines, S.P. and S.P.W.; Mass Spectrometry, T.I.R. and L.Y.; Data Analysis, T.I.R., E.G., F.Z.G., S.P.W., J.C.W., M.P., P.B., J.S.-R., A.B., and O.S.; Cell Lines Classification, R.D. and J.G.; Drug Data Analysis, N.A., M.M., M.S., M.Y., J.S.-R., S.P.W., T.I.R., L.W., and U.M.; CRISPR Lines RNA-Seq, C.A., M.D.C.V.-H., and D.J.A.; Writing – Original Draft, T.I.R., S.P.W., L.W., U.M., and J.S.C.; Writing – Review and Editing, all.

## Accession Numbers

The accession number for the mass spectrometry proteomics data reported in this paper is PRIDE: PXD005235. The accession number for the CRISPR-cas9 RNA-seq data reported in this paper is European Nucleotide Archive: EGAS00001002262. The accession number for the quantitative proteomics data reported in this paper is Expression Atlas: E-PROT-6.

## Supplemental Information

Document S1. Supplemental Experimental Procedures and Figures S1–S7

Table S1. Relative Protein Abundances in the COREAD Cell Lines, Related to Figures 1, 2, 3, 5, 6, and 7 Scaled protein quantification values for 50 colorectal cancer cell lines. Proteins affected by mutations or DNA copy number alterations are annotated by ANOVA p value and average log2fold-changes where applicable.

Table S2. Relative Phosphopeptide Abundances in the COREAD Cell Lines, Related to Figures 6, S2, and S4 Scaled phosphopeptide quantification values for 50 colorectal cancer cell lines. Phosphopeptides are annotated by the known regulatory kinase and by KEGG pathway where applicable. The values are not corrected or normalized for total protein levels.

Table S3. Correlation of Proteins belonging to Known CORUM Complexes, Related to Figure 1 Overlap of known CORUM protein complexes with the COREAD cell lines proteome. The mean and median correlation between protein complex components is shown for all complexes. Proteins with outlier profiles (high or low correlations) are labelled.

Table S4. Annotation of WGCNA Modules, Related to Figure 1 Enrichment of Gene Ontology, GSEA, KEGG, CORUM and Pfam terms for the WGCNA modules. Significance FDR is shown for each term.

Table S5. The Full WGCNA Network, Related to Figure 1 The entire WGCNA correlation network with weights greater than 0.02. The respective Pearson’s correlations are also shown and CORUM interactions are highlighted (value=1). Modules that are possibly driven by DNA copy number variations are highlighted (value=1).

Table S6. Proteomics and RNA-Seq Results for the ARID1A, ARID2, and PBRM1 CRISPR-cas9 Experiments, Related to Figure 4 Absolute and scaled S/N TMT values are shown for the proteomics data. ANOVA pvalues refer to any comparison between the WT, ARID1A, ARID2 and PBRM1 replicate groups. RNA-seq quantification and significance is shown only for the respective identified proteins.

Table S7. Associations between Drug Response and Molecular Data, Related to Figures 7 and S7 The predictive performance, data type and target pathway is shown for each association. The column “Corrected for ABCBs?” indicates whether the feature was predicted with the mean protein abundance of ABCB1 and ABCB11 regressed-out from drug response data.

Document S2. Article plus Supplemental Information
