# Supplementary material for: Genomic Determinants of Protein Abundance Variation in Colorectal Cancer Cells
Source: Cell Rep. 2017 Aug 29;20(9):2201–14. doi: 10.1016/j.celrep.2017.08.010 (PMC5583477; doi:10.1016/j.celrep.2017.08.010)
Supplement: Document S1. Supplemental Experimental Procedures and Figures S1–S7 [file mmc1.pdf]

Cell Reports, Volume *20*

## Supplemental Information

# Genomic Determinants of Protein Abundance Variation in Colorectal Cancer Cells

**Theodoros I. Roumeliotis, Steven P. Williams, Emanuel Gonçalves, Clara Alsinet, Martin Del Castillo Velasco-Herrera, Nanne Aben, Fatemeh Zamanzad Ghavidel, Magali Michaut, Michael Schubert, Stacey Price, James C. Wright, Lu Yu, Mi Yang, Rodrigo Dienstmann, Justin Guinney, Pedro Beltrao, Alvis Brazma, Mercedes Pardo, Oliver Stegle, David J. Adams, Lodewyk Wessels, Julio Saez-Rodriguez, Ultan McDermott, and Jyoti S. Choudhary**

## Supplemental Figures

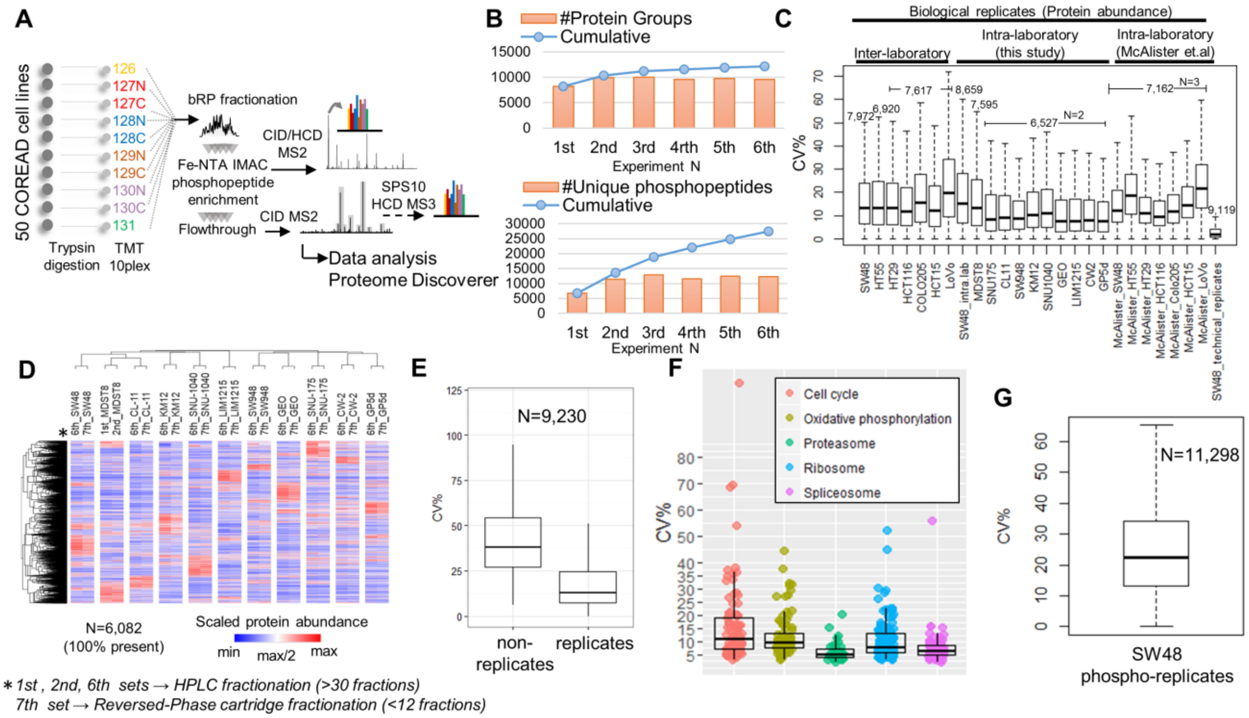


**Figure S1. Proteome and phosphoproteome coverage and reproducibility Related to Figure 1.** A) Workflow for quantitative global proteome and phosphoproteome analysis. 50 colorectal cancer cell lines (COREAD) were analysed using TMT-10plex in seven multiplex sets. The SW48 cell line was included in each set. Biological replicates of the MDST8 cell line were included in two different sets and the 7th set corresponds to a biological replicate of the 6th set. A technical replicate of the SW48 cell line was included in the 5th set. These were used to evaluate the normalization and the batch effect correction methods. B) Number of protein groups (top panel) and unique phosphopeptides (bottom panel) identified per multiplex set are depicted as orange bars and cumulative numbers are shown as blue lines. C) Boxplots summarizing the coefficient of variation (CV%) of protein abundance measurements for intra-laboratory and inter-laboratory comparisons using biological and technical replicates. D) Heatmap of relative protein abundances between biological replicates for 11 cell lines without missing values. E) Boxplots illustrating the CV% of protein abundance measurements between replicate and non-replicate (all different cell lines) samples across a panel of 11 cell lines. F) Boxplots summarizing the CV% of protein abundance measurements for selected KEGG pathways. G) Boxplot illustrating the CV% of protein phosphorylation measurements for the SW48 biological replicates.

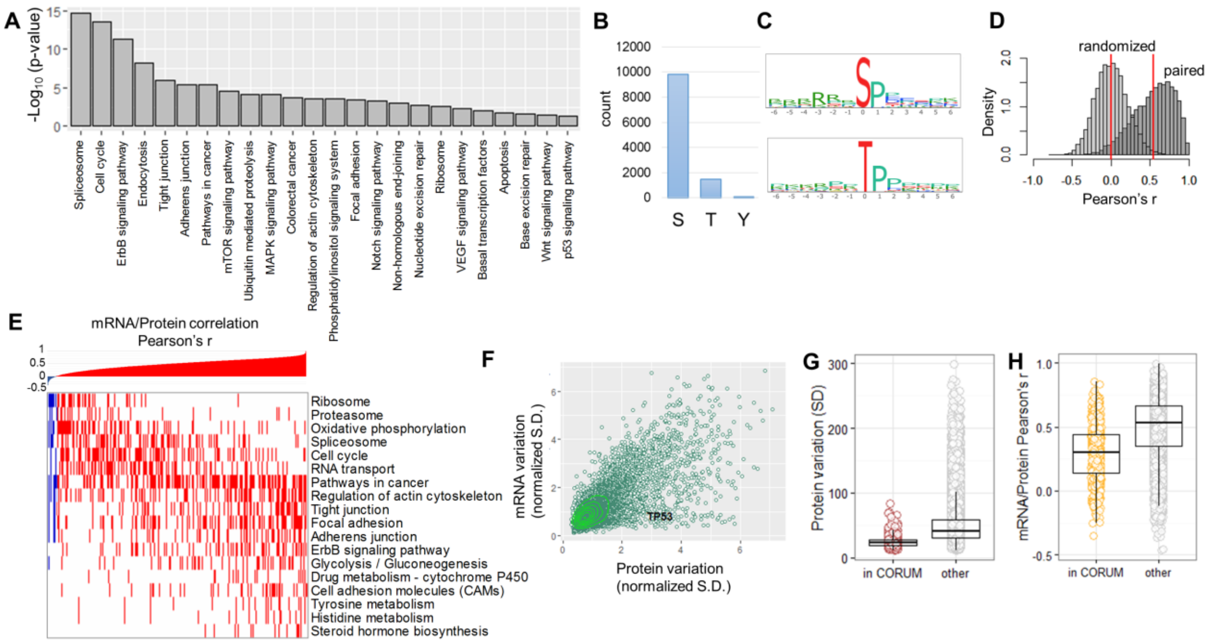


**Figure S2**. **Qualitative and quantitative characteristics of the phosphoproteome and global mRNA-to-protein comparisons Related to Figures 1 and 6.** A) Enriched KEGG pathways by DAVID analysis of all quantified phosphoproteins. B) Number of S,T and Y phosphorylated residues quantified. C) Logos of the phosphorylation motifs identified. D) The distributions of Pearson coefficients for randomized and matched pairs of phosphopeptide abundances versus protein abundances. E) Gene-level mRNA-to-protein Pearson correlations ranked by lowest to highest value and KEGG pathway enrichment for low and high correlations. F) Scatter plot of mRNA versus protein variation using normalized standard deviation values across the COREAD cell lines. G) Boxplots for protein abundance variation of proteins with high correlations within CORUM complexes versus all other proteins. H) Boxplots for mRNA/protein correlation of proteins with high correlations within CORUM complexes versus all other proteins.

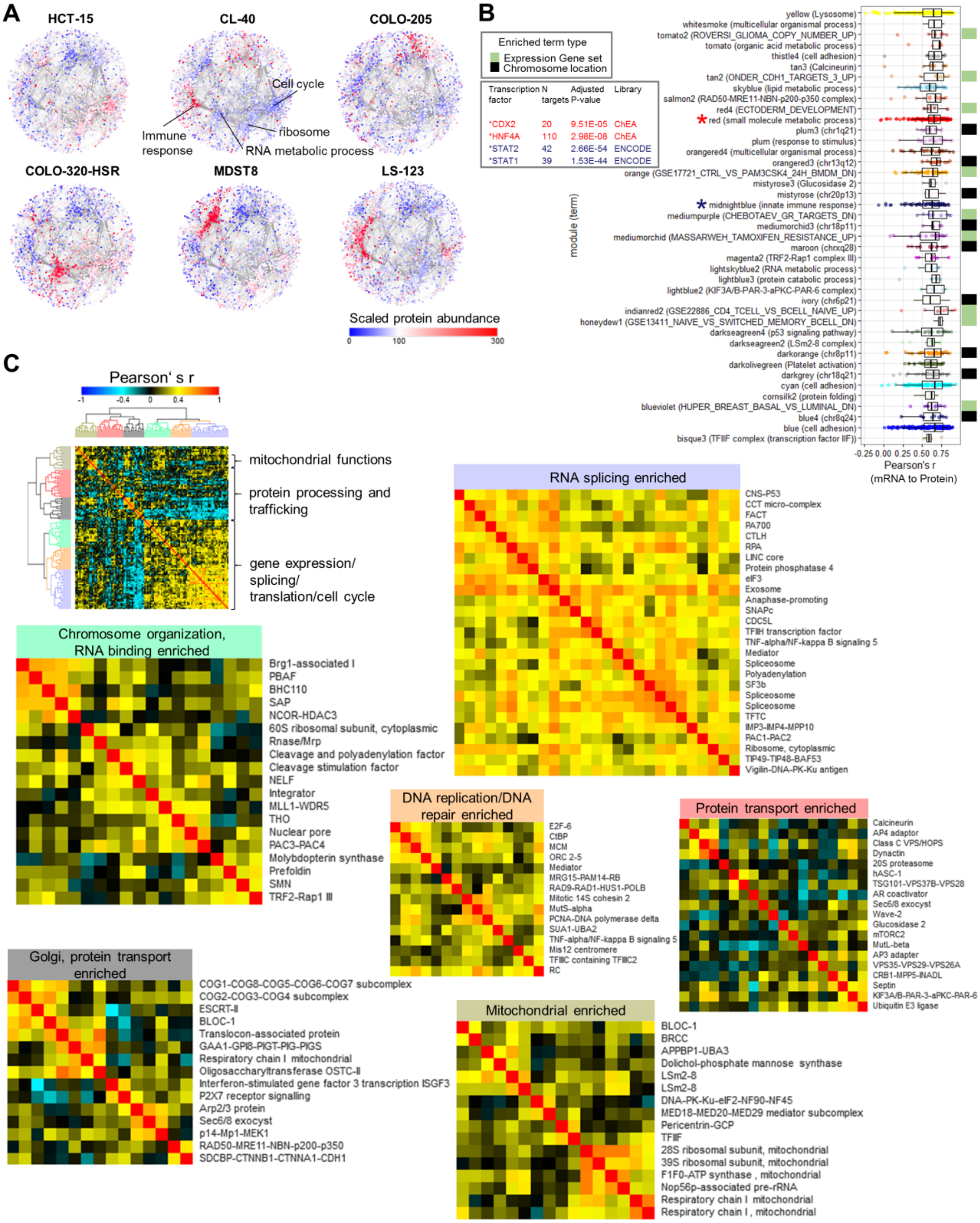
A
HCT-15
CL-40
COLO-205
Cell cycle
Immune response
ribosome
RNA metabolic process
COLO-320-HSR
MDST8
LS-123
Scaled protein abundance
0
100
300
B
Enriched term type
Expression Gene set
Chromosome location
Transcription factor | N targets | Adjusted P-value | Library
*CDX2 | 20 | 9.51E-05 | ChEA
*HNF4A | 110 | 2.98E-08 | ChEA
*STAT2 | 42 | 2.66E-54 | ENCODE
*STAT1 | 39 | 1.53E-44 | ENCODE
module (term)
yellow (Lysosome)
whitesmoke (multicellular organismal process)
tomato2 (ROVERSI_GLIOMA_COPY_NUMBER_UP)
tomato (organic acid metabolic process)
thistle4 (cell adhesion)
tan3 (Calcineurin)
tan2 (ONDER_CDH1_TARGETS_3_UP)
skyblue (lipid metabolic process)
salmon2 (RAD50-MRE11-NBN-p200-p350 complex)
red4 (ECTODERM_DEVELOPMENT)
* red (small molecule metabolic process)
plum3 (chr1q21)
plum (response to stimulus)
orangered4 (multicellular organismal process)
orangered3 (chr13q12)
orange (GSE17721_CTRL_VS_PAM3CSK4_24H_BMDM_DN)
mistyrose3 (Glucosidase 2)
mistyrose (chr20p13)
* midnightblue (innate immune response)
mediumpurple (CHEBOTAEV_GR_TARGETS_DN)
mediumorchid3 (chr18p11)
mediumorchid (MASSARWEH_TAMOXIFEN_RESISTANCE_UP)
maroon (chrxq28)
magenta2 (TRF2-Rap1 complex III)
lightskyblue2 (RNA metabolic process)
lightblue3 (protein catabolic process)
lightblue2 (KIF3A/B-PAR-3-aPKC-PAR-6 complex)
ivory (chr6p21)
indianred2 (GSE22886_CD4_TCELL_VS_BCELL_NAIVE_UP)
honeydew1 (GSE13411_NAIVE_VS_SWITCHED_MEMORY_BCELL_DN)
darkseagreen4 (p53 signaling pathway)
darkseagreen2 (LSm2-8 complex)
darkorange (chr8p11)
darkolivegreen (Platelet activation)
darkgrey (chr18q21)
cyan (cell adhesion)
cornsilk2 (protein folding)
blueviolet (HUPER_BREAST_BASAL_VS_LUMINAL_DN)
blue4 (chr8q24)
blue (cell adhesion)
bisque3 (TFIIF complex (transcription factor IIF))
-0.25 0.00 0.25 0.50 0.75
Pearson's r
(mRNA to Protein)
C
Pearson' s r
-1
-0.4
0
0.4
1
mitochondrial functions
protein processing and trafficking
gene expression/ splicing/ translation/cell cycle
RNA splicing enriched
CNS-P53
CCT micro-complex
FACT
PA700
CTLH
RPA
LINC core
Protein phosphatase 4
eIF3
Exosome
Anaphase-promoting
SNAPc
CDC5L
TFIIH transcription factor
TNF-alpha/NF-kappa B signaling 5
Mediator
Spliceosome
Polyadenylation
SF3b
Spliceosome
Spliceosome
TFTC
IMP3-IMP4-MPP10
PAC1-PAC2
Ribosome, cytoplasmic
TIP49-TIP48-BAF53
Vigilin-DNA-PK-Ku antigen
Chromosome organization, RNA binding enriched
Brg1-associated I
PBAF
BHC110
SAP
NCOR-HDAC3
60S ribosomal subunit, cytoplasmic
Rnase/Mrp
Cleavage and polyadenylation factor
Cleavage stimulation factor
NELF
Integrator
MLL1-WDR5
THO
Nuclear pore
PAC3-PAC4
Molybdopterin synthase
Prefoldin
SMN
TRF2-Rap1 III
DNA replication/DNA repair enriched
E2F-6
CtBP
MCM
ORC 2-5
Mediator
MRG15-PAM14-RB
RAD9-RAD1-HUS1-POLB
Mitotic 14S cohesin 2
MutS-alpha
PCNA-DNA polymerase delta
SUA1-UBA2
TNF-alpha/NF-kappa B signaling 5
Mis12 centromere
TFIIIC containing TFIIIC2
RC
Protein transport enriched
Calcineurin
AP4 adaptor
Class C VPS/HOPS
Dynactin
20S proteasome
hASC-1
TSG101-VPS37B-VPS28
AR coactivator
Sec6/8 exocyst
Wave-2
Glucosidase 2
mTORC2
MutL-beta
AP3 adapter
VPS35-VPS29-VPS26A
CRB1-MPP5-INADL
Septin
KIF3A/B-PAR-3-aPKC-PAR-6
Ubiquitin E3 ligase
Golgi, protein transport enriched
COG1-COG8-COG5-COG6-COG7 subcomplex
COG2-COG3-COG4 subcomplex
ESCRT-II
BLOC-1
Translocon-associated protein
GAA1-GPI8-PIGT-PIG-PIGS
Respiratory chain I mitochondrial
Oligosaccharyltransferase OSTC-II
Interferon-stimulated gene factor 3 transcription ISGF3
P2X7 receptor signalling
Arp2/3 protein
Sec6/8 exocyst
p14-Mp1-MEK1
RAD50-MRE11-NBN-p200-p350
SDCBP-CTNNB1-CTNNA1-CDH1
Mitochondrial enriched
BLOC-1
BRCC
APPBP1-UBA3
Dolichol-phosphate mannose synthase
LSm2-8
LSm2-8
DNA-PK-Ku-eIF2-NF90-NF45
MED18-MED20-MED29 mediator subcomplex
Pericentrin-GCP
TFIIF
28S ribosomal subunit, mitochondrial
39S ribosomal subunit, mitochondrial
F1F0-ATP synthase , mitochondrial
Nop56p-associated pre-rRNA
Respiratory chain I mitochondrial
Respiratory chain I , mitochondrial

**Figure S3. The colorectal cancer cell protein correlation network Related to Figure 1.** A) Examples of unique network signatures for six COREAD cell lines. B) Boxplots of Pearson mRNA-to-protein correlation for modules highly corresponding to mRNA levels. Dots are color-coded according to the default WGCNA module name. Modules enriched for GSEA gene sets and chromosome locations are highlighted with green and black marks respectively. Enriched transcription factors in the "small molecule metabolic process" and "innate immune response" modules are displayed. C) Correlation heatmap of protein complexes based on the similarities of their representative profiles (eigengenes). The heatmap is divided in six main clusters which are color-coded and magnified. Shortened CORUM complex names are used. Duplicate entries represent protein complexes that are separated into more than one modules.

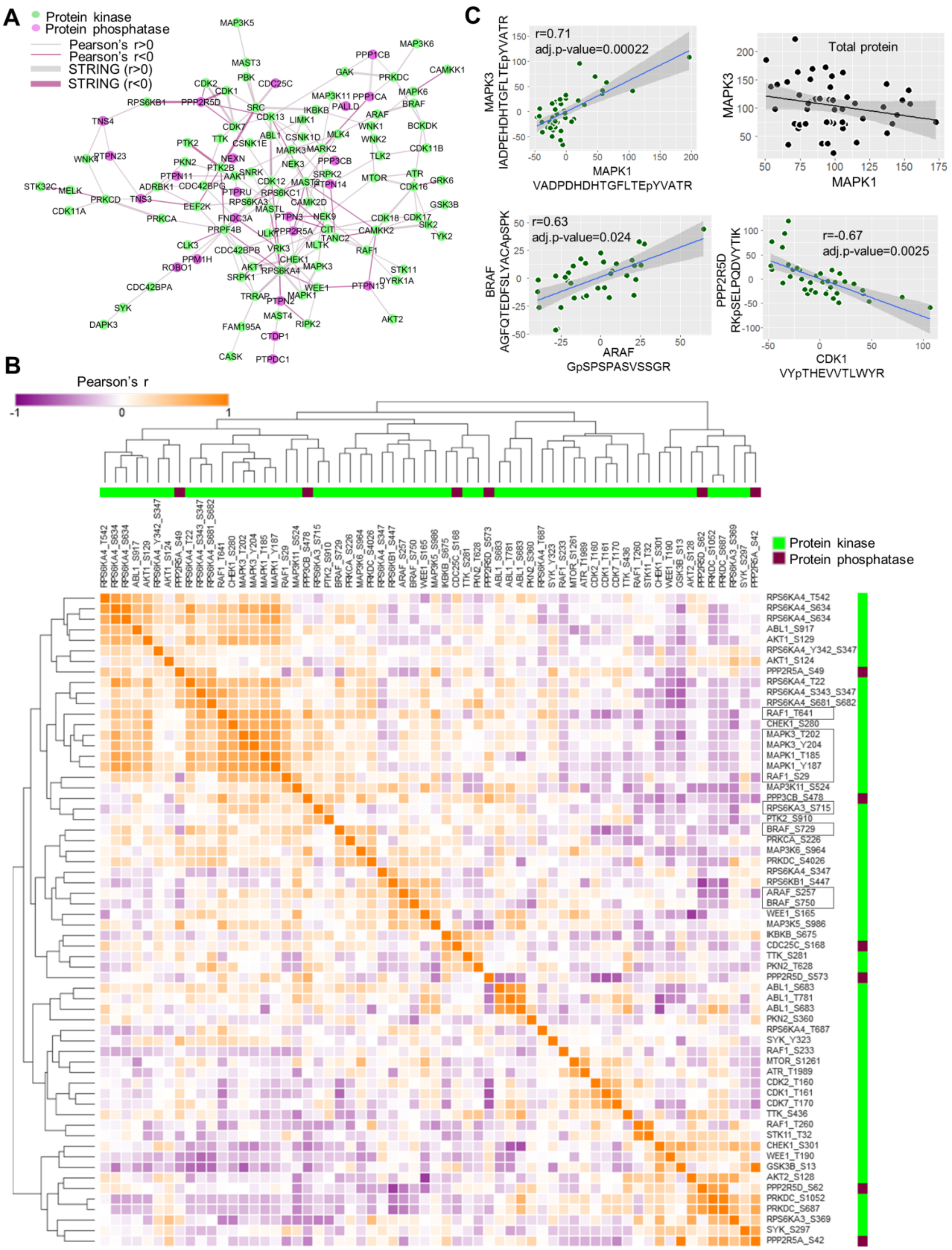

A
Protein kinase
Protein phosphatase
Pearson's r>0
Pearson's r<0
STRING (r>0)
STRING (r<0)
C
r=0.71
adj.p-value=0.00022
MAPK3
IADPEHDHTGFLTEpYVATR
MAPK1
VADPDHDHTGFLTEpYVATR
Total protein
MAPK3
MAPK1
r=0.63
adj.p-value=0.024
BRAF
AGFQTEDFSLYACApSPK
ARAF
GpSPSPASVSSGR
r=-0.67
adj.p-value=0.0025
PPP2R5D
RKpSELPQDVYTIK
CDK1
VYpTHEVVTLWYR
B
Pearson's r
-1
0
1
Protein kinase
Protein phosphatase

**Figure S4**. ***De novo* prediction of a phosphorylation network involving kinases and phosphatases Related to Figure 6.** A) Correlation network of positive and negative associations (Benj. Hoch. FDR<0.1) among phosphopeptides belonging to protein kinases and phosphatases. The nodes represent the phosphorylated kinases or phosphatases. B) Correlation heatmap of phosphopeptides belonging to kinases and phosphatases involved in KEGG signalling pathways. Duplicate entries represent protein phosphorylation sites that were identified by different overlapping phosphopeptides with different lengths. Phosphoproteins of the MAPK pathway are outlined. C) Correlation plots of MAPK1 and MAPK3 phosphorylation (top left) and total protein (top right). Correlation plots of BRAF and ARAF phosphorylation (bottom left) and negative correlation plot between phosphorylated CDK1 kinase and PPP2R5D phosphatase (bottom right).

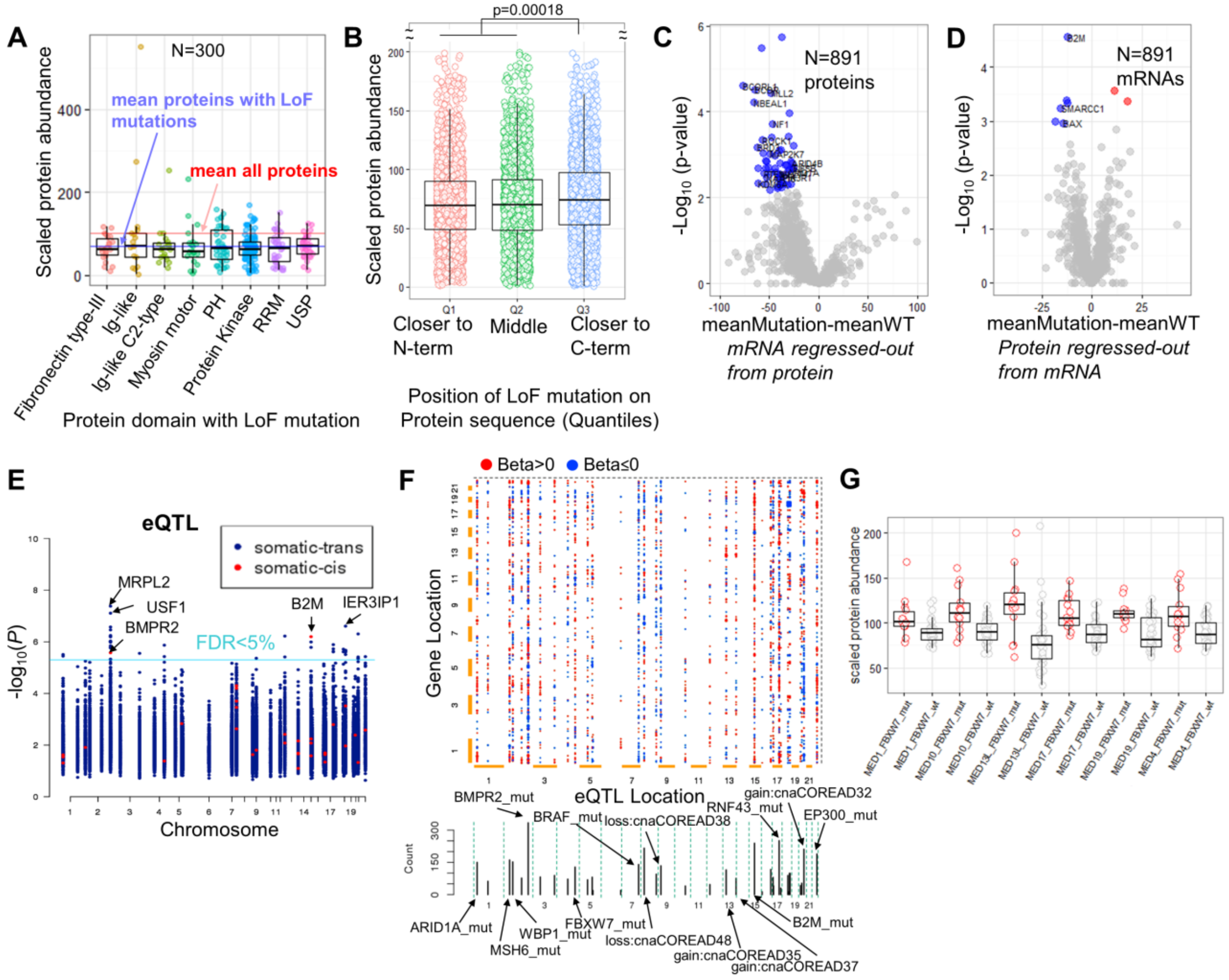


**Figure S5. The impact of mutations on protein and mRNA abundances Related to Figures 2,3,4 and 5.** A) Boxplots of scaled protein abundances for proteins with LoF mutations within specific protein domains. B) Boxplots of scaled protein abundances for proteins with LoF mutations located closer to the N-terminus, in the middle and closer to the C-terminus of the protein sequence. C) Volcano plot summarizing the effect of LoF mutations on the respective mRNA regressed-out protein levels (ANOVA test). Hits at permutation-based FDR<0.1 are coloured. D) Volcano plot summarizing the effect of LoF mutations on the respective protein regressed-out mRNA levels (ANOVA test). Hits at permutation-based FDR<0.1 are coloured. E) Identification of *cis* and *trans* eQTLs in colorectal cancer cell lines considering cancer driver variants. The p-value and genomic coordinates for the most confident non-redundant mRNA-variant association tests are depicted in the Manhattan plot. F) Representation of eQTLs as 2D plot of variants (x-axis) and associated genes (y-axis). Associations with q<0.3 are shown as dots coloured by the beta value (red: positive association, blue: negative association) while the size is increasing with the

confidence of the association. Cumulative of the number of associations per variant is plotted below the 2D matrix. G) Boxplots of protein abundance of proteins from the Mediator complex identified only by pQTL analysis, in cell lines with mutated (red) and wt (grey) *FBXW7*.

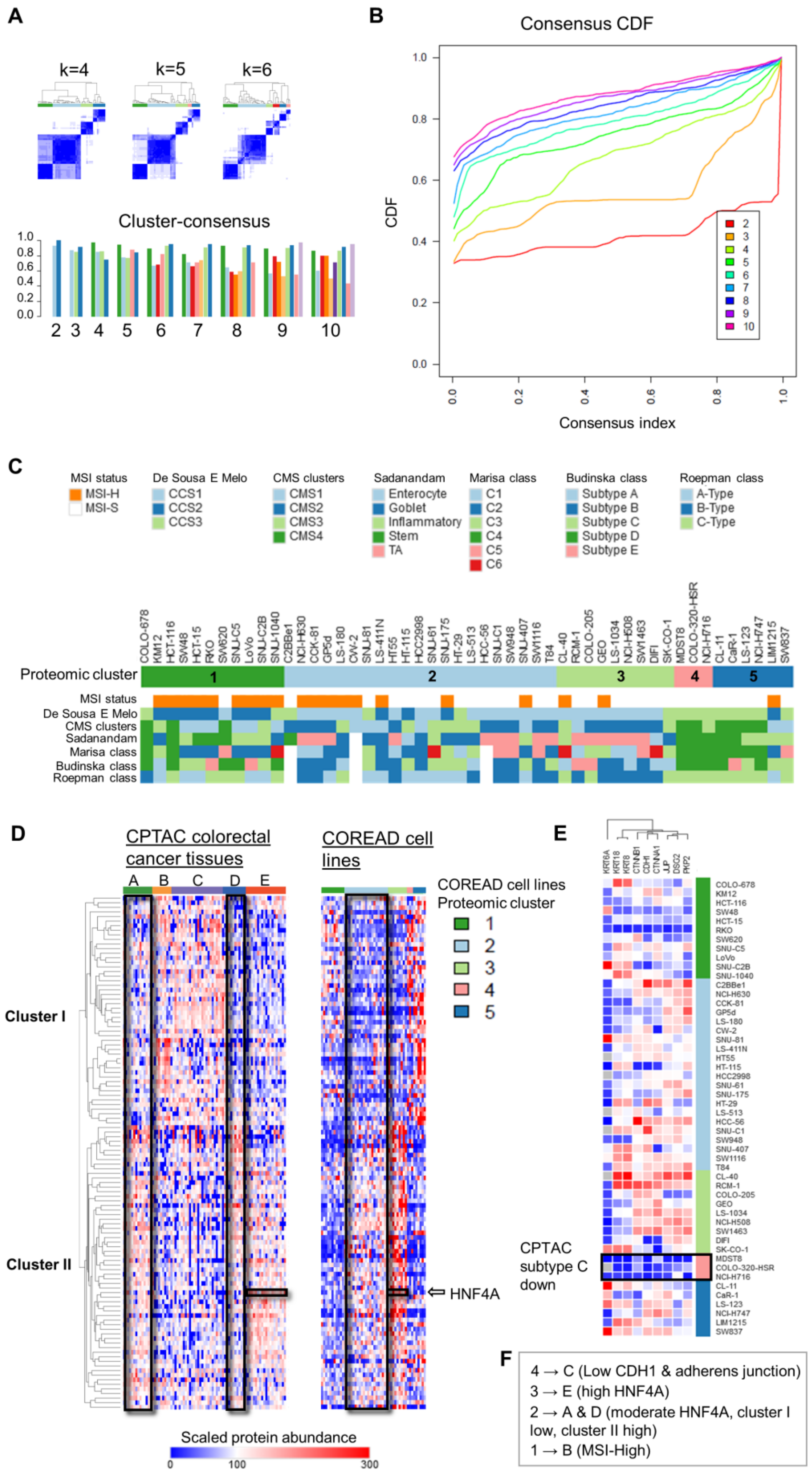
A
k=4
k=5
k=6
Cluster-consensus
1.0
0.8
0.6
0.4
0.2
0.0
2 3 4 5 6 7 8 9 10
B
Consensus CDF
CDF
Consensus index
2 3 4 5 6 7 8 9 10
C
MSI status
MSI-H
MSI-S
De Sousa E Melo
CCS1
CCS2
CCS3
CMS clusters
CMS1
CMS2
CMS3
CMS4
Sadanandam
Enterocyte
Goblet
Inflammatory
Stem
TA
Marisa class
C1
C2
C3
C4
C5
C6
Budinska class
Subtype A
Subtype B
Subtype C
Subtype D
Subtype E
Roepman class
A-Type
B-Type
C-Type
Proteomic cluster
1 2 3 4 5
MSI status
De Sousa E Melo
CMS clusters
Sadanandam
Marisa class
Budinska class
Roepman class
D
CPTAC colorectal cancer tissues
A B C D E
COREAD cell lines
COREAD cell lines Proteomic cluster
1 2 3 4 5
Cluster I
Cluster II
HNF4A
Scaled protein abundance
0 100 300
E
CPTAC subtype C down
F
4 → C (Low CDH1 & adherens junction)
3 → E (high HNF4A)
2 → A & D (moderate HNF4A, cluster I low, cluster II high)
1 → B (MSI-High)

**Figure S6. Consensus clustering of colorectal cancer cell lines and overlap with colorectal cancer tissue subtypes Related to Figure 6.** A) Proteome clusters were derived based on consensus clustering (ConsensusClusterPlus R package) using the 30% most variable proteins with no missing values. The consensus matrices for target values *k*=4,5 and 6 are visualized (top panel) along with the cluster-consensus plot displaying the mean of all pairwise consensus values between a cluster's members at each *k* (bottom panel). Balanced mean consensus values are obtained at *k*=5. B) The empirical cumulative distribution function (CDF) plot which indicates the *k* at which the distribution reaches an approximate maximum. C) Overlap of the proteomics cell line subtypes with tissue level classifications. D) Heatmaps using the cell line signature proteins that are also differentially regulated between the CPTAC colorectal cancer proteomic subtypes. Scaled protein abundances are used for both datasets. Proteins (rows) are hierarchically clustered based on the CPTAC data and columns are sorted by CPTAC proteomics subtype (left panel) or by the COREAD cell lines subtypes (right panel). E) The abundance profiling of CPTAC subtype C down-regulated proteins across the COREAD cell lines panel. Strong down-regulation is observed in COREAD cell lines subtype 4. F) Summary of the overlap between the CPTAC colorectal cancer tissue proteome subtypes and the COREAD cell lines proteomic subtypes.

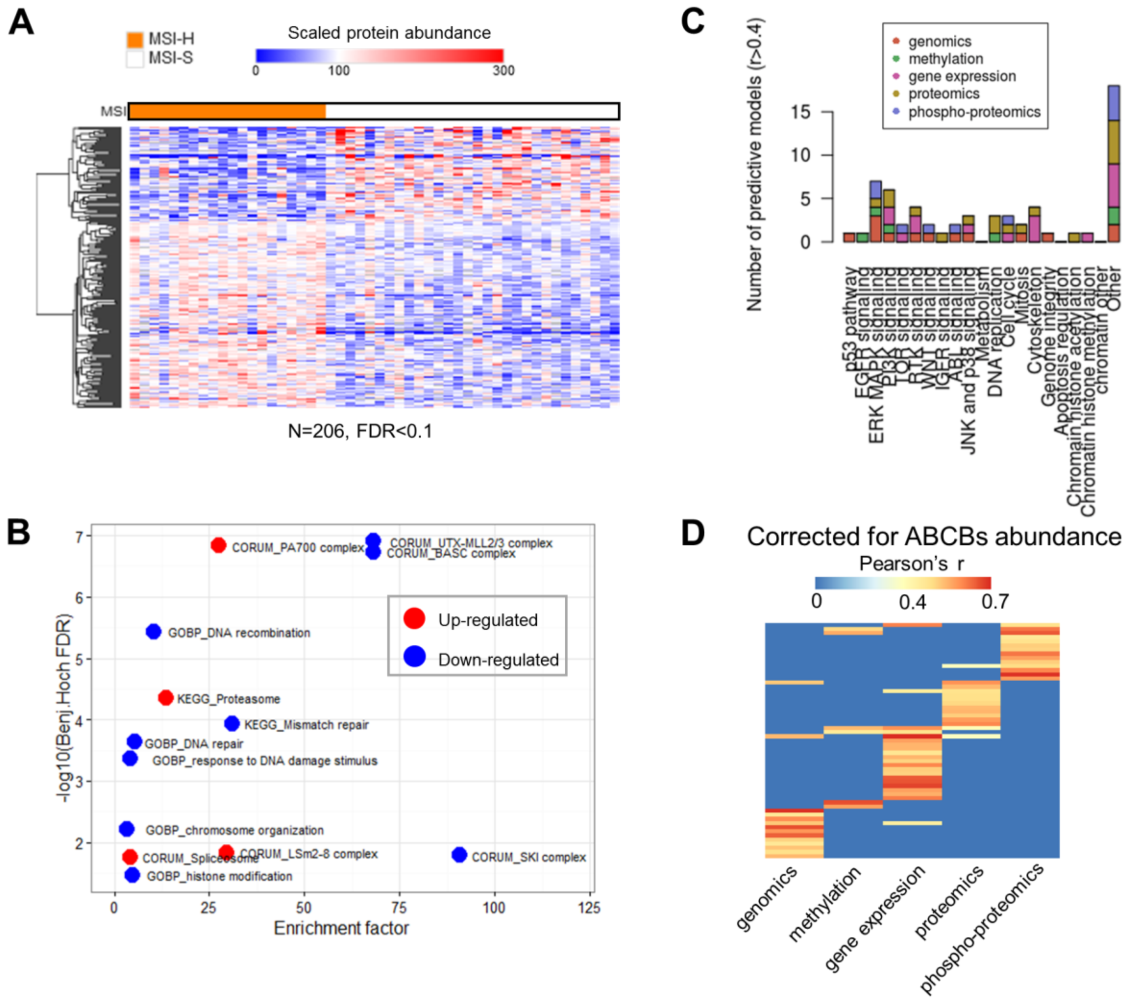


**Figure S7. Proteins associated with microsatellite instability and pharmacoproteomic models Related to Figures 6 and 7.** A) Heatmap of MSI-high associated proteins (Welch t-test, permutation-based FDR<0.1). Rows represent proteins and columns correspond to cell lines grouped according to the MSI status. B) Scatter plot of fold-enrichment versus significance FDR for enriched biological processes, KEGG pathways and CORUM complexes in the MSI-high associated proteins. C) The number of drugs where response was specifically predicted by one

molecular data type, stratified by each of the four molecular data types and by the 21 drug classes as defined by Iorio et al. (2016). D) Heatmap indicating for each drug and each data type whether a predictive model could be fitted using ABCBs-corrected drug response data.

## Supplemental tables titles and legends

**Table S1 Related to Figures 1,2,3,5,6 and 7. Relative protein abundances in the COREAD cell lines.** Scaled protein quantification values for 50 colorectal cancer cell lines. Proteins affected by mutations or DNA copy number alterations are annotated by ANOVA p-value and average log2fold-changes where applicable.

**Table S2 Related to Figures 6, S2 and S4. Relative phosphopeptide abundances in the COREAD cell lines.** Scaled phosphopeptide quantification values for 50 colorectal cancer cell lines. Phosphopeptides are annotated by the known regulatory kinase and by KEGG pathway where applicable. The values are not corrected or normalized for total protein levels.

**Table S3 Related to Figure 1. Correlation of proteins belonging to known CORUM complexes.** Overlap of known CORUM protein complexes with the COREAD cell lines proteome. The mean and median correlation between protein complex components is shown for all complexes. Proteins with outlier profiles (high or low correlations) are labelled.

**Table S4 Related to Figure 1. Annotation of WGCNA modules.** Enrichment of Gene Ontology, GSEA, KEGG, CORUM and Pfam terms for the WGCNA modules. Significance FDR is shown for each term.

**Table S5 Related to Figure 1. The full WGCNA network.** The entire WGCNA correlation network with weights greater than 0.02. The respective Pearson's correlations are also shown and CORUM interactions are highlighted (value=1). Modules that are possibly driven by DNA copy number variations are highlighted (value=1).

**Table S6 Related to Figure 4. Proteomics and RNA-seq results for the ARID1A, ARID2 and PBRM1 CRISPR-cas9 experiments.** Absolute and scaled S/N TMT values are shown for the proteomics data. ANOVA p-values refer to any comparison between the WT, ARID1A, ARID2 and PBRM1 replicate groups. RNA-seq quantification and significance is shown only for the respective identified proteins.

**Table S7 Related to Figure 7 and Figure S7. Associations between drug response and molecular data.** The predictive performance, data type and target pathway is shown for each association. The column "Corrected for ABCBs?" indicates whether the feature was predicted with the mean protein abundance of ABCB1 and ABCB11 regressed-out from drug response data.

## Supplemental Experimental Procedures

### Colorectal cancer cell lines culture and reagents

Cells were grown in either DMEM/F12 medium (Gibco) supplemented with 10% fetal calf serum (v/v) (Gibco) and 50 U/mL penicillin, 50 mg/mL streptavidin (Gibco), or RPMI 1640 medium (Gibco) supplemented with 10% fetal calf serum (v/v) (Gibco), 50 U/mL penicillin, 50 mg/mL streptavidin (Gibco), 2.5 mg/mL glucose (Sigma-Aldrich) and 1 mM sodium pyruvate (Gibco), and maintained at 37 °C in a humidified atmosphere at 5% $CO_2$. Cells were harvested by incubating with TrypLE (Gibco) until detached, and washing twice with cold PBS solution before snap freezing on dry ice.

### Viability assays

Cells were seeded at 5,000 cells per well in 96-well tissue culture plates in growth media. The following day, docetaxel (Selleckchem) was added to give a final concentration range of 0.2 nM – 50 nM, with triplicate wells of each condition. Assay was carried out in the presence or absence of 2.5 μM tariquidar (Selleckchem). Control wells received an equivalent volume of DMSO. Assay plates were incubated at 37°C, 5% $CO_2$ for 6 days, before adding

CellTiter Blue (Promega) as an indicator of cell viability. Fluorescent signal was measured after 6 h using a Molecular Devices Paradigm plate reader (560/590 nm filter).

**iPSC lines culture**

Human induced pluripotent stem cells have been maintained in mTeSR E8 media (StemCell Technologies) on Synthemax II-SC Substrate-treated plates (Corning). All lines have been passaged as single cells by incubating with Accutase for 10 min at 37 °C, scraping with cell lifters and re-suspending 5 to 10 times with a 10 mL pipette. Cells were then plated in mTeSR-E8 media containing 10 uM ROCK inhibitor (Y-27632 dihydrochloride monohydrate, StemCell Technologies). When passaging as clumps, cells were incubated 3 minutes in Gentle Dissociation Buffer, scraped with cell lifters, resuspended once or twice with a 10 mL pipette and plated in mTeSR-E8 media.

**iPSC lines CRISPR targeting**

Production of tumor suppressor gene knock out lines was achieved through the substitution of an asymmetrical exon by a Puromycin cassette and introduction of a frame-shift indel in the remaining allele. A hSpCas9 and two small guide RNA expression vectors along with a template vector were used. The template vector harboured an EF1a-Puromycin cassette with two flanking 1.5 kb homology arms designed around the asymmetric exon of interest. For each knock out line, $2\times10^6$ single cells were transfected using the Amaxa Human Stem Cell Nucleofector® Kit 2 (Lonza) with 4 ug, 3 ug and 2 ug of each plasmid, respectively, and plated in 10 cm plates. After 72 h, cells were selected in 3 ug/mL Puromycin and colonies expanded and genotyped for the presence of a frame-shift indel by Sanger sequencing. For the proteomic analysis $2\times10^5$ single cells were plated in 10 cm plates and collected once colonies showed the typical dense sharp-edged morphology. Cells were washed in DPBS, scraped with cell lifters and cell pellets were centrifuged at 2,500 rpm for 5 min at 4 °C. Pellets were then snap frozen in dry ice and stored at -80 °C.

**Protein digestion and TMT labelling**

The PBS washed cell pellets containing $2\sim3\times10^6$ cells were dissolved in 150 µL 0.1 M triethylammonium bicarbonate (TEAB), 0.1% SDS with pulsed probe sonication (EpiShear™, power 40%) on ice for 20 sec and direct boiling at 95 °C in a preheated heat block for 10 min. The sonication-boiling procedure was performed twice and cellular debris was removed by centrifugation at 12,000 rpm for 10 min. Protein concentration was measured with Quick Start Bradford Protein Assay (Bio-Rad) according to manufacturer's instructions. Aliquots containing 100 µg of total protein were prepared for trypsin digestion. Cysteine disulfide bonds were reduced with a final concentration of 5 mM tris-2-carboxymethyl phosphine (TCEP) followed by 1 h incubation in heating block at 60 °C. Cysteine residues were blocked with a final concentration of 10 mM freshly prepared Iodoacetamide (IAA) solution and 30 min incubation at room temperature in dark. Trypsin (Pierce, MS grade) was added at mass ratio 1:30 for overnight digestion. The resultant peptides were diluted up to 100 µL with 0.1 M TEAB buffer. A 41 µL volume of anhydrous acetonitrile was added to each TMT 10-plex reagent (Thermo Scientific) vial and after vortex mixing the content of each TMT vial was transferred to each sample tube. The labelling reaction was quenched after 1 hour by the addition of 8 µL 5% hydroxylamine. Samples were combined and the mixture was dried with speedvac concentrator and stored at -20 °C until the high-pH Reverse Phase (RP) fractionation.

**Peptide fractionation**

High pH Reverse Phase (RP) peptide fractionation was performed with the Waters, XBridge C18 column (2.1 x 150 mm, 3.5 µm, 120 Å) on a Dionex Ultimate 3000 HPLC system equipped with autosampler. Mobile phase (A) was composed of 0.1% ammonium hydroxide and mobile phase (B) was composed of 100% acetonitrile, 0.1% ammonium hydroxide. The TMT labelled peptide mixture was reconstituted in 100 µL mobile phase (A), centrifuged and injected for fractionation. The multi-step gradient elution method at 0.2 mL/min was as follows: for 5 minutes isocratic at 5% (B), for 35 min gradient to 35% (B), for 5 min gradient to 80% (B), isocratic for 5 minutes and re-equilibration to 5% (B). Signal was recorded at 215 and 280 nm and fractions were collected in a time dependent manner every 30 sec. The collected fractions were dried with SpeedVac concentrator and stored at -20 °C until the LC-MS analysis. For the replication sample set (7th set) and the CRISPR/cas9 proteomic experiments, peptide fractionation was performed on reversed-phase OASIS HLB cartridges at high pH and up to 12 fractions were collected for each set.

**Phosphopeptide enrichment**

The peptide fractions were reconstituted in 10 uL of 20% isopropanol, 0.5% formic acid binding solution and were loaded on 10 uL of phosphopeptide enrichment IMAC resin (PHOS-Select™ Iron Affinity Gel) already washed and

conditioned with binding solution in custom made filter tips fitted on the eppendorf tubes caps. The resin was washed three times with 40 uL of binding solution and centrifugation at 300 g after 2 h of binding and the flow-through solutions were collected. Phosphopeptides were eluted three times with 70 uL of 40% acetonitrile, 400 mM ammonium hydroxide solution. Both the eluents and flow-through solutions were dried in a speedvac and stored at -20 °C until the phosphoproteomic and proteomic LC-MS analysis respectively.

**LC-MS analysis**

LC-MS analysis was performed on the Dionex Ultimate 3000 UHPLC system coupled with the Orbitrap Fusion Tribrid Mass Spectrometer (Thermo Scientific). Each peptide fraction was reconstituted in 40 μL 0.1% formic acid and a volume of 7 μL was loaded to the Acclaim PepMap 100, 100 μm × 2 cm C18, 5 μm, 100 Ȧ trapping column with the μlPickUp mode at 10 μL/min flow rate. The sample was then subjected to a multi-step gradient elution on the Acclaim PepMap RSLC (75 μm × 50 cm, 2 μm, 100 Å) C18 capillary column retrofitted to an electrospray emitter (New Objective, FS360-20-10-D-20) at 45 °C. Mobile phase (A) was composed of 0.1% formic acid and mobile phase (B) was composed of 80% acetonitrile, 0.1% formic acid. The gradient separation method at flow rate 300 nL/min was as follows: for 95 min gradient to 42% B, for 5 min up to 95% B, for 8 min isocratic at 95% B, re-equilibration to 5% B in 2 min, for 10 min isocratic at 5% B.
Precursors were selected with mass resolution of 120k, AGC $3\times10^5$ and IT 100 ms in the top speed mode within 3 sec and were isolated for CID fragmentation with quadrupole isolation width 0.7 Th. Collision energy was set at 35% with AGC $1\times10^4$ and IT 35 ms. MS3 quantification spectra were acquired with further HCD fragmentation of the top 10 most abundant CID fragments isolated with Synchronous Precursor Selection (SPS) excluding neutral losses of maximum m/z 30. Iontrap isolation width was set at 0.5 Th, collision energy was applied at 45% and the AGC setting was at $6\times10^4$ with 100 ms IT. The HCD MS3 spectra were acquired within 120-140 m/z with 60k resolution. Targeted precursors were dynamically excluded for further isolation and activation for 45 seconds with 7 ppm mass tolerance. Phosphopeptide samples were analyzed with a CID-HCD method at the MS2 level. MS level AGC was set at $5\times10^5$, IT was set at 150 ms and exclusion duration at 30sec. AGC settings for CID and HCD fragmentation were $5\times10^4$ and $2\times10^5$ respectively. The fractions for the replication and CRISPR/cas9 sets were analysed with 180 min and 300 min LC-MS runs respectively and the analysis was repeated by setting upper intensity thresholds between 2-$5\times10^6$ to capture lower abundant peptides. The total data collection was accomplished with 469 LC-MS runs in about 1,000 hours of analysis.

**Protein identification and quantification**

The acquired mass spectra were submitted to SequestHT search in Proteome Discoverer 1.4 (P.D 2.1 for the CRISPR-cas9 experiments) for protein identification and quantification. The precursor mass tolerance was set at 20 ppm and the fragment ion mass tolerance was set at 0.5 Da for the CID and at 0.02 Da for the HCD spectra used for the phosphopeptide analysis. Spectra were searched for fully tryptic peptides with maximum 2 miss-cleavages and minimum length of 6 amino acids. TMT6plex at N-termimus, K and Carbamidomethyl at C were defined as static modifications. Dynamic modifications included oxidation of M and Deamidation of N,Q. Maximum two different dynamic modifications were allowed for each peptide with maximum two repetitions each. Search for phospho-S,T,Y was included only for the IMAC data. Peptide confidence was estimated with the Percolator node. Peptide FDR was set at 0.01 and validation was based on q-value and decoy database search. All spectra were searched against a UniProt fasta file containing 20,165 reviewed human entries. The Reporter Ion Quantifier node included a custom TMT-10plex Quantification Method with integration window tolerance 15 ppm, integration method the Most Confident Centroid at the MS3 level (or at the MS2 level for the IMAC data) and missing channels were replaced by minimum intensity. Only peptides uniquely belonging to protein groups were used for quantification. Peptide-spectrum matches (PSMs) with mean TMT intensity less than 500 across samples were discarded. The TMT intensities of PSMs uniquely matching to the same protein or phosphopeptide were summed to obtain protein and phosphopeptide level intensities respectively. Protein and phosphopeptide summed intensities were further corrected for equal loading across samples by median normalization (divide by column median). Scaled quantitative values in the range of 0 to 1,000 were obtained by dividing each TMT value with the mean TMT intensity across samples per protein in each multiplex set separately (divide by row mean × 100, per sample set). To detect net phosphorylation changes, the relative protein abundances were regressed out from the respective phosphopeptide levels. Phosphorylation abundances were set as the dependent variables (y) and protein abundances were set as the independent variables (x). The residuals of the y~x linear model were the phosphorylation levels not driven by protein abundance levels.

**RNAseq data processing and identification of differentially expressed genes for the CRISPR/cas9 cell lines**
Knockout human induced pluripotent stem cells (hiPSC) for *ARID1A*, *ARID2* and *PBRM1* and WT cells were cultured in the same conditions as specified in the protein extraction section. RNA was extracted using the RNAeasy Kit (QiAGEN) from a total of six biological replicates per gene knockout and four WT cells for a total of 22 samples. Independent barcoded stranded libraries were prepared for each sample and pooled into a multiplex library that was sequenced across six lanes using the Illumina HiSeq4000. Raw 75bp paired-end reads were aligned to the human reference genome (GRCh38) using STAR (v2.5.0a) (Dobin et al., 2013) and ENSEMBL (v84) human annotation. Subsequently, uniquely mapped read pairs with a mapping quality >10 were counted using htseq-count (Anders et al., 2015) with the model intersection_nonempty and ENSEMBL v84 annotation. Raw counts were normalised by calculating the transcripts per kilobase per million (TPM) obtained for each gene for each biological replicate. Pearson correlation among the different samples was calculated based on the TPM values of all the protein coding genes. Differentially expressed genes were identified by performing paired comparisons between the three independent groups of targeted hiPS cells (*ARID1A, ARID2 and PBRM1)* against WT hiPS cells (BoB) using DESeq2 (Love et al., 2014) (v1.10.1). Once dispersion estimates and normalised counts were calculated, genes with mean normalized <1, across all samples within the comparison, were filtered out. P-values were re-adjusted using the Benjamini-Hochberg correction for multiple-testing.

**Visualization on genome coordinates**
The peptides identified in this study were mapped onto the human reference genome GRCh38/hg38 using GENCODE v25 and GRCh37/hg19 using GENCODE v25lift37 (Wright et al., 2016) through the peptide to genome mapping tool PoGo (http://www.sanger.ac.uk/science/tools/pogo). The resulting BED output files were used to create a track-hub (Raney et al., 2014), i.e. web-accessible directory of genomic related data for visualisation of a large number of genome-wide data sets, through application of the TrackHubGenerator tool (http://www.sanger.ac.uk/science/tools/trackhubgenerator). The track-hub is made available through ftp://ngs.sanger.ac.uk/production/proteogenomics/WTSI_proteomics_COREAD and can be loaded in prominent online genome browsers. The Hub can be loaded in the Ensembl genome browser (Aken et al., 2017) through selection of 'Custom tracks' and copying the following URL into the 'Data' field and selection of 'Track Hub' as data format: http://ngs.sanger.ac.uk/production/proteogenomics/WTSI_proteomics_COREAD/hub.txt. After adding the data and closing the configuration panel five new tracks will appear in the "Region in detail" view showing the overall identified peptides, and phosphopeptides. The tracks can also be loaded in the UCSC genome browser (Kent et al., 2002) through 'My Data' and the 'My Hubs'. After copying the above link and adding the hub the tracks will appear in the browser after selection of the reference assembly hg19 or hg38.
To support other types of visualisation the additional folder 'suppl' in the track-hub directory provides downloadable GTF mappings of the peptides with associated gene names and gene biotypes for reference assemblies hg19 and hg38. Furthermore, comparative visualisation of peptide quantitation across all 50 COREAD cell lines for both reference assemblies are provided through GCT files in the respective assembly folders. These files can be visualised in the Integrative Genomics Viewer (IGV) (Robinson et al., 2011). After selection of the genome assembly the respective files can be loaded through the file selection dialog. Comparative visualisation on protein level in association with chromosome bands is provided separately in the file 'WTSI_proteomics_COREAD_50_proteome_bands.gct'.

**COREAD gene expression data**
The mRNA relative abundances between 45 colorectal cancer cell lines were computed from publicly available microarray gene expression data (ArrayExpress, accession: E-MTAB-3610) using robust Multi-Array Average (RMA) (Bolstad et al., 2003). Scaled values were obtained by row-mean normalization as applied to the proteomics data.

**Weighted correlation network analysis**
Weighted correlation network analysis was performed with the WGCNA package in RStudio using 8,295 proteins quantified in at least 80% of the cell lines. A soft threshold at power 7 was selected based on scale free topology model fit. Other parameters included: mergeCutHeight = 0.25 and minModuleSize = 3. Gene Ontology annotation of the modules was performed by the WGCNA package and enrichment for additional terms was performed with Fisher's test using GSEA, GOBP-slim, CORUM, KEGG, and Pfam terms in Perseus software (Tyanova et al., 2016).

**Consensus clustering of the cell lines**

Unsupervised clustering of the cell lines was performed with the ConsensusClusterPlus R package using the top 30% most variable proteins without missing values (N=2,161). Proteome clusters were derived based on k-means clustering and 1,000 resampling repetitions in the range of 2 to 10 clusters. The consensus matrices for target values $k$=4, 5 and 6 were visualized along with the empirical cumulative distribution function (CDF) plot which indicates the $k$ at which the distribution reaches an approximate maximum and the cluster-consensus plot displaying the mean of all pairwise consensus values between a cluster's members at each $k$.

**Comparison of COREAD cell lines proteomic subtypes with CPTAC colorectal cancer proteomic subtypes**

To assess the overlap between the COREAD and the CPTAC colorectal cancer proteomic subtypes from Zhang et al., we tested whether our COREAD signature proteins (differentially expressed between the five subtypes, ANOVA test, permutation-based FDR<0.05, N=723) were also differentially expressed between the CPTAC subtypes. For the CPTAC data, proteins with mean intensity greater than 1.4 (median label free intensity) across tissues were considered (N=3,615), and the label free quantification values were row-mean scaled similarly to the cell line data. About 75% of the COREAD signature proteins (N=251) that were found in the scaled CPTAC subset were also differentially regulated between the CPTAC subtypes (ANOVA test, permutation-based FDR<0.1). The most variable proteins of the latter subset were visualized with hierarchical clustering and the resultant clusters were compared with the COREAD subtypes profiles.

**Statistical tests and visualization**

ROC curves were plotted in Python (v 2.7.10) module Seaborn (v 0.7.1) using known STRING and CORUM interactions as true positive hits. Enrichment for biological terms and pathways was performed in Perseus 1.4 software (Tyanova et al., 2016) with Fisher's test or with the 1D-annotation enrichment method (Cox and Mann, 2012). The enrichment score indicates whether the proteins in a given pathway tend to be systematically up-regulated or down-regulated based on Wilcoxon-Mann-Whitney test. The 1D-annotation enrichment method was also applied for the enrichment of KEGG pathways with low and high mRNA-to-protein correlations and for kinase enrichment analysis using known kinase-substrate associations from the PhosphoSitePlus database and non-regressed phosphorylation abundances. All terms were filtered for Benjamini-Hochberg FDR<0.05. Correlation analysis of regressed phosphorylation profiles was performed in RStudio with Benjamini-Hochberg FDR multiple testing corrections. PHOSIDA was used for enrichment of phosphorylation motifs (Gnad et al., 2011). ANOVA and Welch's tests were performed in Perseus 1.4 software. Permutation based FDR correction was applied to the ANOVA test p-values for the assessment of the impact of mutations and copy number variations on protein and mRNA abundances in Perseus 1.4 software. The web-based tool Morpheus (https://software.broadinstitute.org/morpheus/) was used for hierarchical clustering and visualization of heatmaps. Volcano plots, boxplots, distribution plots, scatter plots and bar plots were drawn in RStudio with the ggplot2 and ggrepel packages. KEGG pathway enrichment for the identified phosphoproteins was performed in DAVID (Huang et al., 2009) (https://david.ncifcrf.gov/). PROSITE protein domains (Sigrist et al., 2010) were used to assess the impact of mutated domains on protein abundances. Transcription factor enrichment analysis was performed in the Enrichr tool (http://amp.pharm.mssm.edu/Enrichr/). STRING and Cytoscape 3.2.1 (Shannon et al., 2003) were used for network analysis and visualization.

**Identification of pQTL and eQTL**

We performed a whole-genome protein level QTL (pQTL) mapping by testing for associations between the protein level measurements and a set of 19 variant genes and 17 CNAs representing colorectal cancer drivers, filtered for at least 5 events across the cell lines. From the original 9,489 proteins measured, we selected proteins expressed across all cell lines and aligned uniquely to one Ensemble gene (N=6,929). Proteins on X, Y and mitochondrial chromosome were excluded. To increase the robustness with respect to possible outlying protein values, all protein measures were quantile normalized to a Gaussian distribution prior to fitting a model. All associations were implemented by LIMIX using a linear regression test. Let the protein level measurements be $\boldsymbol{y}$, $\boldsymbol{x}_s$ corresponds to the binary representation of somatic variants. We can write the linear regression model as:

$$\boldsymbol{y} = \boldsymbol{1}\mu + \boldsymbol{x}_s\beta_s + \boldsymbol{\psi}, \text{ where } \boldsymbol{\psi} \sim N(0, \sigma_e^2\boldsymbol{I}).$$

Here $\beta_s$ denotes the effect size of the tested variant, $\mu$ is the intercept and $\boldsymbol{\psi}$ is the residual noise.Similarly for eQTL analysis, genes on X, Y and mitochondrial chromosome were excluded (N=16,546). We used a shared normalization, regression and multiple testing correction for eQTL as pQTL.

**Drug response prediction**

For the associations with drug response, we used the genomics, methylation, gene expression, proteomics, phosphoproteomics (using only phosphorylation sites of kinases/phosphatases with total protein abundance regressed-out, N=436) and drug response data from the COREAD cell lines in the GDSC1000. This encompassed the mutation status of 38 colorectal cancer genes, gains or losses in 48 copy number regions, methylation status of CpG islands in 32 gene promoters, gene expression and proteomics. For a fair comparison between data types (genomics, methylation, gene expression and proteomics), the same cross-validation folds were used for the different data types, and hence we only considered the 45 cell lines for which data was available in all data types. Given the limited number of available COREAD cell lines, we also limited the number of features. For the genomics and methylation we only used the Cancer Functional Events (CFEs), as defined by Iorio et al. (2016). For the proteomics, we used a subset of 2,161 most-variable proteins (the top 30% proteins, ranked by standard deviation) for which data was available in all cell lines. Similarly, for a fair comparison to the proteomics data, we considered the 2,161 most-variable transcripts. Finally, we used k-nearest neighbours (with k=10) to impute missing values in the phosphoproteomics data.

For each drug and each data type an Elastic Net model (Zou and Hastie, 2005) was fitted to predict the drug response (log IC50), using the implementation from the R package "glmnet" version 1.4 (2009). The hyper-parameter $\lambda$ was optimized using 10-fold cross-validation and $\alpha$ was set to 0.5. Predictive performance was determined using Pearson correlation between the observed and the predicted IC50s, using the predictions from the cross-validation. When the difference in predictive performance between the optimal model and a model fitting only, an intercept was less than one standard deviation (i.e. when a model using λ1SE selects zero features), the predictive performance was set to zero. The ABCBs-corrected drug response data were the residuals of the linear regression model where the mean abundance of ABCB1/ABCB11 was used as the independent variable (x) and the log2(row mean-scaled) drug response data were used as the dependent variables (y).

**Supplemental References**


Aken, B.L., Achuthan, P., Akanni, W., Amode, M.R., Bernsdorff, F., Bhai, J., Billis, K., Carvalho-Silva, D., Cummins, C., Clapham, P.*, et al.* (2017). Ensembl 2017. Nucleic acids research *45*, D635-D642.

Anders, S., Pyl, P.T., and Huber, W. (2015). HTSeq-a Python framework to work with high-throughput sequencing data. Bioinformatics *31*, 166-169.

Bolstad, B.M., Irizarry, R.A., Astrand, M., and Speed, T.P. (2003). A comparison of normalization methods for high density oligonucleotide array data based on variance and bias. Bioinformatics *19*, 185-193.

Cox, J., and Mann, M. (2012). 1D and 2D annotation enrichment: a statistical method integrating quantitative proteomics with complementary high-throughput data. BMC bioinformatics *13 Suppl 16*, S12.

Dobin, A., Davis, C.A., Schlesinger, F., Drenkow, J., Zaleski, C., Jha, S., Batut, P., Chaisson, M., and Gingeras, T.R. (2013). STAR: ultrafast universal RNA-seq aligner. Bioinformatics *29*, 15-21.

Gnad, F., Gunawardena, J., and Mann, M. (2011). PHOSIDA 2011: the posttranslational modification database. Nucleic acids research *39*, D253-260.

Huang, D.W., Sherman, B.T., and Lempicki, R.A. (2009). Systematic and integrative analysis of large gene lists using DAVID bioinformatics resources. Nature protocols *4*, 44-57.

Kent, W.J., Sugnet, C.W., Furey, T.S., Roskin, K.M., Pringle, T.H., Zahler, A.M., and Haussler, D. (2002). The human genome browser at UCSC. Genome research *12*, 996-1006.

Love, M.I., Huber, W., and Anders, S. (2014). Moderated estimation of fold change and dispersion for RNA-seq data with DESeq2. Genome biology *15*, 550.

Raney, B.J., Dreszer, T.R., Barber, G.P., Clawson, H., Fujita, P.A., Wang, T., Nguyen, N., Paten, B., Zweig, A.S., Karolchik, D.*, et al.* (2014). Track data hubs enable visualization of user-defined genome-wide annotations on the UCSC Genome Browser. Bioinformatics *30*, 1003-1005.

Robinson, J.T., Thorvaldsdottir, H., Winckler, W., Guttman, M., Lander, E.S., Getz, G., and Mesirov, J.P. (2011). Integrative genomics viewer. Nature biotechnology *29*, 24-26.
Shannon, P., Markiel, A., Ozier, O., Baliga, N.S., Wang, J.T., Ramage, D., Amin, N., Schwikowski, B., and Ideker, T. (2003). Cytoscape: a software environment for integrated models of biomolecular interaction networks. Genome research *13*, 2498-2504.
Sigrist, C.J., Cerutti, L., de Castro, E., Langendijk-Genevaux, P.S., Bulliard, V., Bairoch, A., and Hulo, N. (2010). PROSITE, a protein domain database for functional characterization and annotation. Nucleic acids research *38*, D161-166.
Tyanova, S., Temu, T., Sinitcyn, P., Carlson, A., Hein, M.Y., Geiger, T., Mann, M., and Cox, J. (2016). The Perseus computational platform for comprehensive analysis of (prote)omics data. Nature methods *13*, 731-740.
Wright, J.C., Mudge, J., Weisser, H., Barzine, M.P., Gonzalez, J.M., Brazma, A., Choudhary, J.S., and Harrow, J. (2016). Improving GENCODE reference gene annotation using a high-stringency proteogenomics workflow. Nature communications *7*.
Zou, H., and Hastie, T. (2005). Regularization and variable selection via the elastic net. J Roy Stat Soc B *67*, 301-320.
