# Supplementary material for: Genomic Determinants of Protein Abundance Variation in Colorectal Cancer Cells
Source: Cell Rep. 2017 Aug 29;20(9):2201–14. doi: 10.1016/j.celrep.2017.08.010 (PMC5583477; doi:10.1016/j.celrep.2017.08.010)
Supplement: Document S2. Article plus Supplemental Information [file mmc9.pdf]

Cell Reports

Resource

Genomic Determinants of Protein Abundance Variation in Colorectal Cancer Cells

Graphical Abstract

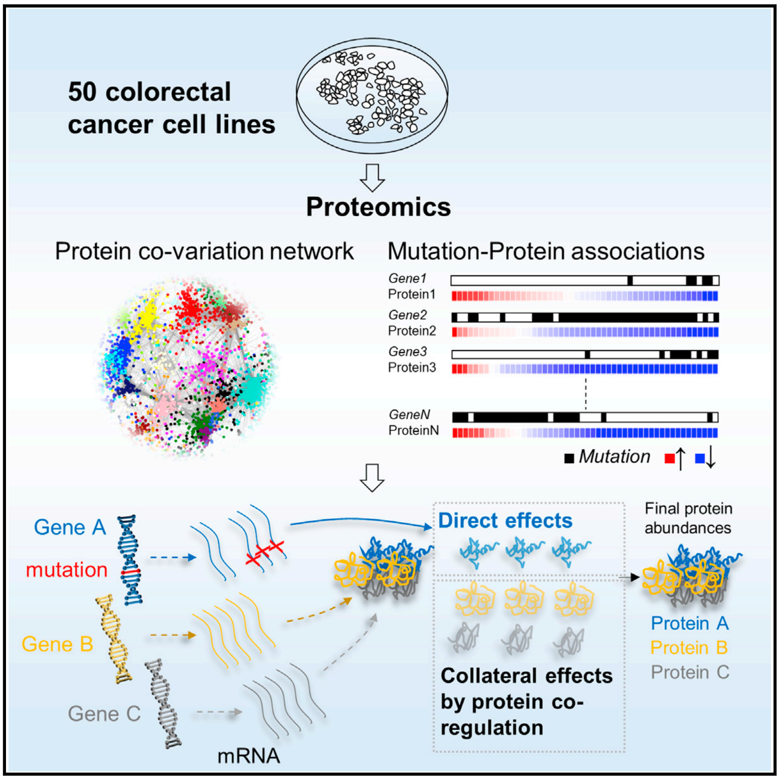

Authors

Theodoros I. Roumeliotis, Steven P. Williams, Emanuel Gonçalves, ..., Julio Saez-Rodriguez, Ultan McDermott, Jyoti S. Choudhary

Correspondence

tr6@sanger.ac.uk (T.I.R.), jc4@sanger.ac.uk (J.S.C.)

In Brief

Roumeliotis et al. use in-depth proteomics to assess the impact of genomic alterations on protein networks in colorectal cancer cell lines. Cell-line-specific network signatures are inferred de novo by protein quantification profiles and ultimately expose the collateral and transcript-independent effects of detrimental mutations on protein complexes.

Highlights

- The cancer cell "co-variome" recapitulates functional protein associations
- Loss-of-function mutations can impact protein levels beyond mRNA regulation
- The consequences of genomic alterations can propagate through protein interactions
- We provide insight into the molecular organization of colorectal cancer cells

Accession Numbers

PXD005235

Roumeliotis et al., 2017, Cell Reports *20*, 2201–2214
August 29, 2017 © 2017 The Author(s).
http://dx.doi.org/10.1016/j.celrep.2017.08.010

Cell Reports
Resource

OPEN ACCESS
CellPress

Genomic Determinants of Protein Abundance Variation in Colorectal Cancer Cells

Theodoros I. Roumeliotis,[1,9,10,*] Steven P. Williams,[1,9] Emanuel Gonçalves,[2] Clara Alsinet,[1] Martin Del Castillo Velasco-Herrera,[1] Nanne Aben,[4] Fatemeh Zamanzad Ghavidel,[2] Magali Michaut,[4] Michael Schubert,[2] Stacey Price,[1] James C. Wright,[1] Lu Yu,[1] Mi Yang,[3] Rodrigo Dienstmann,[6,7] Justin Guinney,[6] Pedro Beltrao,[2] Alvis Brazma,[2] Mercedes Pardo,[1] Oliver Stegle,[2] David J. Adams,[1,10] Lodewyk Wessels,[4,5,10] Julio Saez-Rodriguez,[2,3,10] Ultan McDermott,[1,10] and Jyoti S. Choudhary[1,8,10,11,*]

[1]Wellcome Trust Sanger Institute, Wellcome Genome Campus, Cambridge CB10 1SA, UK
[2]European Molecular Biology Laboratory, European Bioinformatics Institute (EMBL-EBI), Wellcome Genome Campus, Cambridge CB10 1SD, UK
[3]Faculty of Medicine, Joint Research Center for Computational Biomedicine, RWTH Aachen University, Aachen 52057, Germany
[4]Division of Molecular Carcinogenesis, Computational Cancer Biology, the Netherlands Cancer Institute, Amsterdam 1066, the Netherlands
[5]Faculty of EEMCS, Delft University of Technology, Delft 2628, the Netherlands
[6]Computational Oncology, Sage Bionetworks, Fred Hutchinson Cancer Research Center, Seattle, WA 98109-1024, USA
[7]Oncology Data Science Group, Vall d'Hebron Institute of Oncology, Barcelona 08035, Spain
[8]Functional Proteomics Group, Chester Beatty Laboratories, The Institute of Cancer Research, London SW3 6JB, UK
[9]These authors contributed equally
[10]Senior author
[11]Lead Contact
*Correspondence: tr6@sanger.ac.uk (T.I.R.), jc4@sanger.ac.uk (J.S.C.)
http://dx.doi.org/10.1016/j.celrep.2017.08.010

SUMMARY

Assessing the impact of genomic alterations on protein networks is fundamental in identifying the mechanisms that shape cancer heterogeneity. We have used isobaric labeling to characterize the proteomic landscapes of 50 colorectal cancer cell lines and to decipher the functional consequences of somatic genomic variants. The robust quantification of over 9,000 proteins and 11,000 phosphopeptides on average enabled the de novo construction of a functional protein correlation network, which ultimately exposed the collateral effects of mutations on protein complexes. CRISPR-cas9 deletion of key chromatin modifiers confirmed that the consequences of genomic alterations can propagate through protein interactions in a transcript-independent manner. Lastly, we leveraged the quantified proteome to perform unsupervised classification of the cell lines and to build predictive models of drug response in colorectal cancer. Overall, we provide a deep integrative view of the functional network and the molecular structure underlying the heterogeneity of colorectal cancer cells.

INTRODUCTION

Tumors exhibit a high degree of molecular and cellular heterogeneity due to the impact of genomic aberrations on protein networks underlying physiological cellular activities. Modern mass-spectrometry-based proteomic technologies have the capacity to perform highly reliable analytical measurements of proteins in large numbers of subjects and analytes, providing a powerful tool for the discovery of regulatory associations between genomic features, gene expression patterns, protein networks, and phenotypic traits (Mertins et al., 2016; Zhang et al., 2014, 2016). However, understanding how genomic variation affects protein networks and leads to variable proteomic landscapes and distinct cellular phenotypes remains challenging due to the enormous diversity in the biological characteristics of proteins. Studying protein co-variation holds the promise to overcome the challenges associated with the complexity of proteomic landscapes as it enables grouping of multiple proteins into functionally coherent groups and is now gaining ground in the study of protein associations (Stefely et al., 2016; Wang et al., 2017). Colorectal cancer cell lines are widely used as cancer models; however, their protein and phosphoprotein co-variation networks and the genomic factors underlying their regulation remain largely unexplored.

Here, we studied a panel of 50 colorectal cancer cell lines (colorectal adenocarcinoma [COREAD]) using isobaric labeling and tribrid mass spectrometry proteomic analysis in order to assess the impact of somatic genomic variants on protein networks. This panel has been extensively characterized by whole-exome sequencing, gene expression profiling, copy number and methylation profiling, and the frequency of molecular alterations is similar to that seen in clinical colorectal cohorts (Iorio et al., 2016). First, we leveraged the robust quantification of over 9,000 proteins to build de novo protein co-variation networks, and we show that they are highly representative of known protein complexes and interactions. Second, we rationalize the impact of genomic variation in the context of the cancer cell protein co-variation network (henceforth, "co-variome") to uncover protein network vulnerabilities. Proteomic and RNA sequencing (RNA-seq) analysis of human induced pluripotent stem cells (iPSCs) engineered with gene knockouts of key

This is an open access article under the CC BY license (http://creativecommons.org/licenses/by/4.0/).

chromatin modifiers confirmed that genomic variation can be transmitted from directly affected proteins to tightly co-regulated distant gene products through protein interactions. Overall, our results constitute an in-depth view of the molecular organization of colorectal cancer cells widely used in cancer research.

RESULTS

Quantified Proteome and Phosphoproteome Coverage and Correlation with Gene Expression

To assess the variation in protein abundance and phosphorylation within a panel of 50 colorectal cancer cell lines (COREAD), we utilized isobaric peptide labeling (TMT-10plex) and MS3 quantification (Figure S1A). We obtained relative quantification between the different cell lines (scaled intensities range: 0–1,000) for an average of 9,410 proteins and 11,647 phosphopeptides (Tables S1 and S2; Figure S1B). To assess the reproducibility of our data, we computed the coefficient of variation (CV) (CV = SD/mean) of protein abundances for 11 cell lines measured as biological replicates. The median CV in our study was 10.5%, showing equivalent levels of intra-laboratory biological variation with previously published TMT data for seven colorectal cancer cell lines (McAlister et al., 2014; Figure S1C). Inter-laboratory comparison for the 7 cell lines common in both studies showed median CV = 13.9% (Figure S1C). The additional variation encompasses differences in sample preparation methods (e.g., digestion enzymes), mass spectrometry instrumentation, and raw signal processing. The same SW48 protein digest aliquoted in two parts and labeled with two different TMT labels within the same 10plex experiment displayed a median CV = 1.9% (Figure S1C), indicating that the labeling procedure and the mass spectrometry (MS) signal acquisition noise have very small contribution to the total variation. The protein abundance profiles for 11 cell lines measured as biological replicates in two separate sets are shown as a heatmap in Figure S1D, revealing the high heterogeneity of the COREAD proteomic landscapes. The variation between different cell lines was on average 3 times higher than the variation between replicates (Figure S1E), with 93% of the proteins exhibiting an inter-sample variation greater than the respective baseline variation between replicates. For proteins participating in basic cellular processes (selected Kyoto encyclopedia of genes and genomes [KEGG] pathways), the median CV between biological replicates was as low as 8% (Figure S1F). At the phosphopeptide level, the SW48 biological replicates across all multiplex sets displayed a median CV = 22% (Figure S1G), reflecting the generally higher uncertainty of the peptide level measurements compared to the protein level measurements. Taken together, our results show that protein abundance differences as low as 50% or 1.5-fold (>2 × CV%) can be reliably detected using our proteomics approach at both the proteome and phosphoproteome level.

Qualitatively, phosphorylated proteins (n = 3,565) were highly enriched for spliceosomal and cell cycle functions and covered a wide range of cancer-related pathways (Figure S2A). The phosphosites were comprised of 86% serine, 13% threonine, and <1% tyrosine phosphorylation (Figure S2B), and the most frequent motifs identified were pS-P (47% of all pS) and pT-P (63% of all pT) (Figure S2C). Approximately 70% of the quantified phosphorylation sites are cataloged in Uniprot, and 751 of these represent known kinase substrates in the PhosphoSitePlus database (Hornbeck et al., 2015). In terms of phosphorylation quantification, we observed that phosphorylation profiles were strongly correlated with the respective protein abundances (Figure S2D), and therefore, to detect net phosphorylation changes, we corrected the phosphorylation levels for total protein changes by linear regression.

Correlation analysis between mRNA (publicly available microarray data) and relative protein abundances for each gene across the cell lines indicated a pathway-dependent concordance of protein/mRNA expression with median Pearson's r = 0.52 (Figure S2E). Highly variable mRNAs tend to correspond to highly variable proteins (Spearman's r = 0.62), although with a wide distribution (Figure S2F). Notably, several genes, including *TP53*, displayed high variation at the protein level despite the low variation at the mRNA level, implicating significant post-transcriptional modulation of their abundance.

Our COREAD proteomics and phosphoproteomics data can be downloaded from ftp://ngs.sanger.ac.uk/production/proteogenomics/WTSI_proteomics_COREAD/ in annotated *.gct, *.gtf, and *.bb file formats compatible with the Integrative Genomics Viewer (Robinson et al., 2011), the Morpheus clustering web tool (https://software.broadinstitute.org/morpheus/), or the Ensembl (Aken et al., 2017) and University of California Santa Cruz (UCSC) (Kent et al., 2002) genome browsers. Our proteomics data can also be viewed through the Expression Atlas database (Petryszak et al., 2016).

The Subunits of Protein Complexes Tightly Maintain Their Total Abundance Ratios Post-transcriptionally

The protein abundance measurements allowed us to study the extent to which proteins tend to be co-regulated in abundance across the colorectal cancer cell lines. We first computed the Pearson's correlation coefficients between proteins with known physical interactions in protein complexes cataloged in the CORUM database (Ruepp et al., 2010). We found that the distribution of correlations between CORUM protein pairs was bimodal and clearly shifted to positive values (Wilcoxon test; p value $< 2.2e-16$) with mean 0.33 (Figure 1A, left panel), whereas all pairwise protein-to-protein correlations displayed a normal distribution with mean 0.01 (Figure 1A, left panel). Specifically, 290 partially overlapping CORUM complexes showed a greater than 0.5 median correlation between their subunits (Table S3). It has been shown that high-stoichiometry interactors are more likely to be coherently expressed across different cell types (Hein et al., 2015); therefore, our correlation data offer an assessment of the stability of known protein complexes in the context of colorectal cancer cells. Moreover, less stable or context-dependent interactions in known protein complexes may be identified by outlier profiles. Such proteins, with at least 50% lower individual correlation compared to the average complex correlation, are highlighted in Table S3. For example, the ORC1 and ORC6 proteins displayed a divergent profile from the average profile of the ORC complex, which is in line with their distinct roles in the replication initiation processes (Ohta et al., 2003; Prasanth et al., 2002).

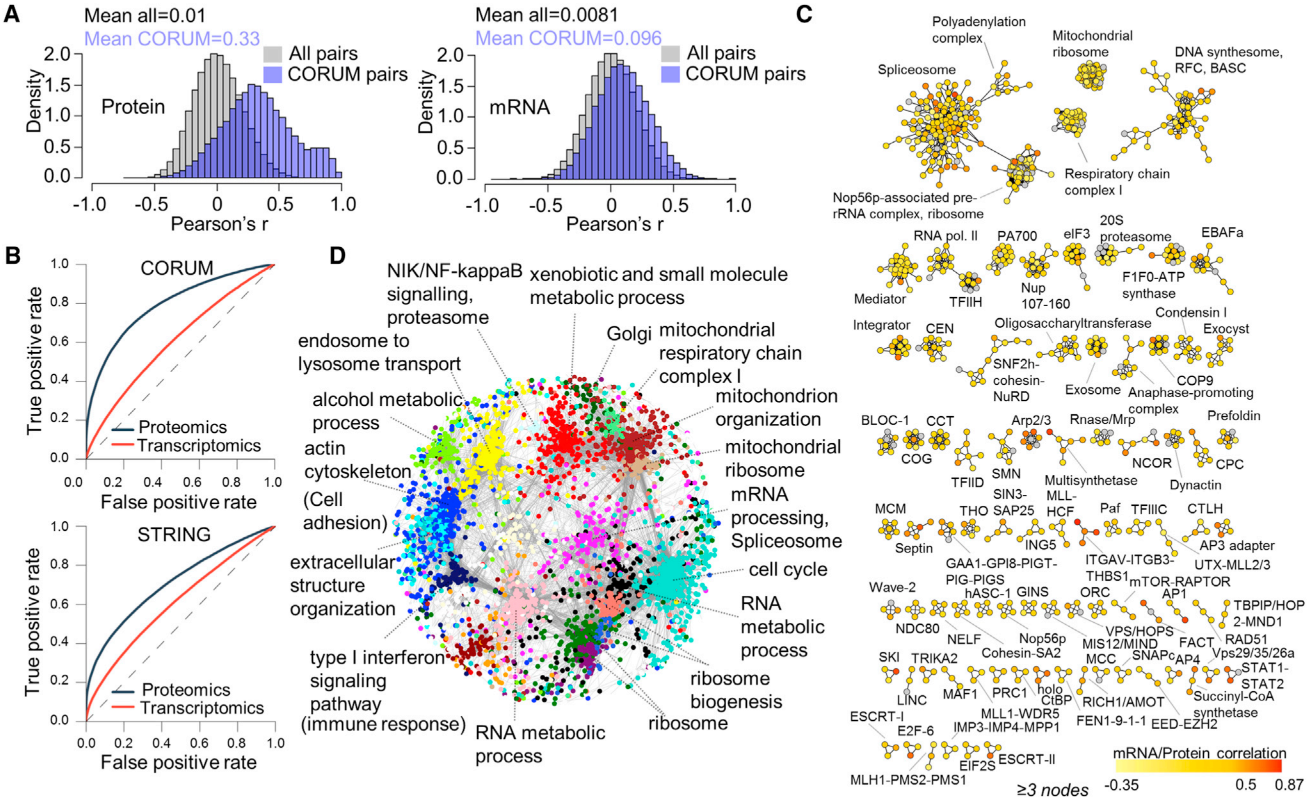

Figure 1. Global Distributions of Gene-to-Gene Correlations and Protein Co-variation Networks in Colorectal Cancer Cell Lines

(A) Distributions of Pearson's correlation coefficients between protein-protein pairs (left panel) and mRNA-mRNA pairs (right panel) for all pairs (gray) and for pairs with known interactions in the CORUM database (blue).

(B) Receiver operating characteristic (ROC) curves illustrating the performance of proteomics- and transcriptomics-based correlations to predict CORUM and high-confident STRING interactions.

(C) Protein abundance correlation networks derived from WGCNA analysis for enriched CORUM complexes. The nodes are color-coded according to mRNA-to-protein Pearson correlation.

(D) The global structure of the WGCNA network using modules with more than 50 nodes. Protein modules are color coded according to the WGCNA module default name, and representative enriched terms are used for the annotation of the network.

See also Figure S3.

In contrast, the distribution of Pearson's coefficients between CORUM pairs based on mRNA co-variation profiles was only slightly shifted toward higher correlations with mean = 0.096 (Figure 1A, right panel). Interestingly, proteins with strong correlations within protein complexes showed low variation across the COREAD panel (Figure S2G) and have poor correspondence to mRNA levels (Figure S2H). Together, these suggest that the subunits of most of the known protein complexes are regulated post-transcriptionally to accurately maintain stable ratio of total abundance. Receiver operating characteristic (ROC) analyses confirmed that our proteomics data outperformed mRNA data in predicting protein complexes as well as high confident STRING interactions (Szklarczyk et al., 2015; CORUM ROC area under the curve [AUC]: 0.79 versus 0.61; STRING ROC AUC: 0.71 versus 0.61; for proteomics and gene expression, respectively; Figure 1B). The ability to also predict any type of STRING interaction suggests that protein co-variation also encompasses a broader range of functional relationships beyond structural physical interactions. Overall, our results demonstrate that correlation analysis of protein abundances across a limited set of cellular samples with variable genotypes can generate co-variation signatures for many known protein-protein interactions and protein complexes.

The Colorectal Cancer Cell Protein Correlation Network

We conducted a systematic un-biased genome-wide analysis to characterize the colorectal cancer cell protein-protein correlation network and to identify de novo modules of interconnected proteins. To this end, we performed a weighted correlation network analysis (WGCNA) (Langfelder and Horvath, 2008) using 8,295 proteins quantified in at least 80% of the cell lines. A total of 284 protein modules ranging in size from 3 to 1,012 proteins ($Q_1 = 6$; $Q_3 = 18$) were inferred covering the entire input dataset. An interaction weight was assigned to each pair of correlating proteins based on their profile similarities and the properties of the network. We performed Gene Ontology annotation of the modules with the WGCNA package as well as using additional terms from CORUM, KEGG, GOBP-slim, GSEA, and Pfam

databases with a Fisher's exact test (Benjamini-Hochberg [Benj. Hoch.] false discovery rate [FDR] < 0.05). We found significantly enriched terms for 235 modules (Table S4) with an average annotation coverage of 40%. Specifically, 111 modules displayed overrepresentation of CORUM protein complexes. For 29 of the 49 not-annotated modules, we detected known STRING interactions within each module, suggesting that these also capture functional associations that do not converge to specific terms.

The correlation networks of protein complexes with more than 2 nodes are shown in Figure 1C. The global structure of the colorectal cancer network comprised of modules with at least 50 proteins is depicted in Figure 1D and is annotated by significant terms. The entire WGCNA network contains 87,420 interactions (weight > 0.02; 96% positive; mean Pearson's r = 0.61), encompassing 7,248 and 20,969 known CORUM and STRING interactions of any confidence, respectively. Overlaying the protein abundance levels on the network generates a unique quantitative map of the cancer cell co-variome, which can help discriminate the different biological characteristics of the cell lines (Figure S3A). For instance, it can be inferred that the CL-40 cell line is mainly characterized by low abundances of cell cycle, ribosomal, and RNA metabolism proteins, which uniquely coincide with increased abundances of immune response proteins (Figure S3A). The full WGCNA network with weights greater than 0.02 is provided in Table S5.

As most of the proteins in modules representing protein complexes are poorly correlated with mRNA levels, we next sought to understand the transcriptional regulation of the modules with the highest mean mRNA-to-protein correlations (5th quantile; mean Pearson's r > 0.57; 41 modules; 1,497 proteins). These included several large components of the co-variome (e.g., "cell adhesion," "small molecule metabolic process," and "innate immune response"), modules showing enrichment for experimental gene sets (based on gene set enrichment analysis [GSEA]), and modules containing proteins encoded by common chromosomal regions, implicating the effects of DNA copy number variations (Figure S3B). In order to further annotate the modules with potential transcriptional regulators, we examined whether transcription factors that are members of the large transcriptionally regulated modules are co-expressed along with their target genes at the protein level. Transcription factor enrichment analysis (Kuleshov et al., 2016) indicated that the "xenobiotic and small molecule metabolic process" module was enriched for the transcription factors HNF4A and CDX2 and that STAT1/STAT2 were the potential master regulators of the "immune response" module (Figure S3B, top left panel). HNF4A (hepatocyte nuclear factor 4-alpha) is an important regulator of metabolism, cell junctions, and the differentiation of intestinal epithelial cells (Garrison et al., 2006) and has been previously associated with colorectal cancer proteomic subtypes in human tumors analyzed by the CPTAC consortium (Zhang et al., 2014). Here, we were able to further characterize the consequences of HNF4A variation through its proteome regulatory network.

To globally understand the interdependencies of protein complexes in the colorectal cancer cells, we plotted the module-to-module relationships as a correlation heatmap using only modules enriched for protein complexes. The representative profile of each module (eigengene or first principal component; Langfelder and Horvath, 2007) was used as a metric (Figure S3C). This analysis captures known functional associations between protein complexes (e.g., MCM-ORC, spliceosome-polyadenylation, and THO-nuclear pore; Lei and Tye, 2001; Millevoi et al., 2006; Wickramasinghe and Laskey, 2015) and reveals the higher order organization of the proteome. The major clusters of the correlation map can be categorized into three main themes: (1) gene expression/splicing/translation/cell cycle; (2) protein processing and trafficking; and (3) mitochondrial functions. This demonstrates that such similarity profiling of abundance signatures has the potential to uncover novel instances of cross-talk between protein complexes and also to discriminate sub-complexes within larger protein assemblies.

In addition to protein abundance co-variation, the scale of global phosphorylation survey accomplished here offers the opportunity for the de novo prediction of kinase-substrate associations inferred by co-varying phosphorylation patterns that involve kinases (Ochoa et al., 2016; Petsalaki et al., 2015). Correlation analysis among 436 phosphopeptides attributed to 137 protein kinases and 29 protein phosphatases yielded 186 positive and 40 negative associations at Benj. Hoch. FDR < 0.1 (Figure S4A), representing the co-phosphorylation signature of kinases and phosphatases in the COREAD panel. Using this high-confidence network as the baseline, we next focused on co-phosphorylation profiling of kinases and phosphatases involved in KEGG signaling pathways (Figure S4B), where known kinase relationships can be used to assess the validity of the predictions. We found co-regulated phosphorylation between RAF1, MAPK1, MAPK3, and RPS6KA3, which were more distantly correlated with the co-phosphorylated BRAF and ARAF protein kinases, all members of the mitogen-activated protein kinase (MAPK) pathway core axis (Figure S4B). MAP2K1 (or MEK1) was found phosphorylated at T388 (unknown kinase substrate), which was not correlating with the above profile. The S222 phosphorylation site of MAP2K2 (or MEK2), regulated by RAF kinase, was not detected possibly due to limitations related to the lengthy (22 amino acids) theoretical overlapping peptide. Strongly maintained co-phosphorylation between CDK1, CDK2, and CDK7 of the cell cycle pathway was another true positive example (Figure S4B). The correlation plots of MAPK1 and MAPK3 phosphorylation and total protein are depicted in Figure S4C, top panel. The co-phosphorylation of BRAF and ARAF is depicted in Figure S4C, bottom left panel. A negative correlation example (between CDK1 kinase and PPP2R5D phosphatase), reflecting the known role of PPP2R5D as an upstream negative regulator of CDK1 (Forester et al., 2007), is shown in Figure S4C, bottom right panel.

Taken together, our correlation analyses reveal the higher-order organization of cellular functions. This well-organized structure is shaped by the compartmental interactions between protein complexes, and it is clearly divided into transcriptionally and post-transcriptionally regulated sectors. The analysis performed here constitutes a reference point for the better understanding of the underlying biological networks in the COREAD panel. The resolution and specificity of the protein clusters can be further improved by the combinatorial use of alternative algorithms for construction of biological networks (Allen et al., 2012).

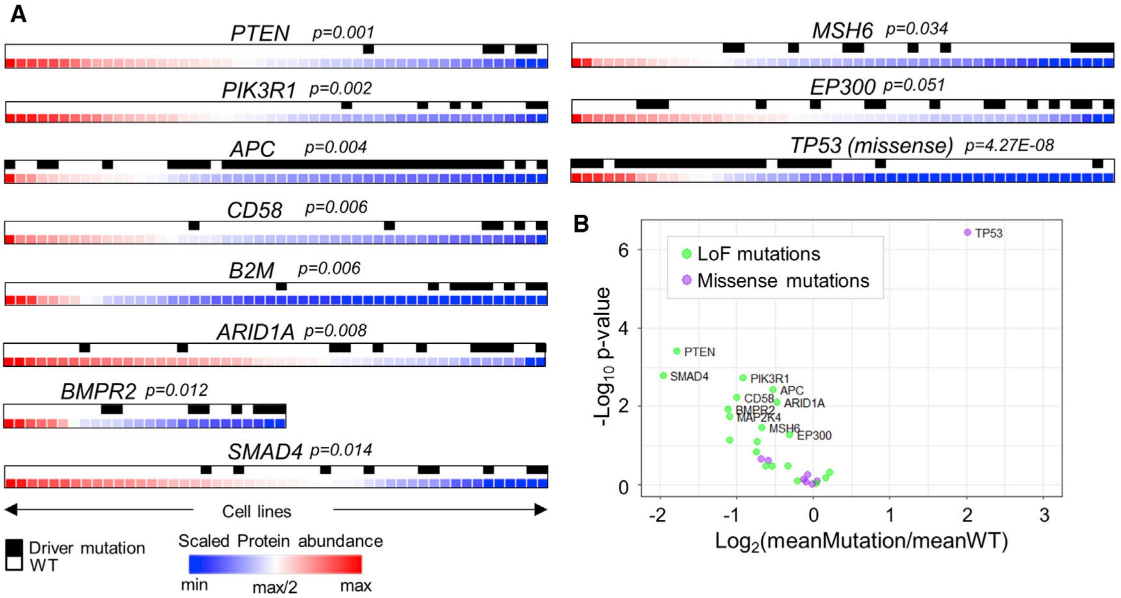

Figure 2. The Effect of Colorectal Cancer Driver Mutations on Protein Abundances

(A) Association of driver mutations in colorectal cancer genes with the respective protein abundance levels (ANOVA test; permutation-based FDR < 0.1). The cell lines are ranked by highest (left) to lowest (right) protein abundance, and the bar on the top indicates the presence of driver mutations with black marks.

(B) Volcano plot summarizing the effect of loss of function (LoF) and missense driver mutations on the respective protein abundances.

Similarly, correlation analysis of protein phosphorylation data demonstrates that functional relationships are encrypted in patterns of co-regulated or anti-regulated phosphorylation events.

The Impact of Genomic Alterations on Protein Abundance

Assessing the impact of non-synonymous protein coding variants and copy number alterations on protein abundance is fundamental in understanding the link between cancer genotypes and dysregulated biological processes. To characterize the impact of genomic alterations on the proteome of the COREAD panel, we first examined whether driver mutations in the most frequently mutated colorectal cancer driver genes (Iorio et al., 2016) could alter the levels of their protein products. For 10 out of 18 such genes harboring driver mutations in at least 5 cell lines (*PTEN*, *PIK3R1*, *APC*, *CD58*, *B2M*, *ARID1A*, *BMPR2*, *SMAD4*, *MSH6*, and *EP300*), we found a significant negative impact on the respective protein abundances, in line with their function as tumor suppressors, whereas missense mutations in *TP53* were associated with elevated protein levels as previously reported (Bertorelle et al., 1996; Dix et al., 1994; ANOVA test; permutation-based FDR < 0.1; Figure 2A). For the majority of driver mutations in oncogenes, there was no clear relationship between the presence of mutations and protein expression (Figure 2B). From these observations, we conclude that mutations in canonical tumor suppressor genes predicted to cause nonsense-mediated decay of transcript generally result in a decrease of protein abundance. This effect, however, varies between the cell lines.

We extended our analysis to globally assess the effect of mutations on protein abundances. For 4,658 genes harboring somatic single-amino-acid substitutions in at least three cell lines, only 12 proteins exhibited differential abundances in the mutated versus the wild-type cell lines at ANOVA test FDR < 0.1 (Figure 3A). Performing the analysis in genes with loss-of-function (LoF) mutations (frameshift, nonsense, in-frame, splice site, and start-stop codon loss) showed that 115 out of the 957 genes tested presented lower abundances in the mutated versus the wild-type cell lines at ANOVA test FDR < 0.1 (Figure 3B). The STRING network of the top significant hits is depicted in Figure 3C and indicates that many of the affected proteins are functionally related. Overall, almost all proteins in a less stringent set with p value < 0.05 (n = 217) were found to be downregulated by LoF mutations, confirming the general negative impact on protein abundances. As expected, zygosity of LoF mutations was a major determinant of protein abundance, with homozygous mutations imposing a more severe downregulation compared to heterozygous mutations (Figure 3D). Whereas the negative impact of LoF mutations was not biased toward their localization in specific protein domains (Figure S5A), we found that mutations localized closer to the protein C terminus were slightly less detrimental (Figure S5B). Notably, genes with LoF mutations and subsequently the significantly affected proteins displayed an overrepresentation of chromatin modification proteins over the identified proteome as the reference set (Fisher's exact test; Benj. Hoch. FDR < 0.05). Chromatin modifiers play an important role in the regulation of chromatin structure during transcription, DNA replication, and DNA repair (Narlikar et al., 2013). Impaired function of chromatin modifiers can lead to dysregulated gene expression and cancer (Cairns, 2001). Our results show that loss of chromatin modification proteins due to the presence of LoF

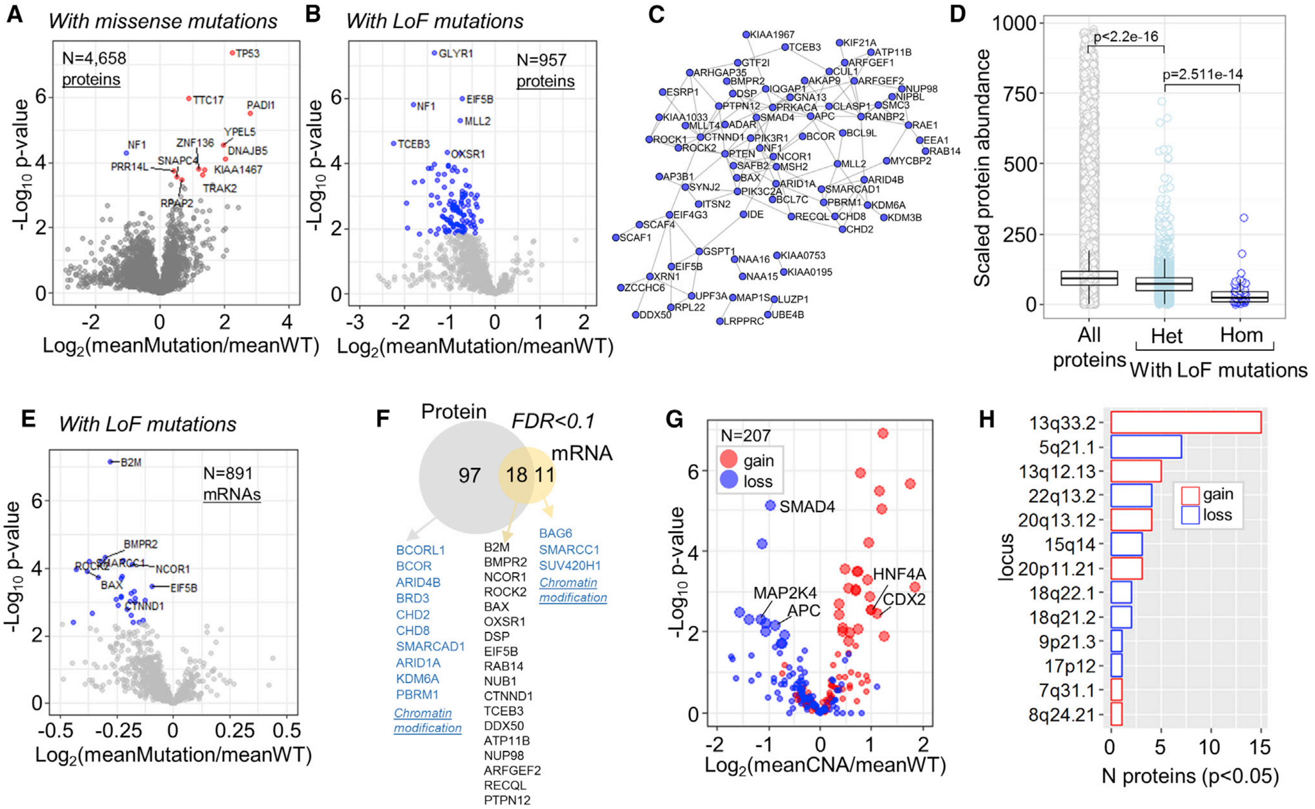

Figure 3. The Global Effects of Genomic Alterations on Protein and mRNA Abundances

(A) Volcano plot summarizing the effect of missense mutations on the respective protein abundances (ANOVA test). Hits at permutation-based FDR < 0.1 are colored.

(B) Volcano plot summarizing the effect of LoF mutations on the respective protein abundances (ANOVA test). Hits at permutation-based FDR < 0.1 are colored.

(C) STRING network of the proteins downregulated by LoF mutations at FDR < 0.1.

(D) Boxplots illustrating the protein abundance differences between all proteins and proteins with heterozygous or homozygous LoF mutations.

(E) Volcano plot summarizing the effect of LoF mutations with both mRNA and protein measurements on the respective mRNA abundances (ANOVA test). Hits at permutation-based FDR < 0.1 are colored.

(F) Venn diagram displaying the overlap between proteins and mRNAs affected by LoF mutations. Selected unique and overlapping proteins are displayed.

(G) Volcano plot summarizing the effect of recurrent copy number alterations on the protein abundances of the contained genes (binary data; ANOVA test). Red and blue points highlight genes with amplifications and losses, respectively. Enlarged points highlight genes at permutation-based FDR < 0.1.

(H) Bar plot illustrating the number of affected proteins by CNAs per genomic locus.

See also Figure S5.

mutations is frequent among the COREAD cell lines and represents a major molecular phenotype.

A less-pronounced impact of LoF mutations was found at the mRNA level, where only 29 genes (out of 891 with both mRNA and protein data) exhibited altered mRNA abundances in the mutated versus the wild-type cell lines at ANOVA test FDR < 0.1 (Figure 3E). The overlap between the protein and mRNA level analyses is depicted in Figure 3F. Even when we regressed out the mRNA levels from the respective protein levels, almost 40% of the proteins previously found to be significantly downregulated were recovered at ANOVA test FDR < 0.1 and the general downregulation trend was still evident (Figure S5C). On the contrary, regression of protein values out of the mRNA values strongly diminished the statistical significance of the associations between mutations and mRNA levels (Figure S5D). The fact that LoF mutations have a greater impact on protein abundances compared to the mRNA levels suggests that an additional post-transcriptional (e.g., translation efficiency) or a post-translational mechanism (e.g., protein degradation) is involved in the regulation of the final protein abundances. Lastly, 24 of the genes downregulated at the protein level by LoF mutations have been characterized as essential genes in human colon cancer cell lines (OGEE database; Chen et al., 2017). Such genes may be used as targets for negative regulation of cancer cell fitness upon further inhibition.

We also explored the effect of 20 recurrent copy number alterations (CNAs), using binary-type data, on the abundances of 207 quantified proteins falling within these intervals (total coverage 56%). Amplified genes tended to display increased protein levels, whereas gene losses had an overall negative impact on protein abundances with several exceptions (Figure 3G). The 49 genes for which protein abundance was

associated with CNAs at ANOVA p value < 0.05 (37 genes at FDR < 0.1) were mapped to 13 genomic loci (Figure 3H), with 13q33.2 amplification encompassing the highest number of affected proteins. Losses in 18q21.2, 5q21.1, and 17p12 loci were associated with reduced protein levels of three important colorectal cancer drivers: SMAD4; APC; and MAP2K4, respectively (FDR < 0.1). Increased levels of CDX2 and HNF4A transcription factors were significantly associated with 13q12.13 and 20q13.12 amplifications (FDR < 0.1). The association of these transcription factors with a number of targets and metabolic processes as found by the co-variome further reveals the functional consequences of the particular amplified loci. All proteins affected by LoF mutations and recurrent CNAs are annotated in Table S1.

Overall, we show that the protein abundance levels of genes with mutations predicted to cause nonsense-mediated mRNA decay are likely to undergo an additional level of negative regulation, which involves translational and/or post-translational events. The extent of protein downregulation heavily depends on zygosity and appears to be independent from secondary structure features and without notable dependency on the position of the mutation on the encoded product. Missense mutations rarely affect the protein abundance levels with the significant exception of TP53. We conclude that only for a small portion of the proteome can the variation in abundance be directly explained by mutations and DNA copy number variations.

The Consequences of Genomic Alterations Extend to Protein Complexes

As tightly controlled maintenance of protein abundance appears to be pivotal for many protein complexes and interactions, we hypothesize that genomic variation can be transmitted from directly affected genes to distant gene protein products through protein interactions, thereby explaining another layer of protein variation. To assess the frequency of such events, we retrieved strongly co-varying interactors of the proteins downregulated by LoF mutations to construct mutation-vulnerable protein networks. For stringency, we filtered for known STRING interactions additionally to the required co-variation. We hypothesize that, in these subnetworks, the downregulation of a protein node due to LoF mutations can also lead to the downregulation of interacting partners. These sub-networks were comprised of 306 protein nodes and 278 interactions and included at least 10 well-known protein complexes (Figure 4A). Two characteristic examples were the BAF and PBAF complexes (Hodges et al., 2016), characterized by disruption of ARID1A, ARID2, and PBRM1 protein abundances. To confirm whether the downregulation of these chromatin-remodeling proteins can affect the protein abundance levels of their co-varying interactors (Figure 4B) post-transcriptionally, we performed proteomics and RNA-seq analysis on CRISPR-Cas9 knockout (KO) clones of these genes in isogenic human iPSCs (Table S6). We found that downregulation of ARID1A protein coincided with diminished protein levels of 7 partners in the predicted network (Figure 4C, left panel). These show the strongest correlations and are known components of the BAF complex (Hodges et al., 2016). In addition, reduced levels of ARID2 resulted in the downregulation of three partners unique to the PBAF complex, with significant loss of PBRM1 protein (Figure 4C, left panel). Several components of the BAF complex were also compromised in the ARID2 KO, reflecting shared components of the BAF and PBAF complexes. Conversely, loss of PBRM1 had no effect on ARID2 protein abundance or any of its module components, in line with the role of PBRM1 in modifying PBAF targeting specificity (Thompson, 2009). The latter demonstrates that collateral effects transmitted through protein interactions can be directional. ARID1A, ARID2, and PBRM1 protein abundance reduction was clearly driven by their respective low mRNA levels; however, the effect was not equally strong in all three genes (Figure 4C, right panel). Strikingly, the interactors that were affected at the protein level were not regulated at the mRNA level, confirming that the regulation of these protein complexes is transcript independent (Figure 4C, right panel). *ARID1A* KO yielded the highest number of differentially expressed genes (Figure 4D); however, these changes were poorly represented in the proteome (Figure 4E). Although pathway-enrichment analysis in all KOs revealed systematic regulation of a wide range of pathways at the protein level, mostly affecting cellular metabolism (Figure 4F), we didn't identify such regulation at the mRNA level. This suggests that the downstream effects elicited by the acquisition of genomic alterations in the particular genes are distinct between gene expression and protein regulation.

The latter prompted us to systematically interrogate the distant effects of all frequent colorectal cancer driver genomic alterations on protein and mRNA abundances by protein and gene expression quantitative trait loci analyses (pQTL and eQTL). We identified 86 proteins and 196 mRNAs with at least one pQTL and eQTL, respectively, at 10% FDR (Figures 5A and S5E). To assess the replication rates between independently tested QTL for each phenotype pair, we also performed the mapping using 6,456 commonly quantified genes at stringent (FDR < 10%) and more relaxed (FDR < 30%) significance cutoffs. In both instances, we found moderate overlap, with 41%–64% of the pQTL validating as eQTLs and 39%–54% of the eQTLs validating as pQTL (Figure 5B). Ranking the pQTL by the number of associations (FDR < 30%) showed that mutations in *BMPR2*, *RNF43*, and *ARID1A*, as well as CNAs of regions 18q22.1, 13q12.13, 16q23.1, 9p21.3, 13q33.2, and 18q21.2 accounted for 62% of the total variant-protein pairs (Figure 5C). The above-mentioned genomic loci were also among the top 10 eQTL hotspots (Figure S5F). High-frequency hotspots in chromosomes 13, 16, and 18 associated with CNAs are consistent with previously identified regions in colorectal cancer tissues (Zhang et al., 2014). We next investigated the pQTL for known associations between the genomic variants and the differentially regulated proteins. Interestingly, increased protein, but not mRNA, levels of the mediator complex subunits were associated with *FBXW7* mutations (Figure S5G), an ubiquitin ligase that targets MED13/13L for degradation (Davis et al., 2013).

Overall, our findings indicate that an additional layer of protein variation can be explained by the collateral effects of mutations on tightly co-regulated partners in protein co-variation networks. Moreover, we show that a large portion of genomic variation affecting gene expression is not directly transmitted to the proteome. Finally, distant protein changes attributed to variation in

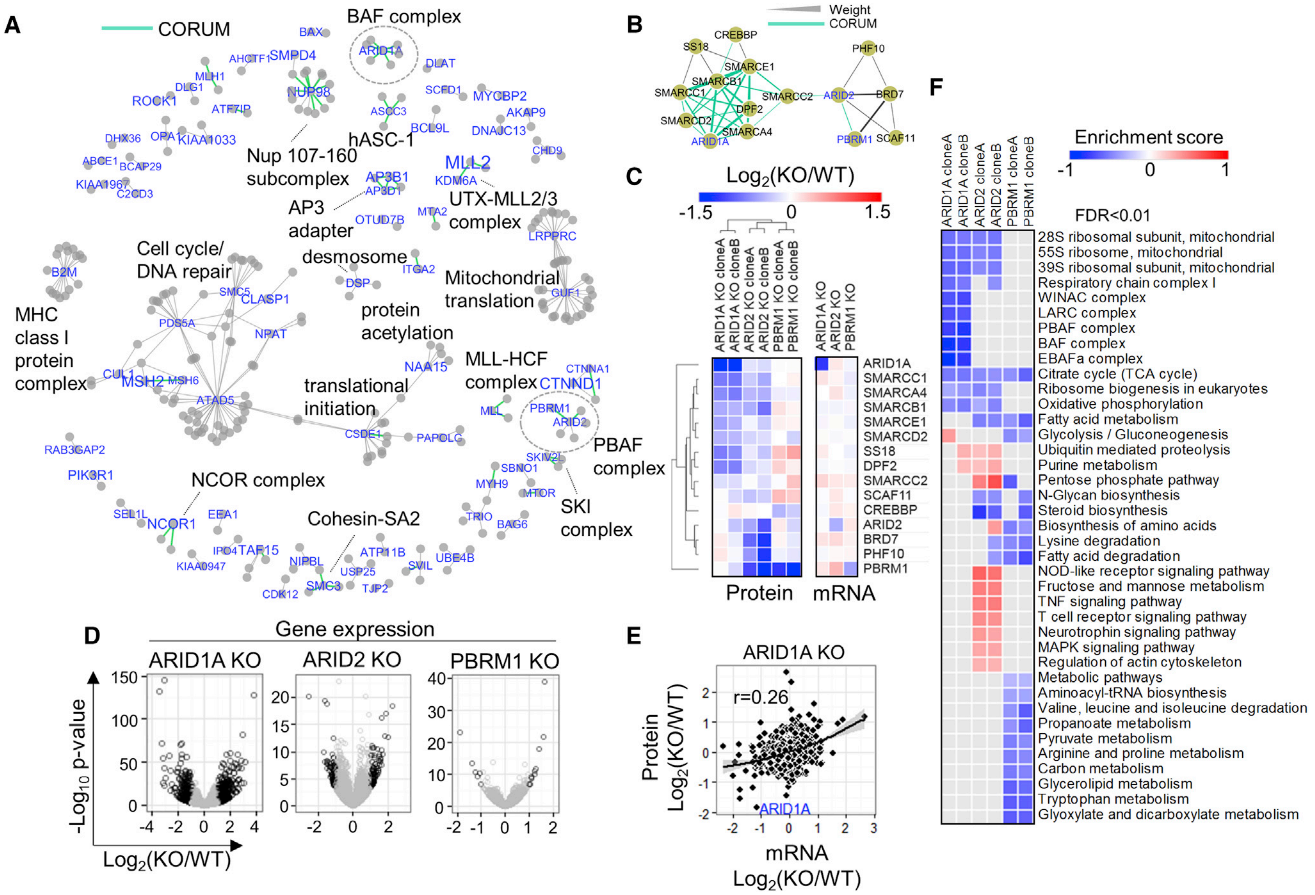

Figure 4. The Consequences of Mutations on Protein Complexes

(A) Correlations networks filtered for known STRING interactions of proteins downregulated by LoF mutations at p value < 0.05. The font size is proportional to the $-\log_{10}$(p value). CORUM interactions are highlighted as green thick edges, and representative protein complexes are labeled.

(B) Protein abundance correlation network of the ARID1A, ARID2, and PBRM1 modules. Green edges denote known CORUM interactions, and the edge thickness is increasing proportionally to the WGCNA interaction weight.

(C) Heatmap summarizing the protein and mRNA abundance $\log_2$fold-change values in the knockout clones compared to the wild-type (WT) clones for the proteins in the ARID1A, ARID2, and PBRM1 modules.

(D) Volcano plots highlighting the differentially regulated mRNAs in the KO samples.

(E) Scatterplot illustrating the correlation between protein and mRNA abundance changes in the ARID1A KO.

(F) KEGG pathway and CORUM enrichment analysis for the proteomic analysis results of ARID1A, ARID2, and PBRM1 CRISPR-cas9 knockouts in human iPSCs.

cancer driver genes can be regulated directly at the protein level with indication of causal effects involving enzyme-substrate relationships.

Proteomic Subtypes of Colorectal Cancer Cell Lines

To explore whether our deep proteomes recapitulate tissue level subtypes of colorectal cancer and to provide insight into the cellular and molecular heterogeneity of the colorectal cancer cell lines, we performed unsupervised clustering based on the quantitative profiles of the top 30% most variable proteins without missing values (n = 2,161) by class discovery using the ConsensusClusterPlus method (Wilkerson and Hayes, 2010). Optimal separation by k-means clustering was reached using 5 colorectal proteomic subtypes (CPSs) (Figures S6A and S6B).

Our proteomic clusters overlapped very well with previously published tissue subtypes and annotations (Medico et al., 2015; Figure S6C), especially with the classification described by De Sousa E Melo et al. (2013). Previous classifiers have commonly subdivided samples along the lines of “epithelial” (lower crypt and crypt top), “microsatellite instability (MSI)-H,” and “stem-like,” with varying descriptions (Guinney et al., 2015). Our in-depth proteomics dataset not only captures the commonly identified classification features but provides increased resolution to further subdivide these groups. The identification of unique proteomic features pointing to key cellular functions gives insight into the molecular basis of these subtypes and provides clarity as to the differences between them (Figures 6A and 6B).

The CPS1 subtype is the canonical MSI-H cluster, overlapping with the CCS2 cluster identified by De Sousa E Melo et al., (2013), CMS1 from Guinney et al., (2015), and CPTAC subtype B (Zhang et al., 2014). Significantly, CPS1 displays low expression of ABC

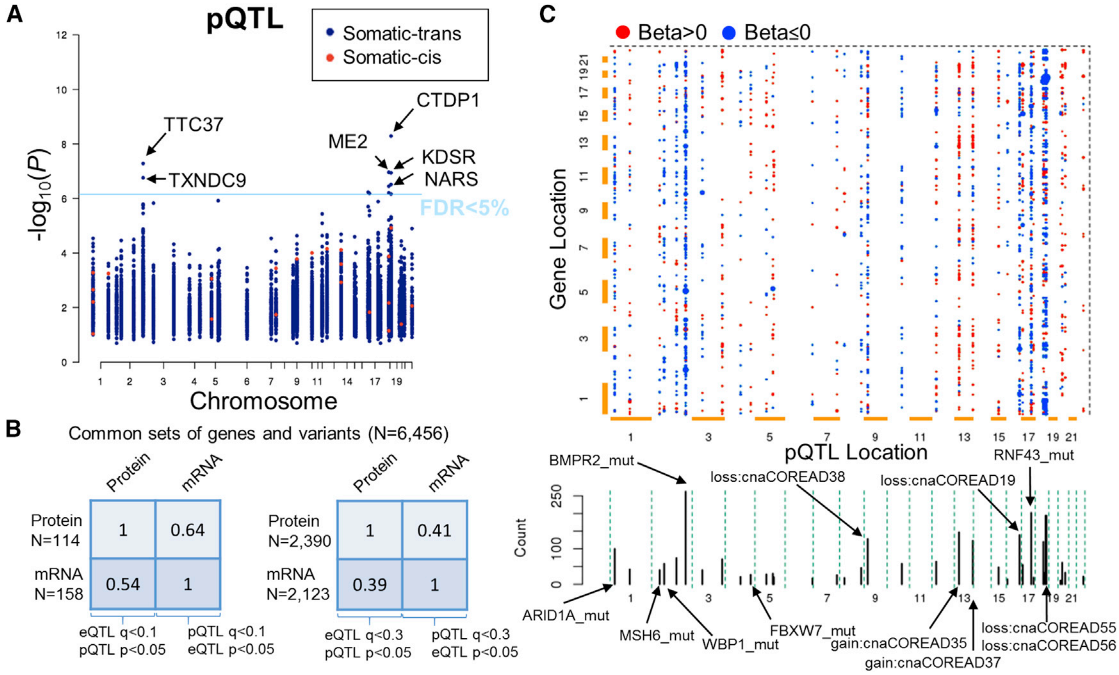

Figure 5. Proteome-wide Quantitative Trait Loci Analysis of Cancer Driver Genomic Alterations

(A) Identification of *cis* and *trans* proteome-wide quantitative trait loci (pQTL) in colorectal cancer cell lines considering colorectal cancer driver variants. The p value and genomic coordinates for the most confident non-redundant protein-variant association tests are depicted in the Manhattan plot.

(B) Replication rates between independently tested QTL for each phenotype pair using common sets of genes and variants (n = 6,456 genes).

(C) Representation of pQTL as 2D plot of variants (x axis) and associated genes (y axis). Associations with q < 0.3 are shown as dots colored by the beta value (blue, negative association; red, positive association) while the size is increasing with the confidence of the association. Cumulative plot of the number of associations per variant is shown below the 2D matrix.

See also Figure S5.

transporters, which may lead to low drug efflux and contribute to the better response rates seen in MSI-H patients (Popat et al., 2005).

Cell lines with a canonical epithelial phenotype (previously classified as CCS1 by De Sousa E Melo et al., 2013) clustered together but are subdivided into 2 subtypes (CPS2 and CPS3). These subtypes displayed higher expression of HNF4A, indicating a more differentiated state. Whereas subtype CPS3 is dominated by transit-amplifying cell phenotypes (Sadanandam et al., 2013), CPS2 is a more heterogeneous group characterized by a mixed TA and goblet cell signature (Figure S6C). CPS2 is also enriched in lines that are hypermutated, including MSI-negative/hypermutated lines (HT115, HCC2998, and HT55; Medico et al., 2015; COSMIC; Figure S6C). However, lower activation of steroid biosynthesis and ascorbate metabolism pathways as well as lower levels of ABC transporters in CPS1 render this group clearly distinguishable from CPS2 (Figure 6B). We also observed subtle differences in the genes mutated between the two groups. *RNF43* mutations and loss of 16q23.1 (including *WWOX* tumor suppressor) are common in CPS1. The separation into two distinct MSI-H/hypermutated classifications was also observed by Guinney et al., (2015) and may have implications for patient therapy and prognosis.

Transit-amplifying subtype CPS3 can be distinguished from CPS2 by lower expression of cell cycle proteins (e.g., CDC20, KIF11, and BUB1); predicted low CDK1, CDK2, and PRKACA kinase activities based on the quantitative profile of known substrates from the PhosphoSitePlus database (Hornbeck et al., 2015); and high PPAR signaling pathway activation (Figure 6B). Common amplifications of 20q13.12 and subsequent high HNF4A levels indicate this cluster corresponds well with CPTAC subtype E (Figure S6D; Zhang et al., 2014). CPS3 also contains lines (DIFI and NCI-H508) that are most sensitive to the anti-epidermal growth factor receptor (EGFR) antibody cetuximab (Medico et al., 2015).

The commonly observed colorectal stem-like subgroup is represented by subtypes CPS4 and CPS5 (Figures 6A and S6C). These cell lines have also been commonly associated with a less-differentiated state by other classifiers, and this is reinforced by our dataset; subtype CPS4 and CPS5 have low levels of HNF4A and CDX1 transcription factors (Chan et al., 2009; Garrison et al., 2006; Jones et al., 2015) and correlate well with CMS4 (Guinney et al., 2015) and CCS3 (De Sousa E Melo et al., 2013). Cells in CPS4 and CPS5 subtypes commonly exhibit loss of the 9p21.3 region, including *CDKN2A* and *CDKN2B*, whereas this is rarely seen in other subtypes. Interestingly, whereas CPS5 displays activation of the Hippo signaling pathway, inflammatory/wounding response, and loss of 18q21.2 (*SMAD4*), CPS4 has a mesenchymal profile, with low expression of CDH1 and JUP similarly to CPTAC subtype C and high Vimentin. Finally, we found

OPEN ACCESS
CellPress

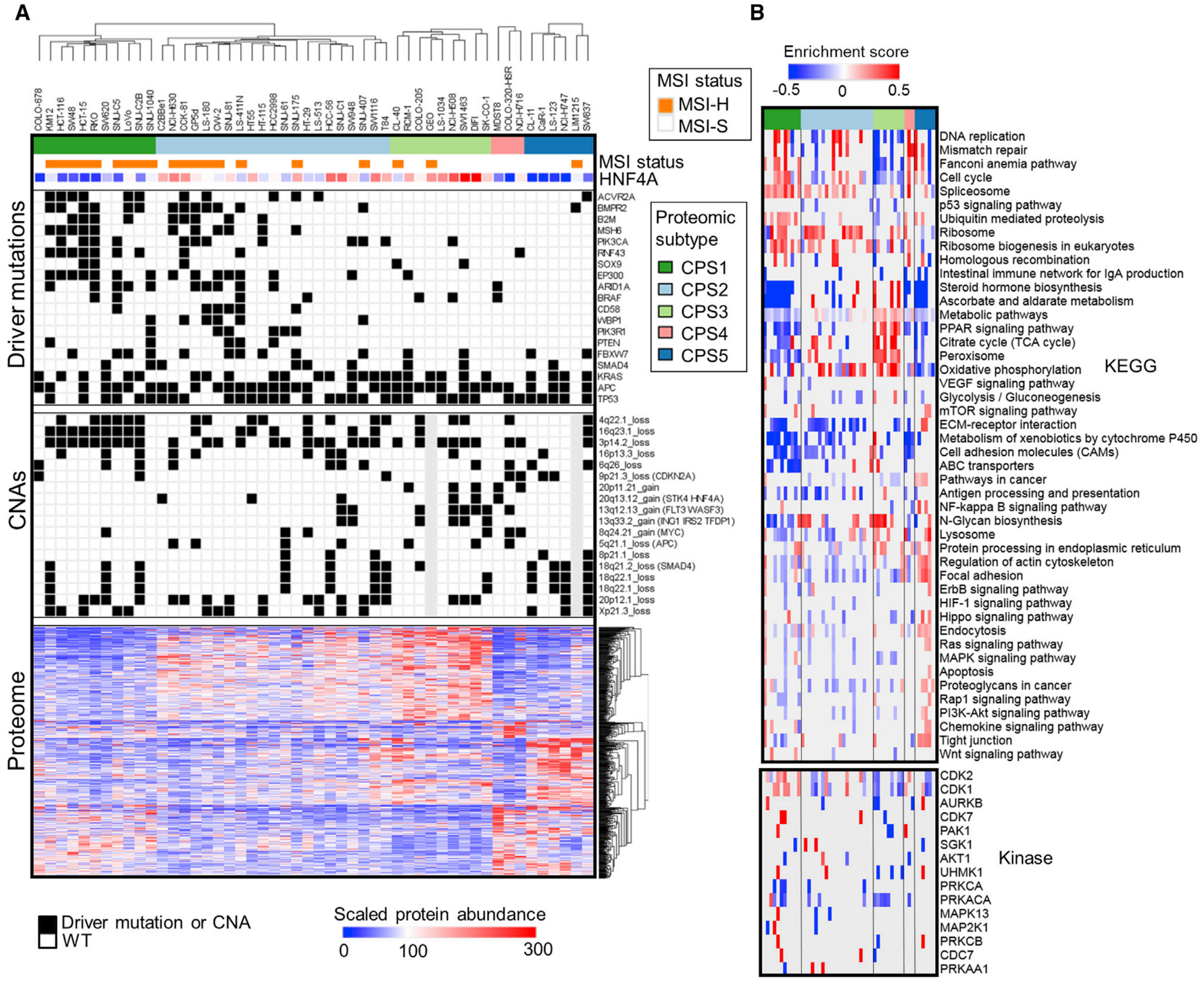

Figure 6. Proteomics Subtypes of Colorectal Cancer Cell Lines and Pathway Analysis

(A) Cell lines are represented as columns, horizontally ordered by five color-coded proteomics consensus clusters and aligned with microsatellite instability (MSI), HNF4A protein abundance, cancer driver genomic alterations, and differentially regulated proteins.

(B) KEGG pathway and kinase enrichment analysis per cell line.

See also Figure S6.

common systematic patterns between the COREAD proteomic subtypes and the CPTAC colorectal cancer proteomic subtypes (Zhang et al., 2014) in a global scale (Figures S6D and S6E) using the cell line signature proteins. The overlap between the cell lines and the CPTAC colorectal tissue proteomic subtypes is summarized in Figure S6F.

Lastly, we detected 206 differentially regulated proteins between the MSI-high and MSI-low cell lines (Welch's t test; permutation based FDR < 0.1; Figure S7A), which were mainly converging to downregulation of DNA repair and chromosome organization as well as to upregulation of proteasome and Lsm2-8 complex (RNA degradation; Figure S7B). Whereas loss of DNA repair and organization functions are the underlying causes of MSI (Boland and Goel, 2010), the upregulation of RNA and protein degradation factors indicate the activation of a scavenging mechanism that regulates the abundance of mutated gene products.

Pharmacoproteomic Models Significantly Contribute to Drug Response Prediction

Although a number of recent studies have investigated the power of different combinations of molecular data to predict drug response in colorectal cancer cell lines, these have been limited to using genomic (mutations and copy number), transcriptomic, and methylation datasets (Iorio et al., 2016). We have shown above that the DNA and gene expression variations are not directly consistent with the protein measurements. Also, it has been shown that there is a gain in predictive power for some

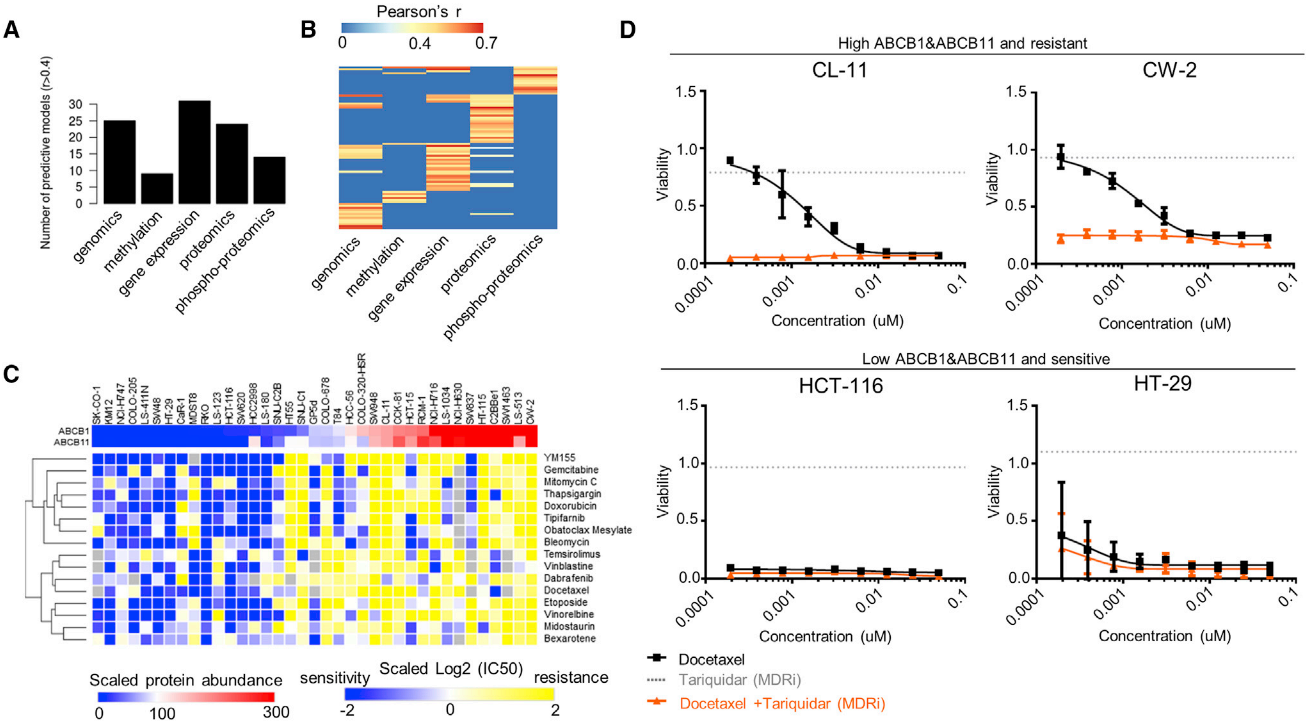

Figure 7. Pharmacoproteomic Models

(A) The number of drugs for which predictive models (i.e., models where the Pearson correlation between predicted and observed IC_{50}s exceeds $r > 0.4$) could be fitted is stratified per data type.

(B) Heatmap indicating for each drug and each data type whether a predictive model could be fitted. Most drugs were specifically predicted by one data type.

(C) Heatmap of scaled log2 IC_{50} values for selected drugs displaying significant association (ANOVA FDR < 0.05) between protein abundance of ABCB1, ABCB11, and drug response.

(D) Dose-response profiles for colorectal cancer cell lines treated with docetaxel (black line), 2.5 μM tariquidar alone (gray dotted line), or the combination of docetaxel and 2.5 μM tariquidar (orange line). Error bars represent mean ± SEM.

See also Figure S7.

phenotypic associations when also using protein abundance and phosphorylation changes (Costello et al., 2014; Gholami et al., 2013; Li et al., 2017). To date, there has not been a comprehensive analysis of the effect on the predictive power from the addition of proteomics datasets in colorectal cancer. All of the colorectal cell lines included in this study have been extensively characterized by sensitivity data (half maximal inhibitory concentration [IC_{50}] values) for 265 compounds (Iorio et al., 2016). These include clinical drugs (n = 48), drugs currently in clinical development (n = 76), and experimental compounds (n = 141).

We built Elastic Net models that use as input features genomic (mutations and copy number gains/losses), methylation (CpG islands in gene promoters), gene expression, proteomics, and phosphoproteomics datasets. We were able to generate predictive models where the Pearson correlation between predicted and observed IC_{50} was greater than 0.4 in 81 of the 265 compounds (Table S7). Response to most drugs was often specifically predicted by one data type, with very little overlap (Figures 7A and 7B, respectively). The number of predictive models per drug target pathway and data type is depicted in Figure S7C, highlighting the contribution of proteomics and phosphoproteomics datasets in predicting response to certain drug classes.

Within the proteomics-based signatures found to be predictive for drug response, we frequently observed the drug efflux transporters ABCB1 and ABCB11 (6 and 6 out of 24, respectively; 8 non-redundant; Table S7). In all models containing these proteins, elevated expression of the drug transporter was associated with drug resistance, in agreement with previous results (Garnett et al., 2012). Notably, protein measurements of these transporters correlated more strongly with response to these drugs than the respective mRNA measurements (mean Pearson's $r = 0.61$ and $r = 0.31$, respectively; Wilcoxon test p value = 0.016). Interestingly, ABCB1 and ABCB11 are tightly co-regulated (Pearson's $r = 0.92$), suggesting a novel protein interaction. Classifying the cell lines into two groups with low and high mean protein abundance of ABCB1 and ABCB11 revealed a strong overlap with drug response for 54 compounds (ANOVA test; permutation-based FDR < 0.05). Representative examples of these drug associations are shown in Figure 7C. To confirm the causal association between the protein abundance levels of ABCB1, ABCB11, and drug response, we performed viability assays in four cell lines treated with docetaxel, a chemotherapeutic agent broadly used in cancer treatment. The treatments were performed in the presence or absence of an ABCB1 inhibitor (tariquidar) and confirmed that ABCB1 inhibition increases sensitivity

to docetaxel (Figure 7D) in the cell lines with high ABCB1 and ABCB11 levels. Given the dominant effect of the drug efflux proteins in drug response, we next tested whether additional predictive models could be identified by correcting the drug response data for the mean protein abundance of ABCB1 and ABCB11 using linear regression. With this analysis, we were able to generate predictive models for 41 additional drugs (total 57) from all input datasets combined (Figure S7D; Table S7). Taken together, our results show that the protein expression levels of drug efflux pumps play a key role in determining drug response, and whereas predictive genomic biomarkers may still be discovered, the importance of proteomic associations with drug response should not be underestimated.

DISCUSSION

Our analysis of colorectal cancer cells using in-depth proteomics has yielded several significant insights into both fundamental molecular cell biology and the molecular heterogeneity of colorectal cancer subtypes. Beyond static measurements of protein abundances, the quality of our dataset enabled the construction of a reference proteomic co-variation map with topological features capturing the interplay between known protein complexes and biological processes in colorectal cancer cells. We show that the subunits of protein complexes tend to tightly maintain their total abundance ratios post-transcriptionally, and this is a fundamental feature of the co-variation network. The primary level of co-variation between proteins enables the generation of unique abundance profiles of known protein interactions, and the secondary level of co-regulation between protein complexes can indicate the formation of multi-complex protein assemblies. Moreover, the identification of proteins with outlier profiles from the conserved profile of their known interactors within a given complex can point to their pleiotropic roles in the associated processes. Notably, our approach can be used in combination with high-throughput pull-down assays (Hein et al., 2015; Huttlin et al., 2015) for further refinement of large-scale protein interactomes based on co-variation signatures that appear to be pivotal for many protein interactions. Additionally, our approach can serve as a time-effective tool for the identification of tissue-specific co-variation profiles in cancer that may reflect tissue-specific associations. As a perspective, our data may be used in combination with genetic interaction screens (Costanzo et al., 2016) to explore whether protein co-regulation can explain or predict synthetic lethality (Kaelin, 2005). Another novel aspect that emerged from our analysis is the maintenance of co-regulation at the level of net protein phosphorylation. This seems to be more pronounced in signaling pathways, where the protein abundances are insufficient to indicate functional associations. Analogous study of co-regulation between different types of protein modifications could also enable the identification of modification cross-talk (Beltrao et al., 2013). This framework also enabled the identification of upstream regulatory events that link transcription factors to their transcriptional targets at the protein level and partially explained the components of the co-variome that are not strictly shaped by physical protein interactions. To a smaller degree, the module-based analysis was predictive of DNA copy number variations, exposing paradigms of simple cause-and-effect proteogenomic features of the cell lines. Such associations should be carefully taken into consideration in large-scale correlation analyses, as they do not necessarily represent functional relationships.

The simplification of the complex proteomic landscapes into co-variation modules enables a more direct alignment of genomic features with cellular functions and delineates how genomic alterations affect the proteome directly and indirectly. We show that LoF mutations can have a direct negative impact on protein abundances further to mRNA regulation. Targeted deletion of key chromatin modifiers by CRISPR/cas9 followed by proteomics and RNA-seq analysis confirmed that the effects of genomic alterations can propagate through physical protein interactions, highlighting the role of translational or post-translational mechanisms in modulating protein co-variation. Additionally, our analysis indicated that directionality can be another characteristic of such interactions.

We provide evidence that colorectal cancer subtypes derived from tissue level gene expression and proteomics datasets are largely recapitulated in cell-based model systems at the proteome level, which further resolves the main subtypes into groups. This classification reflects a possible cell type of origin and the underlying differences in genomic alterations. This robust functional characterization of the COREAD cell lines can guide cell line selection in targeted cellular and biochemical experimental designs, where cell-line-specific biological features can have an impact on the results. Proteomic analysis highlighted that the expression of key protein components, such as ABC transporters, is critical in predicting drug response in colorectal cancer. Whereas further work is required to establish these as validated biomarkers of patient response in clinical trials, numerous studies have noted the role of these channels in aiding drug efflux (Chen et al., 2016). In summary, this study demonstrates the utility of proteomics in different aspects of systems biology and provides a valuable insight into the regulatory variation in colorectal cancer cells.

EXPERIMENTAL PROCEDURES

Sample Preparation and Analysis

Cell pellets were lysed by probe sonication/boiling, and protein extracts were subjected to trypsin digestion. The tryptic peptides were labeled with the TMT10plex reagents, combined at equal amounts, and fractionated with high-pH C18 high-performance liquid chromatography (HPLC). Phosphopeptide enrichment was performed with immobilized metal ion affinity chromatography (IMAC). LC-MS analysis was performed on the Dionex Ultimate 3000 system coupled with the Orbitrap Fusion Mass Spectrometer. MS3 level quantification with Synchronous Precursor Selection was used for total proteome measurements, whereas phosphopeptide measurements were obtained with a collision-induced dissociation-higher energy collisional dissociation (CID-HCD) method at the MS2 level. Raw mass spectrometry files were subjected to database search and quantification in Proteome Discoverer 1.4 or 2.1 using the SequestHT node followed by Percolator validation. Protein and phosphopeptide quantification was obtained by the sum of column-normalized TMT spectrum intensities followed by row-mean scaling.

Statistical Analysis

Enrichment for biological terms, pathways, and kinases was performed in Perseus 1.4 software with Fisher's test or with the 1D-annotation-enrichment method. Known kinase-substrate associations were downloaded from the

PhosphoSitePlus database. All terms were filtered for Benjamini-Hochberg FDR < 0.05 or FDR < 0.1. Correlation analyses were performed in RStudio with Benjamini-Hochberg multiple testing correction. ANOVA and Welch's tests were performed in Perseus 1.4 software. Permutation-based FDR correction was applied to the ANOVA test p values for the assessment of the impact of mutations and copy number variations on protein and mRNA abundances. Volcano plots, boxplots, distribution plots, scatterplots, and bar plots were drawn in RStudio with the ggplot2 and ggrepel packages. All QTL associations were implemented by LIMIX using a linear regression test.

ACCESSION NUMBERS

The accession number for the mass spectrometry proteomics data reported in this paper is PRIDE: PXD005235. The accession number for the CRISPR-cas9 RNA-seq data reported in this paper is European Nucleotide Archive: EGAS00001002262. The accession number for the quantitative proteomics data reported in this paper is Expression Atlas: E-PROT-6.

SUPPLEMENTAL INFORMATION

Supplemental Information includes Supplemental Experimental Procedures, seven figures, and seven tables and can be found with this article online at http://dx.doi.org/10.1016/j.celrep.2017.08.010.

AUTHOR CONTRIBUTIONS

Conceptualization, J.S.C. and U.M.; Methodology, T.I.R., S.P.W., and J.S.C.; Cell Lines, S.P. and S.P.W.; Mass Spectrometry, T.I.R. and L.Y.; Data Analysis, T.I.R., E.G., F.Z.G., S.P.W., J.C.W., M.P., P.B., J.S.-R., A.B., and O.S.; Cell Lines Classification, R.D. and J.G.; Drug Data Analysis, N.A., M.M., M.S., M.Y., J.S.-R., S.P.W., T.I.R., L.W., and U.M.; CRISPR Lines RNA-Seq, C.A., M.D.C.V.-H., and D.J.A.; Writing – Original Draft, T.I.R., S.P.W., L.W., U.M., and J.S.C.; Writing – Review and Editing, all.

ACKNOWLEDGMENTS

The work performed at the Sanger Institute was funded by a core grant from the Wellcome Trust (098051). S.P.W., C.A., D.J.A., N.A., M.M., and L.W. are funded by the European Research Council under the European Union's Seventh Framework Programme (FP7/2007-2013)/ERC synergy grant agreement no. 319661 COMBATCANCER. The work of R.D. and J.G. was supported in part by the Merck KGaA, Darmstadt, Germany (Grant for Oncology Innovation 2015). We thank Christoph Schlaffner for generating the data file formats compatible with genomic browsers and the Expression Atlas team for making the proteomics data publicly available in Expression Atlas and ArrayExpress repositories. We would like to thank members of the Cancer Genome Project for helpful discussions and Sarah A. Teichmann for discussions and general suggestions about the manuscript.

Received: December 9, 2016
Revised: July 6, 2017
Accepted: July 24, 2017
Published: August 29, 2017

REFERENCES

Aken, B.L., Achuthan, P., Akanni, W., Amode, M.R., Bernsdorff, F., Bhai, J., Billis, K., Carvalho-Silva, D., Cummins, C., Clapham, P., et al. (2017). Ensembl 2017. Nucleic Acids Res. *45* (*D1*), D635–D642.

Allen, J.D., Xie, Y., Chen, M., Girard, L., and Xiao, G. (2012). Comparing statistical methods for constructing large scale gene networks. PLoS ONE *7*, e29348.

Beltrao, P., Bork, P., Krogan, N.J., and van Noort, V. (2013). Evolution and functional cross-talk of protein post-translational modifications. Mol. Syst. Biol. *9*, 714.

Bertorelle, R., Esposito, G., Belluco, C., Bonaldi, L., Del Mistro, A., Nitti, D., Lise, M., and Chieco-Bianchi, L. (1996). p53 gene alterations and protein accumulation in colorectal cancer. Clin. Mol. Pathol. *49*, M85–M90.

Boland, C.R., and Goel, A. (2010). Microsatellite instability in colorectal cancer. Gastroenterology *138*, 2073–2087.e3.

Cairns, B.R. (2001). Emerging roles for chromatin remodeling in cancer biology. Trends Cell Biol. *11*, S15–S21.

Chan, C.W.M., Wong, N.A., Liu, Y., Bicknell, D., Turley, H., Hollins, L., Miller, C.J., Wilding, J.L., and Bodmer, W.F. (2009). Gastrointestinal differentiation marker Cytokeratin 20 is regulated by homeobox gene CDX1. Proc. Natl. Acad. Sci. USA *106*, 1936–1941.

Chen, Z., Shi, T., Zhang, L., Zhu, P., Deng, M., Huang, C., Hu, T., Jiang, L., and Li, J. (2016). Mammalian drug efflux transporters of the ATP binding cassette (ABC) family in multidrug resistance: A review of the past decade. Cancer Lett. *370*, 153–164.

Chen, W.H., Lu, G., Chen, X., Zhao, X.M., and Bork, P. (2017). OGEE v2: an update of the online gene essentiality database with special focus on differentially essential genes in human cancer cell lines. Nucleic Acids Res. *45* (*D1*), D940–D944.

Costanzo, M., VanderSluis, B., Koch, E.N., Baryshnikova, A., Pons, C., Tan, G., Wang, W., Usaj, M., Hanchard, J., Lee, S.D., et al. (2016). A global genetic interaction network maps a wiring diagram of cellular function. Science *353*, aaf1420.

Costello, J.C., Heiser, L.M., Georgii, E., Gönen, M., Menden, M.P., Wang, N.J., Bansal, M., Ammad-ud-din, M., Hintsanen, P., Khan, S.A., et al.; NCI DREAM Community (2014). A community effort to assess and improve drug sensitivity prediction algorithms. Nat. Biotechnol. *32*, 1202–1212.

Davis, M.A., Larimore, E.A., Fissel, B.M., Swanger, J., Taatjes, D.J., and Clurman, B.E. (2013). The SCF-Fbw7 ubiquitin ligase degrades MED13 and MED13L and regulates CDK8 module association with Mediator. Genes Dev. *27*, 151–156.

De Sousa E Melo, F., Wang, X., Jansen, M., Fessler, E., Trinh, A., de Rooij, L.P., de Jong, J.H., de Boer, O.J., van Leersum, R., Bijlsma, M.F., et al. (2013). Poor-prognosis colon cancer is defined by a molecularly distinct subtype and develops from serrated precursor lesions. Nat. Med. *19*, 614–618.

Dix, B., Robbins, P., Carrello, S., House, A., and Iacopetta, B. (1994). Comparison of p53 gene mutation and protein overexpression in colorectal carcinomas. Br. J. Cancer *70*, 585–590.

Forester, C.M., Maddox, J., Louis, J.V., Goris, J., and Virshup, D.M. (2007). Control of mitotic exit by PP2A regulation of Cdc25C and Cdk1. Proc. Natl. Acad. Sci. USA *104*, 19867–19872.

Garnett, M.J., Edelman, E.J., Heidorn, S.J., Greenman, C.D., Dastur, A., Lau, K.W., Greninger, P., Thompson, I.R., Luo, X., Soares, J., et al. (2012). Systematic identification of genomic markers of drug sensitivity in cancer cells. Nature *483*, 570–575.

Garrison, W.D., Battle, M.A., Yang, C., Kaestner, K.H., Sladek, F.M., and Duncan, S.A. (2006). Hepatocyte nuclear factor 4alpha is essential for embryonic development of the mouse colon. Gastroenterology *130*, 1207–1220.

Gholami, A.M., Hahne, H., Wu, Z., Auer, F.J., Meng, C., Wilhelm, M., and Kuster, B. (2013). Global proteome analysis of the NCI-60 cell line panel. Cell Rep. *4*, 609–620.

Guinney, J., Dienstmann, R., Wang, X., de Reyniès, A., Schlicker, A., Soneson, C., Marisa, L., Roepman, P., Nyamundanda, G., Angelino, P., et al. (2015). The consensus molecular subtypes of colorectal cancer. Nat. Med. *21*, 1350–1356.

Hein, M.Y., Hubner, N.C., Poser, I., Cox, J., Nagaraj, N., Toyoda, Y., Gak, I.A., Weisswange, I., Mansfeld, J., Buchholz, F., et al. (2015). A human interactome in three quantitative dimensions organized by stoichiometries and abundances. Cell *163*, 712–723.

Hodges, C., Kirkland, J.G., and Crabtree, G.R. (2016). The many roles of BAF (mSWI/SNF) and PBAF complexes in cancer. Cold Spring Harb. Perspect. Med. *6*, a026930.

Hornbeck, P.V., Zhang, B., Murray, B., Kornhauser, J.M., Latham, V., and Skrzypek, E. (2015). PhosphoSitePlus, 2014: mutations, PTMs and recalibrations. Nucleic Acids Res. *43*, D512–D520.

Huttlin, E.L., Ting, L., Bruckner, R.J., Gebreab, F., Gygi, M.P., Szpyt, J., Tam, S., Zarraga, G., Colby, G., Baltier, K., et al. (2015). The BioPlex network: a systematic exploration of the human interactome. Cell *162*, 425–440.

Iorio, F., Knijnenburg, T.A., Vis, D.J., Bignell, G.R., Menden, M.P., Schubert, M., Aben, N., Gonçalves, E., Barthorpe, S., Lightfoot, H., et al. (2016). A landscape of pharmacogenomic interactions in cancer. Cell *166*, 740–754.

Jones, M.F., Hara, T., Francis, P., Li, X.L., Bilke, S., Zhu, Y., Pineda, M., Subramanian, M., Bodmer, W.F., and Lal, A. (2015). The CDX1-microRNA-215 axis regulates colorectal cancer stem cell differentiation. Proc. Natl. Acad. Sci. USA *112*, E1550–E1558.

Kaelin, W.G., Jr. (2005). The concept of synthetic lethality in the context of anticancer therapy. Nat. Rev. Cancer *5*, 689–698.

Kent, W.J., Sugnet, C.W., Furey, T.S., Roskin, K.M., Pringle, T.H., Zahler, A.M., and Haussler, D. (2002). The human genome browser at UCSC. Genome Res. *12*, 996–1006.

Kuleshov, M.V., Jones, M.R., Rouillard, A.D., Fernandez, N.F., Duan, Q., Wang, Z., Koplev, S., Jenkins, S.L., Jagodnik, K.M., Lachmann, A., et al. (2016). Enrichr: a comprehensive gene set enrichment analysis web server 2016 update. Nucleic Acids Res. *44* (*W1*), W90–W97.

Langfelder, P., and Horvath, S. (2007). Eigengene networks for studying the relationships between co-expression modules. BMC Syst. Biol. *1*, 54.

Langfelder, P., and Horvath, S. (2008). WGCNA: an R package for weighted correlation network analysis. BMC Bioinformatics *9*, 559.

Lei, M., and Tye, B.K. (2001). Initiating DNA synthesis: from recruiting to activating the MCM complex. J. Cell Sci. *114*, 1447–1454.

Li, J., Zhao, W., Akbani, R., Liu, W., Ju, Z., Ling, S., Vellano, C.P., Roebuck, P., Yu, Q., Eterovic, A.K., et al. (2017). Characterization of human cancer cell lines by reverse-phase protein arrays. Cancer Cell *31*, 225–239.

McAlister, G.C., Nusinow, D.P., Jedrychowski, M.P., Wühr, M., Huttlin, E.L., Erickson, B.K., Rad, R., Haas, W., and Gygi, S.P. (2014). MultiNotch MS3 enables accurate, sensitive, and multiplexed detection of differential expression across cancer cell line proteomes. Anal. Chem. *86*, 7150–7158.

Medico, E., Russo, M., Picco, G., Cancelliere, C., Valtorta, E., Corti, G., Buscarino, M., Isella, C., Lamba, S., Martinoglio, B., et al. (2015). The molecular landscape of colorectal cancer cell lines unveils clinically actionable kinase targets. Nat. Commun. *6*, 7002.

Mertins, P., Mani, D.R., Ruggles, K.V., Gillette, M.A., Clauser, K.R., Wang, P., Wang, X., Qiao, J.W., Cao, S., Petralia, F., et al.; NCI CPTAC (2016). Proteogenomics connects somatic mutations to signalling in breast cancer. Nature *534*, 55–62.

Millevoi, S., Loulergue, C., Dettwiler, S., Karaa, S.Z., Keller, W., Antoniou, M., and Vagner, S. (2006). An interaction between U2AF 65 and CF I(m) links the splicing and 3′ end processing machineries. EMBO J. *25*, 4854–4864.

Narlikar, G.J., Sundaramoorthy, R., and Owen-Hughes, T. (2013). Mechanisms and functions of ATP-dependent chromatin-remodeling enzymes. Cell *154*, 490–503.

Ochoa, D., Jonikas, M., Lawrence, R.T., El Debs, B., Selkrig, J., Typas, A., Villén, J., Santos, S.D., and Beltrao, P. (2016). An atlas of human kinase regulation. Mol. Syst. Biol. *12*, 888.

Ohta, S., Tatsumi, Y., Fujita, M., Tsurimoto, T., and Obuse, C. (2003). The ORC1 cycle in human cells: II. Dynamic changes in the human ORC complex during the cell cycle. J. Biol. Chem. *278*, 41535–41540.

Petryszak, R., Keays, M., Tang, Y.A., Fonseca, N.A., Barrera, E., Burdett, T., Füllgrabe, A., Fuentes, A.M., Jupp, S., Koskinen, S., et al. (2016). Expression Atlas update–an integrated database of gene and protein expression in humans, animals and plants. Nucleic Acids Res. *44* (*D1*), D746–D752.

Petsalaki, E., Helbig, A.O., Gopal, A., Pasculescu, A., Roth, F.P., and Pawson, T. (2015). SELPHI: correlation-based identification of kinase-associated networks from global phospho-proteomics data sets. Nucleic Acids Res. *43* (*W1*), W276–W282.

Popat, S., Hubner, R., and Houlston, R.S. (2005). Systematic review of microsatellite instability and colorectal cancer prognosis. J. Clin. Oncol. *23*, 609–618.

Prasanth, S.G., Prasanth, K.V., and Stillman, B. (2002). Orc6 involved in DNA replication, chromosome segregation, and cytokinesis. Science *297*, 1026–1031.

Robinson, J.T., Thorvaldsdóttir, H., Winckler, W., Guttman, M., Lander, E.S., Getz, G., and Mesirov, J.P. (2011). Integrative genomics viewer. Nat. Biotechnol. *29*, 24–26.

Ruepp, A., Waegele, B., Lechner, M., Brauner, B., Dunger-Kaltenbach, I., Fobo, G., Frishman, G., Montrone, C., and Mewes, H.W. (2010). CORUM: the comprehensive resource of mammalian protein complexes–2009. Nucleic Acids Res. *38*, D497–D501.

Sadanandam, A., Lyssiotis, C.A., Homicsko, K., Collisson, E.A., Gibb, W.J., Wullschleger, S., Ostos, L.C.G., Lannon, W.A., Grotzinger, C., Del Rio, M., et al. (2013). A colorectal cancer classification system that associates cellular phenotype and responses to therapy. Nat. Med. *19*, 619–625.

Stefely, J.A., Kwiecien, N.W., Freiberger, E.C., Richards, A.L., Jochem, A., Rush, M.J.P., Ulbrich, A., Robinson, K.P., Hutchins, P.D., Veling, M.T., et al. (2016). Mitochondrial protein functions elucidated by multi-omic mass spectrometry profiling. Nat. Biotechnol. *34*, 1191–1197.

Szklarczyk, D., Franceschini, A., Wyder, S., Forslund, K., Heller, D., Huerta-Cepas, J., Simonovic, M., Roth, A., Santos, A., Tsafou, K.P., et al. (2015). STRING v10: protein-protein interaction networks, integrated over the tree of life. Nucleic Acids Res. *43*, D447–D452.

Thompson, M. (2009). Polybromo-1: the chromatin targeting subunit of the PBAF complex. Biochimie *91*, 309–319.

Wang, J., Ma, Z., Carr, S.A., Mertins, P., Zhang, H., Zhang, Z., Chan, D.W., Ellis, M.J., Townsend, R.R., Smith, R.D., et al. (2017). Proteome profiling outperforms transcriptome profiling for coexpression based gene function prediction. Mol. Cell. Proteomics *16*, 121–134.

Wickramasinghe, V.O., and Laskey, R.A. (2015). Control of mammalian gene expression by selective mRNA export. Nat. Rev. Mol. Cell Biol. *16*, 431–442.

Wilkerson, M.D., and Hayes, D.N. (2010). ConsensusClusterPlus: a class discovery tool with confidence assessments and item tracking. Bioinformatics *26*, 1572–1573.

Zhang, B., Wang, J., Wang, X., Zhu, J., Liu, Q., Shi, Z., Chambers, M.C., Zimmerman, L.J., Shaddox, K.F., Kim, S., et al.; NCI CPTAC (2014). Proteogenomic characterization of human colon and rectal cancer. Nature *513*, 382–387.

Zhang, H., Liu, T., Zhang, Z., Payne, S.H., Zhang, B., McDermott, J.E., Zhou, J.Y., Petyuk, V.A., Chen, L., Ray, D., et al.; CPTAC investigators (2016). Integrated proteogenomic characterization of human high-grade serous ovarian cancer. Cell *166*, 755–765.

Cell Reports, Volume *20*

Supplemental Information

Genomic Determinants of Protein Abundance Variation in Colorectal Cancer Cells

Theodoros I. Roumeliotis, Steven P. Williams, Emanuel Gonçalves, Clara Alsinet, Martin Del Castillo Velasco-Herrera, Nanne Aben, Fatemeh Zamanzad Ghavidel, Magali Michaut, Michael Schubert, Stacey Price, James C. Wright, Lu Yu, Mi Yang, Rodrigo Dienstmann, Justin Guinney, Pedro Beltrao, Alvis Brazma, Mercedes Pardo, Oliver Stegle, David J. Adams, Lodewyk Wessels, Julio Saez-Rodriguez, Ultan McDermott, and Jyoti S. Choudhary

Supplemental Figures

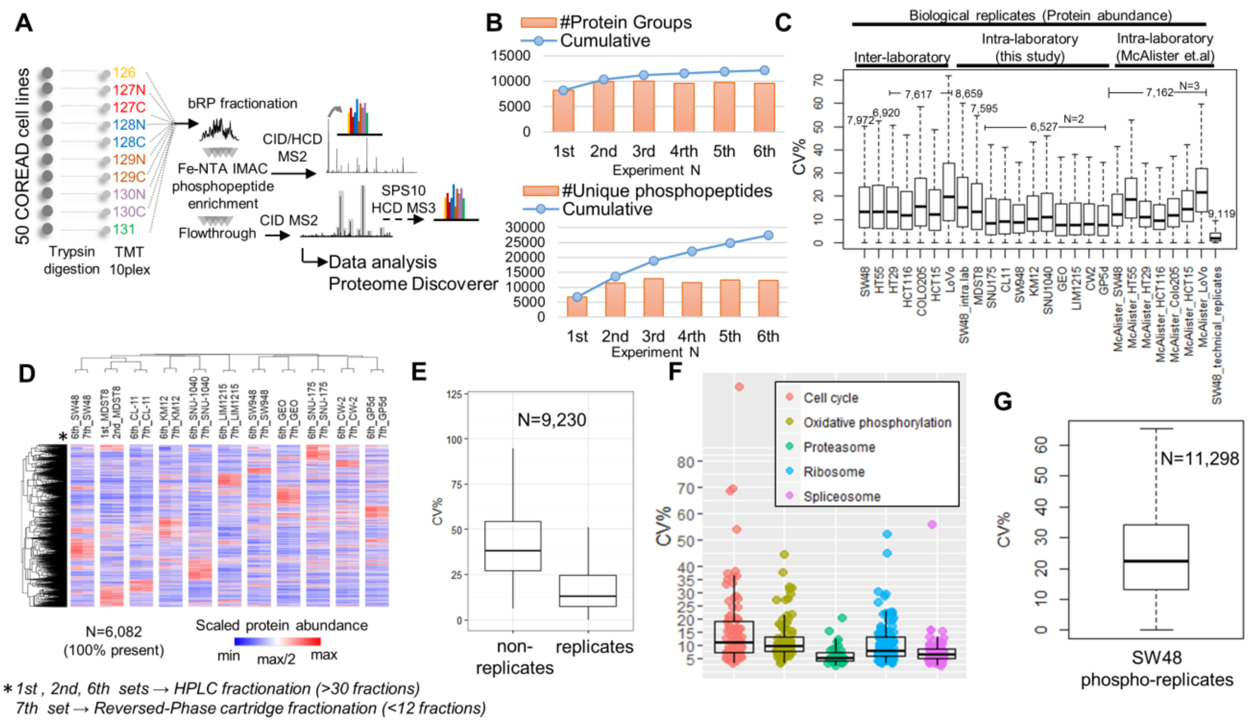

Figure S1. Proteome and phosphoproteome coverage and reproducibility Related to Figure 1. A) Workflow for quantitative global proteome and phosphoproteome analysis. 50 colorectal cancer cell lines (COREAD) were analysed using TMT-10plex in seven multiplex sets. The SW48 cell line was included in each set. Biological replicates of the MDST8 cell line were included in two different sets and the 7th set corresponds to a biological replicate of the 6th set. A technical replicate of the SW48 cell line was included in the 5th set. These were used to evaluate the normalization and the batch effect correction methods. B) Number of protein groups (top panel) and unique phosphopeptides (bottom panel) identified per multiplex set are depicted as orange bars and cumulative numbers are shown as blue lines. C) Boxplots summarizing the coefficient of variation (CV%) of protein abundance measurements for intra-laboratory and inter-laboratory comparisons using biological and technical replicates. D) Heatmap of relative protein abundances between biological replicates for 11 cell lines without missing values. E) Boxplots illustrating the CV% of protein abundance measurements between replicate and non-replicate (all different cell lines) samples across a panel of 11 cell lines. F) Boxplots summarizing the CV% of protein abundance measurements for selected KEGG pathways. G) Boxplot illustrating the CV% of protein phosphorylation measurements for the SW48 biological replicates.

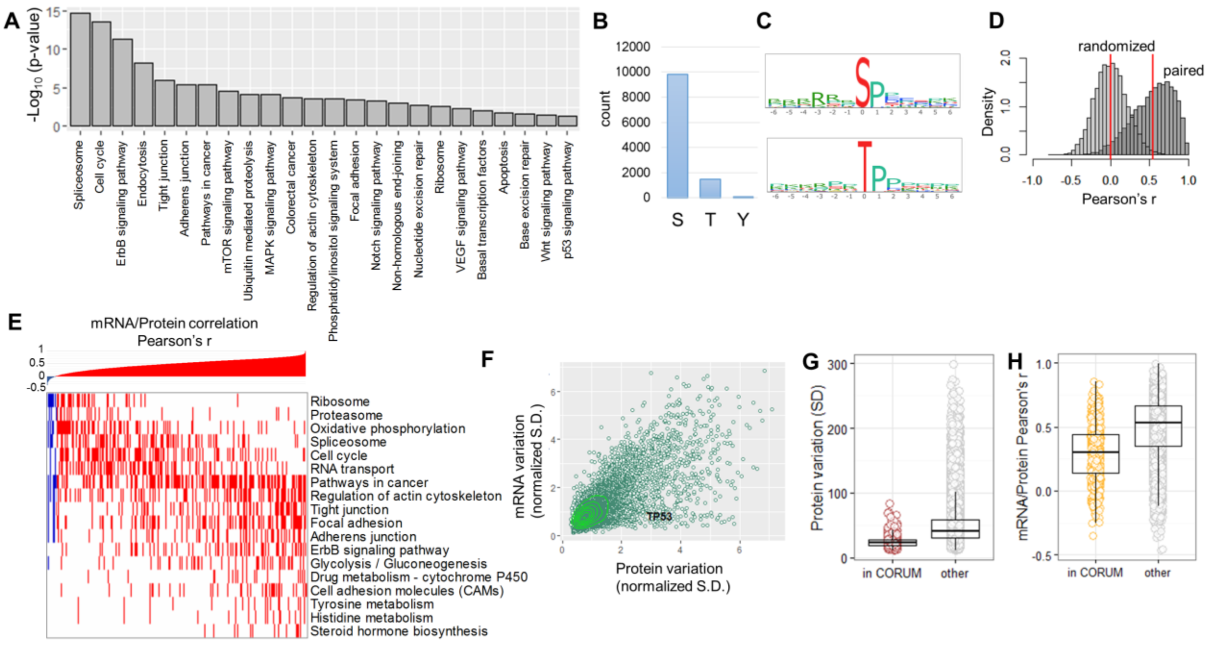

Figure S2. **Qualitative and quantitative characteristics of the phosphoproteome and global mRNA-to-protein comparisons Related to Figures 1 and 6.** A) Enriched KEGG pathways by DAVID analysis of all quantified phosphoproteins. B) Number of S,T and Y phosphorylated residues quantified. C) Logos of the phosphorylation motifs identified. D) The distributions of Pearson coefficients for randomized and matched pairs of phosphopeptide abundances versus protein abundances. E) Gene-level mRNA-to-protein Pearson correlations ranked by lowest to highest value and KEGG pathway enrichment for low and high correlations. F) Scatter plot of mRNA versus protein variation using normalized standard deviation values across the COREAD cell lines. G) Boxplots for protein abundance variation of proteins with high correlations within CORUM complexes versus all other proteins. H) Boxplots for mRNA/protein correlation of proteins with high correlations within CORUM complexes versus all other proteins.

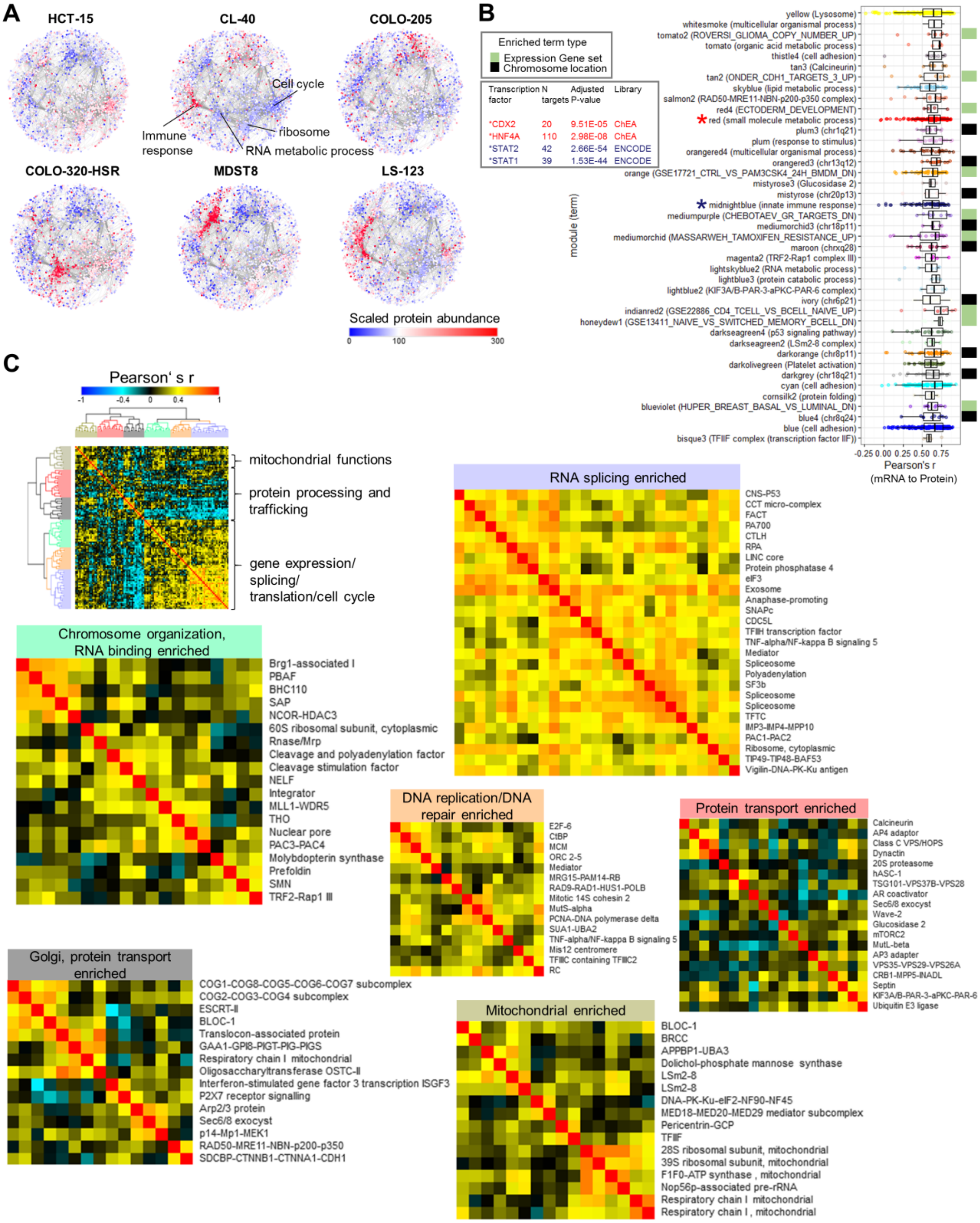
A
HCT-15
CL-40
COLO-205
Cell cycle
Immune response
ribosome
RNA metabolic process
COLO-320-HSR
MDST8
LS-123
Scaled protein abundance
0
100
300
B
Enriched term type
Expression Gene set
Chromosome location
Transcription factor
N targets
Adjusted P-value
Library
*CDX2 20 9.51E-05 ChEA
*HNF4A 110 2.98E-08 ChEA
*STAT2 42 2.66E-54 ENCODE
*STAT1 39 1.53E-44 ENCODE
module (term)
yellow (Lysosome)
whitesmoke (multicellular organismal process)
tomato2 (ROVERSI_GLIOMA_COPY_NUMBER_UP)
tomato (organic acid metabolic process)
thistle4 (cell adhesion)
tan3 (Calcineurin)
tan2 (ONDER_CDH1_TARGETS_3_UP)
skyblue (lipid metabolic process)
salmon2 (RAD50-MRE11-NBN-p200-p350 complex)
red4 (ECTODERM_DEVELOPMENT)
red (small molecule metabolic process)
plum3 (chr1q21)
plum (response to stimulus)
orangered4 (multicellular organismal process)
orangered3 (chr13q12)
orange (GSE17721_CTRL_VS_PAM3CSK4_24H_BMDM_DN)
mistyrose3 (Glucosidase 2)
mistyrose (chr20p13)
midnightblue (innate immune response)
mediumpurple (CHEBOTAEV_GR_TARGETS_DN)
mediumorchid3 (chr18p11)
mediumorchid (MASSARWEH_TAMOXIFEN_RESISTANCE_UP)
maroon (chrxq28)
magenta2 (TRF2-Rap1 complex III)
lightskyblue2 (RNA metabolic process)
lightblue3 (protein catabolic process)
lightblue2 (KIF3A/B-PAR-3-aPKC-PAR-6 complex)
ivory (chr6p21)
indianred2 (GSE22886_CD4_TCELL_VS_BCELL_NAIVE_UP)
honeydew1 (GSE13411_NAIVE_VS_SWITCHED_MEMORY_BCELL_DN)
darkseagreen4 (p53 signaling pathway)
darkseagreen2 (LSm2-8 complex)
darkorange (chr8p11)
darkolivegreen (Platelet activation)
darkgrey (chr18q21)
cyan (cell adhesion)
cornsilk2 (protein folding)
blueviolet (HUPER_BREAST_BASAL_VS_LUMINAL_DN)
blue4 (chr8q24)
blue (cell adhesion)
bisque3 (TFIIF complex (transcription factor IIF))
-0.25 0.00 0.25 0.50 0.75
Pearson's r (mRNA to Protein)
C
Pearson's r
-1 -0.4 0 0.4 1
mitochondrial functions
protein processing and trafficking
gene expression/ splicing/ translation/cell cycle
RNA splicing enriched
CNS-P53
CCT micro-complex
FACT
PA700
CTLH
RPA
LINC core
Protein phosphatase 4
eIF3
Exosome
Anaphase-promoting
SNAPc
CDC5L
TFIIH transcription factor
TNF-alpha/NF-kappa B signaling 5
Mediator
Spliceosome
Polyadenylation
SF3b
Spliceosome
Spliceosome
TFTC
IMP3-IMP4-MPP10
PAC1-PAC2
Ribosome, cytoplasmic
TIP49-TIP48-BAF53
Vigilin-DNA-PK-Ku antigen
Chromosome organization, RNA binding enriched
Brg1-associated I
PBAF
BHC110
SAP
NCOR-HDAC3
60S ribosomal subunit, cytoplasmic
Rnase/Mrp
Cleavage and polyadenylation factor
Cleavage stimulation factor
NELF
Integrator
MLL1-WDR5
THO
Nuclear pore
PAC3-PAC4
Molybdopterin synthase
Prefoldin
SMN
TRF2-Rap1 III
DNA replication/DNA repair enriched
E2F-6
CtBP
MCM
ORC 2-5
Mediator
MRG15-PAM14-RB
RAD9-RAD1-HUS1-POLB
Mitotic 14S cohesin 2
MutS-alpha
PCNA-DNA polymerase delta
SUA1-UBA2
TNF-alpha/NF-kappa B signaling 5
Mis12 centromere
TFIIIC containing TFIIIC2
RC
Protein transport enriched
Calcineurin
AP4 adaptor
Class C VPS/HOPS
Dynactin
20S proteasome
hASC-1
TSG101-VPS37B-VPS28
AR coactivator
Sec6/8 exocyst
Wave-2
Glucosidase 2
mTORC2
MutL-beta
AP3 adaptor
VPS35-VPS29-VPS26A
CRB1-MPP5-INADL
Septin
KIF3A/B-PAR-3-aPKC-PAR-6
Ubiquitin E3 ligase
Golgi, protein transport enriched
COG1-COG8-COG5-COG6-COG7 subcomplex
COG2-COG3-COG4 subcomplex
ESCRT-II
BLOC-1
Translocon-associated protein
GAA1-GPI8-PIGT-PIG-PIGS
Respiratory chain I mitochondrial
Oligosaccharyltransferase OSTC-II
Interferon-stimulated gene factor 3 transcription ISGF3
P2X7 receptor signalling
Arp2/3 protein
Sec6/8 exocyst
p14-Mp1-MEK1
RAD50-MRE11-NBN-p200-p350
SDCBP-CTNNB1-CTNNA1-CDH1
Mitochondrial enriched
BLOC-1
BRCC
APPBP1-UBA3
Dolichol-phosphate mannose synthase
LSm2-8
LSm2-8
DNA-PK-Ku-eIF2-NF90-NF45
MED18-MED20-MED29 mediator subcomplex
Pericentrin-GCP
TFIIF
28S ribosomal subunit, mitochondrial
39S ribosomal subunit, mitochondrial
F1F0-ATP synthase , mitochondrial
Nop56p-associated pre-rRNA
Respiratory chain I mitochondrial
Respiratory chain I , mitochondrial

Figure S3. The colorectal cancer cell protein correlation network Related to Figure 1. A) Examples of unique network signatures for six COREAD cell lines. B) Boxplots of Pearson mRNA-to-protein correlation for modules highly corresponding to mRNA levels. Dots are color-coded according to the default WGCNA module name. Modules enriched for GSEA gene sets and chromosome locations are highlighted with green and black marks respectively. Enriched transcription factors in the “small molecule metabolic process” and “innate immune response” modules are displayed. C) Correlation heatmap of protein complexes based on the similarities of their representative profiles (eigengenes). The heatmap is divided in six main clusters which are color-coded and magnified. Shortened CORUM complex names are used. Duplicate entries represent protein complexes that are separated into more than one modules.

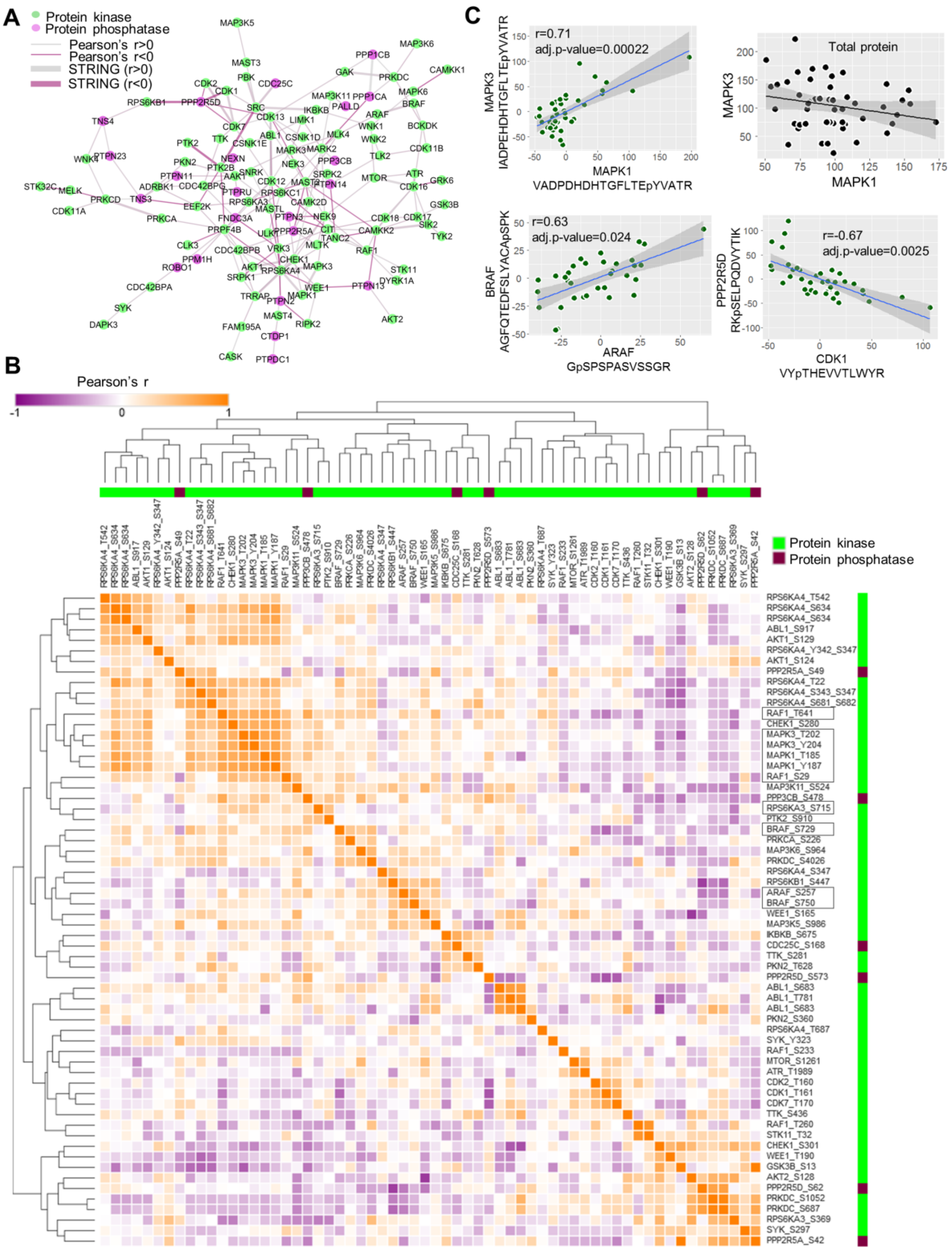
A
Protein kinase
Protein phosphatase
Pearson's r>0
Pearson's r<0
STRING (r>0)
STRING (r<0)
C
r=0.71
adj.p-value=0.00022
MAPK3
IADPEHDHTGFLTEpYVATR
MAPK1
VADPDHDHTGFLTEpYVATR
Total protein
MAPK3
MAPK1
r=0.63
adj.p-value=0.024
BRAF
AGFQTEDFSLYACApSPK
ARAF
GpSPSPASVSSGR
r=-0.67
adj.p-value=0.0025
PPP2R5D
RKpSELPQDVYTIK
CDK1
VYpTHEVVTLWYR
B
Pearson's r
-1
0
1
Protein kinase
Protein phosphatase
RPS6KA4_T542
RPS6KA4_S634
RPS6KA4_S634
ABL1_S917
AKT1_S129
RPS6KA4_Y342_S347
AKT1_S124
PPP2R5A_S49
RPS6KA4_T22
RPS6KA4_S343_S347
RPS6KA4_S681_S682
RAF1_T641
CHEK1_S280
MAPK3_T202
MAPK3_Y204
MAPK1_T185
MAPK1_Y187
RAF1_S29
MAP3K11_S524
PPP3CB_S478
RPS6KA3_S715
PTK2_S910
BRAF_S729
PRKCA_S226
MAP3K6_S964
PRKDC_S4026
RPS6KA4_S347
RPS6KB1_S447
ARAF_S257
BRAF_S750
WEE1_S165
MAP3K5_S986
IKBKB_S675
CDC25C_S168
TTK_S281
PKN2_T628
PPP2R5D_S573
ABL1_S683
ABL1_T781
ABL1_S683
PKN2_S360
RPS6KA4_T687
SYK_Y323
RAF1_S233
MTOR_S1261
ATR_T1989
CDK2_T160
CDK1_T161
CDK7_T170
TTK_S436
RAF1_T260
STK11_T32
CHEK1_S301
WEE1_T190
GSK3B_S13
AKT2_S128
PPP2R5D_S62
PRKDC_S1052
PRKDC_S687
RPS6KA3_S369
SYK_S297
PPP2R5A_S42

Figure S4. ***De novo* prediction of a phosphorylation network involving kinases and phosphatases Related to Figure 6.** A) Correlation network of positive and negative associations (Benj. Hoch. FDR<0.1) among phosphopeptides belonging to protein kinases and phosphatases. The nodes represent the phosphorylated kinases or phosphatases. B) Correlation heatmap of phosphopeptides belonging to kinases and phosphatases involved in KEGG signalling pathways. Duplicate entries represent protein phosphorylation sites that were identified by different overlapping phosphopeptides with different lengths. Phosphoproteins of the MAPK pathway are outlined. C) Correlation plots of MAPK1 and MAPK3 phosphorylation (top left) and total protein (top right). Correlation plots of BRAF and ARAF phosphorylation (bottom left) and negative correlation plot between phosphorylated CDK1 kinase and PPP2R5D phosphatase (bottom right).

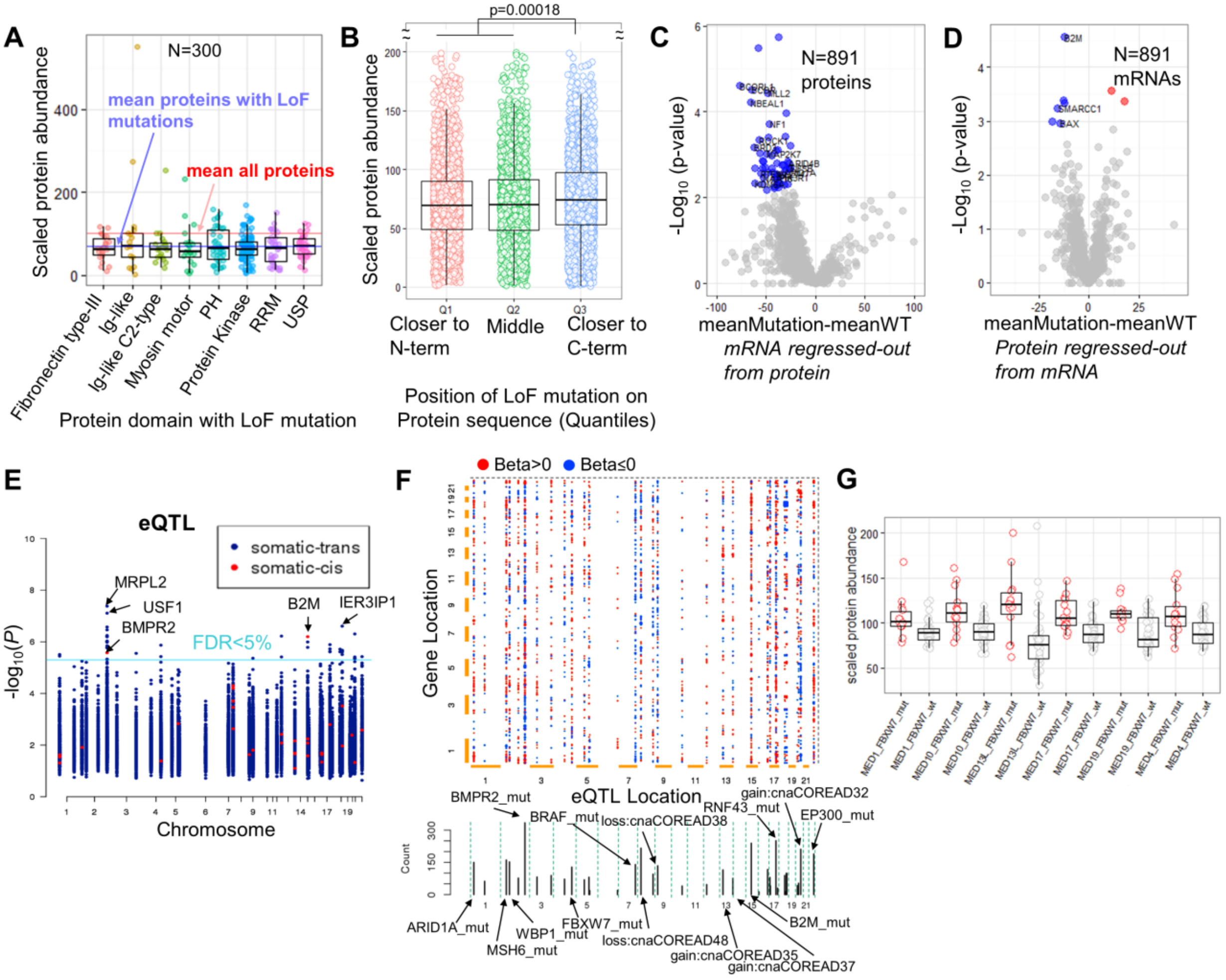

Figure S5. The impact of mutations on protein and mRNA abundances Related to Figures 2,3,4 and 5. A) Boxplots of scaled protein abundances for proteins with LoF mutations within specific protein domains. B) Boxplots of scaled protein abundances for proteins with LoF mutations located closer to the N-terminus, in the middle and closer to the C-terminus of the protein sequence. C) Volcano plot summarizing the effect of LoF mutations on the respective mRNA regressed-out protein levels (ANOVA test). Hits at permutation-based FDR<0.1 are coloured. D) Volcano plot summarizing the effect of LoF mutations on the respective protein regressed-out mRNA levels (ANOVA test). Hits at permutation-based FDR<0.1 are coloured. E) Identification of *cis* and *trans* eQTLs in colorectal cancer cell lines considering cancer driver variants. The p-value and genomic coordinates for the most confident non-redundant mRNA-variant association tests are depicted in the Manhattan plot. F) Representation of eQTLs as 2D plot of variants (x-axis) and associated genes (y-axis). Associations with q<0.3 are shown as dots coloured by the beta value (red: positive association, blue: negative association) while the size is increasing with the

confidence of the association. Cumulative of the number of associations per variant is plotted below the 2D matrix. G) Boxplots of protein abundance of proteins from the Mediator complex identified only by pQTL analysis, in cell lines with mutated (red) and wt (grey) *FBXW7*.

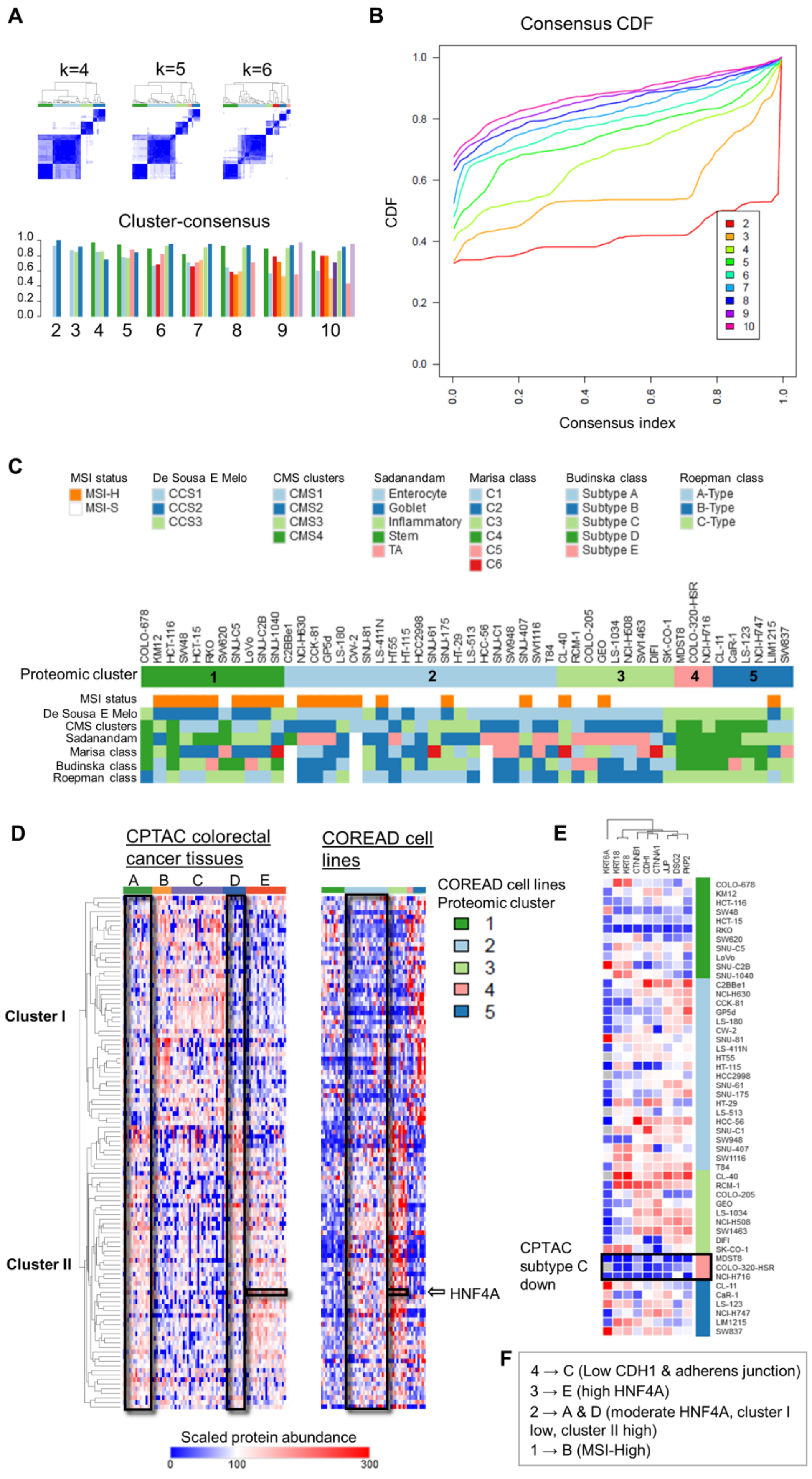

A
k=4
k=5
k=6
Cluster-consensus
1.0
0.8
0.6
0.4
0.2
0.0
2 3 4 5 6 7 8 9 10
B
Consensus CDF
CDF
Consensus index
2 3 4 5 6 7 8 9 10
C
MSI status
MSI-H
MSI-S
De Sousa E Melo
CCS1
CCS2
CCS3
CMS clusters
CMS1
CMS2
CMS3
CMS4
Sadanandam
Enterocyte
Goblet
Inflammatory
Stem
TA
Marisa class
C1
C2
C3
C4
C5
C6
Budinska class
Subtype A
Subtype B
Subtype C
Subtype D
Subtype E
Roepman class
A-Type
B-Type
C-Type
COLO-678 KM12 HCT-116 SW48 HCT-15 RKO SW620 SNU-C5 LoVo SNU-C2B SNU-1040 C2BBe1 NCI-H630 CCK-81 GP5d LS-180 CW-2 SNU-81 LS-411N HT55 HT-115 HCC2998 SNU-61 SNU-175 HT-29 LS-513 HCC-56 SNU-C1 SW948 SNU-407 SW1116 T84 CL-40 RCM-1 COLO-205 GEO LS-1034 NCI-H508 SW1463 DIFI SK-CO-1 MDST8 COLO-320-HSR NCI-H716 CL-11 CaR-1 LS-123 NCI-H747 LIM1215 SW837
Proteomic cluster
1 2 3 4 5
MSI status
De Sousa E Melo
CMS clusters
Sadanandam
Marisa class
Budinska class
Roepman class
D
CPTAC colorectal cancer tissues
A B C D E
COREAD cell lines
Cluster I
Cluster II
HNF4A
COREAD cell lines Proteomic cluster
1
2
3
4
5
Scaled protein abundance
0
100
300
E
KRT6A KRT18 KRT8 CTNNB1 CDH1 CTNNA1 JUP DSG2 PKP2
CPTAC subtype C down
F
4 → C (Low CDH1 & adherens junction)
3 → E (high HNF4A)
2 → A & D (moderate HNF4A, cluster I low, cluster II high)
1 → B (MSI-High)

Figure S6. Consensus clustering of colorectal cancer cell lines and overlap with colorectal cancer tissue subtypes Related to Figure 6. A) Proteome clusters were derived based on consensus clustering (ConsensusClusterPlus R package) using the 30% most variable proteins with no missing values. The consensus matrices for target values *k*=4,5 and 6 are visualized (top panel) along with the cluster-consensus plot displaying the mean of all pairwise consensus values between a cluster's members at each *k* (bottom panel). Balanced mean consensus values are obtained at *k*=5. B) The empirical cumulative distribution function (CDF) plot which indicates the *k* at which the distribution reaches an approximate maximum. C) Overlap of the proteomics cell line subtypes with tissue level classifications. D) Heatmaps using the cell line signature proteins that are also differentially regulated between the CPTAC colorectal cancer proteomic subtypes. Scaled protein abundances are used for both datasets. Proteins (rows) are hierarchically clustered based on the CPTAC data and columns are sorted by CPTAC proteomics subtype (left panel) or by the COREAD cell lines subtypes (right panel). E) The abundance profiling of CPTAC subtype C down-regulated proteins across the COREAD cell lines panel. Strong down-regulation is observed in COREAD cell lines subtype 4. F) Summary of the overlap between the CPTAC colorectal cancer tissue proteome subtypes and the COREAD cell lines proteomic subtypes.

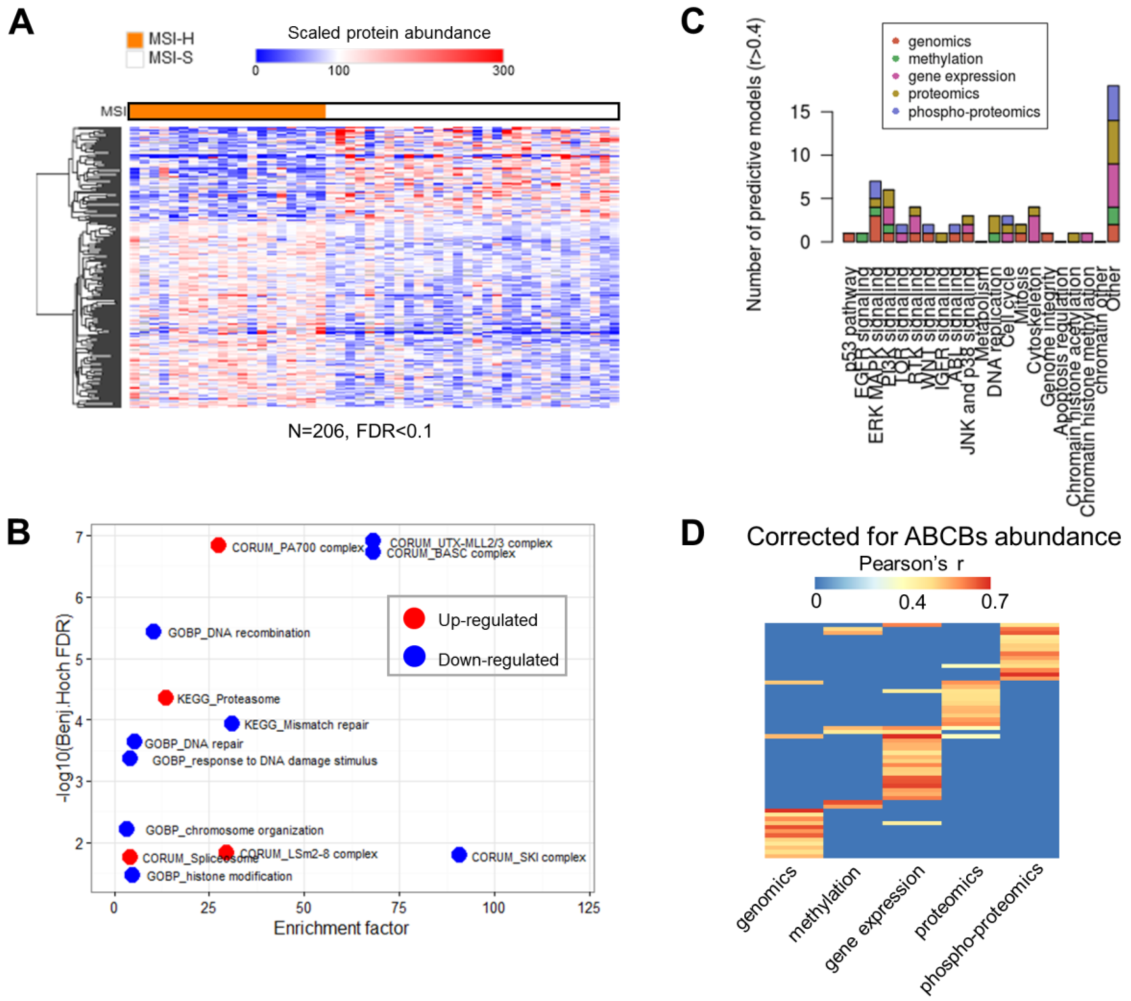

Figure S7. Proteins associated with microsatellite instability and pharmacoproteomic models Related to Figures 6 and 7. A) Heatmap of MSI-high associated proteins (Welch t-test, permutation-based FDR<0.1). Rows represent proteins and columns correspond to cell lines grouped according to the MSI status. B) Scatter plot of fold-enrichment versus significance FDR for enriched biological processes, KEGG pathways and CORUM complexes in the MSI-high associated proteins. C) The number of drugs where response was specifically predicted by one

molecular data type, stratified by each of the four molecular data types and by the 21 drug classes as defined by Iorio et al. (2016). D) Heatmap indicating for each drug and each data type whether a predictive model could be fitted using ABCBs-corrected drug response data.

Supplemental tables titles and legends

Table S1 Related to Figures 1,2,3,5,6 and 7. Relative protein abundances in the COREAD cell lines. Scaled protein quantification values for 50 colorectal cancer cell lines. Proteins affected by mutations or DNA copy number alterations are annotated by ANOVA p-value and average log2fold-changes where applicable.

Table S2 Related to Figures 6, S2 and S4. Relative phosphopeptide abundances in the COREAD cell lines. Scaled phosphopeptide quantification values for 50 colorectal cancer cell lines. Phosphopeptides are annotated by the known regulatory kinase and by KEGG pathway where applicable. The values are not corrected or normalized for total protein levels.

Table S3 Related to Figure 1. Correlation of proteins belonging to known CORUM complexes. Overlap of known CORUM protein complexes with the COREAD cell lines proteome. The mean and median correlation between protein complex components is shown for all complexes. Proteins with outlier profiles (high or low correlations) are labelled.

Table S4 Related to Figure 1. Annotation of WGCNA modules. Enrichment of Gene Ontology, GSEA, KEGG, CORUM and Pfam terms for the WGCNA modules. Significance FDR is shown for each term.

Table S5 Related to Figure 1. The full WGCNA network. The entire WGCNA correlation network with weights greater than 0.02. The respective Pearson's correlations are also shown and CORUM interactions are highlighted (value=1). Modules that are possibly driven by DNA copy number variations are highlighted (value=1).

Table S6 Related to Figure 4. Proteomics and RNA-seq results for the ARID1A, ARID2 and PBRM1 CRISPR-cas9 experiments. Absolute and scaled S/N TMT values are shown for the proteomics data. ANOVA p-values refer to any comparison between the WT, ARID1A, ARID2 and PBRM1 replicate groups. RNA-seq quantification and significance is shown only for the respective identified proteins.

Table S7 Related to Figure 7 and Figure S7. Associations between drug response and molecular data. The predictive performance, data type and target pathway is shown for each association. The column "Corrected for ABCBs?" indicates whether the feature was predicted with the mean protein abundance of ABCB1 and ABCB11 regressed-out from drug response data.

Supplemental Experimental Procedures

Colorectal cancer cell lines culture and reagents

Cells were grown in either DMEM/F12 medium (Gibco) supplemented with 10% fetal calf serum (v/v) (Gibco) and 50 U/mL penicillin, 50 mg/mL streptavidin (Gibco), or RPMI 1640 medium (Gibco) supplemented with 10% fetal calf serum (v/v) (Gibco), 50 U/mL penicillin, 50 mg/mL streptavidin (Gibco), 2.5 mg/mL glucose (Sigma-Aldrich) and 1 mM sodium pyruvate (Gibco), and maintained at 37 °C in a humidified atmosphere at 5% CO_2. Cells were harvested by incubating with TrypLE (Gibco) until detached, and washing twice with cold PBS solution before snap freezing on dry ice.

Viability assays

Cells were seeded at 5,000 cells per well in 96-well tissue culture plates in growth media. The following day, docetaxel (Selleckchem) was added to give a final concentration range of 0.2 nM – 50 nM, with triplicate wells of each condition. Assay was carried out in the presence or absence of 2.5 μM tariquidar (Selleckchem). Control wells received an equivalent volume of DMSO. Assay plates were incubated at 37°C, 5% CO_2 for 6 days, before adding

CellTiter Blue (Promega) as an indicator of cell viability. Fluorescent signal was measured after 6 h using a Molecular Devices Paradigm plate reader (560/590 nm filter).

iPSC lines culture

Human induced pluripotent stem cells have been maintained in mTeSR E8 media (StemCell Technologies) on Synthemax II-SC Substrate-treated plates (Corning). All lines have been passaged as single cells by incubating with Accutase for 10 min at 37 °C, scraping with cell lifters and re-suspending 5 to 10 times with a 10 mL pipette. Cells were then plated in mTeSR-E8 media containing 10 uM ROCK inhibitor (Y-27632 dihydrochloride monohydrate, StemCell Technologies). When passaging as clumps, cells were incubated 3 minutes in Gentle Dissociation Buffer, scraped with cell lifters, resuspended once or twice with a 10 mL pipette and plated in mTeSR-E8 media.

iPSC lines CRISPR targeting

Production of tumor suppressor gene knock out lines was achieved through the substitution of an asymmetrical exon by a Puromycin cassette and introduction of a frame-shift indel in the remaining allele. A hSpCas9 and two small guide RNA expression vectors along with a template vector were used. The template vector harboured an EF1a-Puromycin cassette with two flanking 1.5 kb homology arms designed around the asymmetric exon of interest. For each knock out line, 2×10^6 single cells were transfected using the Amaxa Human Stem Cell Nucleofector® Kit 2 (Lonza) with 4 ug, 3 ug and 2 ug of each plasmid, respectively, and plated in 10 cm plates. After 72 h, cells were selected in 3 ug/mL Puromycin and colonies expanded and genotyped for the presence of a frame-shift indel by Sanger sequencing. For the proteomic analysis 2×10^5 single cells were plated in 10 cm plates and collected once colonies showed the typical dense sharp-edged morphology. Cells were washed in DPBS, scraped with cell lifters and cell pellets were centrifuged at 2,500 rpm for 5 min at 4 °C. Pellets were then snap frozen in dry ice and stored at -80 °C.

Protein digestion and TMT labelling

The PBS washed cell pellets containing $2{\sim}3\times10^6$ cells were dissolved in 150 µL 0.1 M triethylammonium bicarbonate (TEAB), 0.1% SDS with pulsed probe sonication (EpiShear™, power 40%) on ice for 20 sec and direct boiling at 95 °C in a preheated heat block for 10 min. The sonication-boiling procedure was performed twice and cellular debris was removed by centrifugation at 12,000 rpm for 10 min. Protein concentration was measured with Quick Start Bradford Protein Assay (Bio-Rad) according to manufacturer's instructions. Aliquots containing 100 µg of total protein were prepared for trypsin digestion. Cysteine disulfide bonds were reduced with a final concentration of 5 mM tris-2-carboxymethyl phosphine (TCEP) followed by 1 h incubation in heating block at 60 °C. Cysteine residues were blocked with a final concentration of 10 mM freshly prepared Iodoacetamide (IAA) solution and 30 min incubation at room temperature in dark. Trypsin (Pierce, MS grade) was added at mass ratio 1:30 for overnight digestion. The resultant peptides were diluted up to 100 µL with 0.1 M TEAB buffer. A 41 µL volume of anhydrous acetonitrile was added to each TMT 10-plex reagent (Thermo Scientific) vial and after vortex mixing the content of each TMT vial was transferred to each sample tube. The labelling reaction was quenched after 1 hour by the addition of 8 µL 5% hydroxylamine. Samples were combined and the mixture was dried with speedvac concentrator and stored at -20 °C until the high-pH Reverse Phase (RP) fractionation.

Peptide fractionation

High pH Reverse Phase (RP) peptide fractionation was performed with the Waters, XBridge C18 column (2.1 x 150 mm, 3.5 µm, 120 Å) on a Dionex Ultimate 3000 HPLC system equipped with autosampler. Mobile phase (A) was composed of 0.1% ammonium hydroxide and mobile phase (B) was composed of 100% acetonitrile, 0.1% ammonium hydroxide. The TMT labelled peptide mixture was reconstituted in 100 µL mobile phase (A), centrifuged and injected for fractionation. The multi-step gradient elution method at 0.2 mL/min was as follows: for 5 minutes isocratic at 5% (B), for 35 min gradient to 35% (B), for 5 min gradient to 80% (B), isocratic for 5 minutes and re-equilibration to 5% (B). Signal was recorded at 215 and 280 nm and fractions were collected in a time dependent manner every 30 sec. The collected fractions were dried with SpeedVac concentrator and stored at -20 °C until the LC-MS analysis. For the replication sample set (7th set) and the CRISPR/cas9 proteomic experiments, peptide fractionation was performed on reversed-phase OASIS HLB cartridges at high pH and up to 12 fractions were collected for each set.

Phosphopeptide enrichment

The peptide fractions were reconstituted in 10 uL of 20% isopropanol, 0.5% formic acid binding solution and were loaded on 10 uL of phosphopeptide enrichment IMAC resin (PHOS-Select™ Iron Affinity Gel) already washed and

conditioned with binding solution in custom made filter tips fitted on the eppendorf tubes caps. The resin was washed three times with 40 uL of binding solution and centrifugation at 300 g after 2 h of binding and the flow-through solutions were collected. Phosphopeptides were eluted three times with 70 uL of 40% acetonitrile, 400 mM ammonium hydroxide solution. Both the eluents and flow-through solutions were dried in a speedvac and stored at -20 °C until the phosphoproteomic and proteomic LC-MS analysis respectively.

LC-MS analysis

LC-MS analysis was performed on the Dionex Ultimate 3000 UHPLC system coupled with the Orbitrap Fusion Tribrid Mass Spectrometer (Thermo Scientific). Each peptide fraction was reconstituted in 40 μL 0.1% formic acid and a volume of 7 μL was loaded to the Acclaim PepMap 100, 100 μm × 2 cm C18, 5 μm, 100 Å trapping column with the μlPickUp mode at 10 μL/min flow rate. The sample was then subjected to a multi-step gradient elution on the Acclaim PepMap RSLC (75 μm × 50 cm, 2 μm, 100 Å) C18 capillary column retrofitted to an electrospray emitter (New Objective, FS360-20-10-D-20) at 45 °C. Mobile phase (A) was composed of 0.1% formic acid and mobile phase (B) was composed of 80% acetonitrile, 0.1% formic acid. The gradient separation method at flow rate 300 nL/min was as follows: for 95 min gradient to 42% B, for 5 min up to 95% B, for 8 min isocratic at 95% B, re-equilibration to 5% B in 2 min, for 10 min isocratic at 5% B.
Precursors were selected with mass resolution of 120k, AGC 3×10^5 and IT 100 ms in the top speed mode within 3 sec and were isolated for CID fragmentation with quadrupole isolation width 0.7 Th. Collision energy was set at 35% with AGC 1×10^4 and IT 35 ms. MS3 quantification spectra were acquired with further HCD fragmentation of the top 10 most abundant CID fragments isolated with Synchronous Precursor Selection (SPS) excluding neutral losses of maximum m/z 30. Iontrap isolation width was set at 0.5 Th, collision energy was applied at 45% and the AGC setting was at 6×10^4 with 100 ms IT. The HCD MS3 spectra were acquired within 120-140 m/z with 60k resolution. Targeted precursors were dynamically excluded for further isolation and activation for 45 seconds with 7 ppm mass tolerance. Phosphopeptide samples were analyzed with a CID-HCD method at the MS2 level. MS level AGC was set at 5×10^5, IT was set at 150 ms and exclusion duration at 30sec. AGC settings for CID and HCD fragmentation were 5×10^4 and 2×10^5 respectively. The fractions for the replication and CRISPR/cas9 sets were analysed with 180 min and 300 min LC-MS runs respectively and the analysis was repeated by setting upper intensity thresholds between 2-5×10^6 to capture lower abundant peptides. The total data collection was accomplished with 469 LC-MS runs in about 1,000 hours of analysis.

Protein identification and quantification

The acquired mass spectra were submitted to SequestHT search in Proteome Discoverer 1.4 (P.D 2.1 for the CRISPR-cas9 experiments) for protein identification and quantification. The precursor mass tolerance was set at 20 ppm and the fragment ion mass tolerance was set at 0.5 Da for the CID and at 0.02 Da for the HCD spectra used for the phosphopeptide analysis. Spectra were searched for fully tryptic peptides with maximum 2 miss-cleavages and minimum length of 6 amino acids. TMT6plex at N-termimus, K and Carbamidomethyl at C were defined as static modifications. Dynamic modifications included oxidation of M and Deamidation of N,Q. Maximum two different dynamic modifications were allowed for each peptide with maximum two repetitions each. Search for phospho-S,T,Y was included only for the IMAC data. Peptide confidence was estimated with the Percolator node. Peptide FDR was set at 0.01 and validation was based on q-value and decoy database search. All spectra were searched against a UniProt fasta file containing 20,165 reviewed human entries. The Reporter Ion Quantifier node included a custom TMT-10plex Quantification Method with integration window tolerance 15 ppm, integration method the Most Confident Centroid at the MS3 level (or at the MS2 level for the IMAC data) and missing channels were replaced by minimum intensity. Only peptides uniquely belonging to protein groups were used for quantification. Peptide-spectrum matches (PSMs) with mean TMT intensity less than 500 across samples were discarded. The TMT intensities of PSMs uniquely matching to the same protein or phosphopeptide were summed to obtain protein and phosphopeptide level intensities respectively. Protein and phosphopeptide summed intensities were further corrected for equal loading across samples by median normalization (divide by column median). Scaled quantitative values in the range of 0 to 1,000 were obtained by dividing each TMT value with the mean TMT intensity across samples per protein in each multiplex set separately (divide by row mean × 100, per sample set). To detect net phosphorylation changes, the relative protein abundances were regressed out from the respective phosphopeptide levels. Phosphorylation abundances were set as the dependent variables (y) and protein abundances were set as the independent variables (x). The residuals of the y~x linear model were the phosphorylation levels not driven by protein abundance levels.

RNAseq data processing and identification of differentially expressed genes for the CRISPR/cas9 cell lines

Knockout human induced pluripotent stem cells (hiPSC) for *ARID1A*, *ARID2* and *PBRM1* and WT cells were cultured in the same conditions as specified in the protein extraction section. RNA was extracted using the RNAeasy Kit (QiAGEN) from a total of six biological replicates per gene knockout and four WT cells for a total of 22 samples. Independent barcoded stranded libraries were prepared for each sample and pooled into a multiplex library that was sequenced across six lanes using the Illumina HiSeq4000. Raw 75bp paired-end reads were aligned to the human reference genome (GRCh38) using STAR (v2.5.0a) (Dobin et al., 2013) and ENSEMBL (v84) human annotation. Subsequently, uniquely mapped read pairs with a mapping quality >10 were counted using htseq-count (Anders et al., 2015) with the model intersection_nonempty and ENSEMBL v84 annotation. Raw counts were normalised by calculating the transcripts per kilobase per million (TPM) obtained for each gene for each biological replicate. Pearson correlation among the different samples was calculated based on the TPM values of all the protein coding genes. Differentially expressed genes were identified by performing paired comparisons between the three independent groups of targeted hiPS cells (*ARID1A, ARID2 and PBRM1)* against WT hiPS cells (BoB) using DESeq2 (Love et al., 2014) (v1.10.1). Once dispersion estimates and normalised counts were calculated, genes with mean normalized <1, across all samples within the comparison, were filtered out. P-values were re-adjusted using the Benjamini-Hochberg correction for multiple-testing.

Visualization on genome coordinates

The peptides identified in this study were mapped onto the human reference genome GRCh38/hg38 using GENCODE v25 and GRCh37/hg19 using GENCODE v25lift37 (Wright et al., 2016) through the peptide to genome mapping tool PoGo (http://www.sanger.ac.uk/science/tools/pogo). The resulting BED output files were used to create a track-hub (Raney et al., 2014), i.e. web-accessible directory of genomic related data for visualisation of a large number of genome-wide data sets, through application of the TrackHubGenerator tool (http://www.sanger.ac.uk/science/tools/trackhubgenerator). The track-hub is made available through ftp://ngs.sanger.ac.uk/production/proteogenomics/WTSI_proteomics_COREAD and can be loaded in prominent online genome browsers. The Hub can be loaded in the Ensembl genome browser (Aken et al., 2017) through selection of 'Custom tracks' and copying the following URL into the 'Data' field and selection of 'Track Hub' as data format: http://ngs.sanger.ac.uk/production/proteogenomics/WTSI_proteomics_COREAD/hub.txt. After adding the data and closing the configuration panel five new tracks will appear in the "Region in detail" view showing the overall identified peptides, and phosphopeptides. The tracks can also be loaded in the UCSC genome browser (Kent et al., 2002) through 'My Data' and the 'My Hubs'. After copying the above link and adding the hub the tracks will appear in the browser after selection of the reference assembly hg19 or hg38.

To support other types of visualisation the additional folder 'suppl' in the track-hub directory provides downloadable GTF mappings of the peptides with associated gene names and gene biotypes for reference assemblies hg19 and hg38. Furthermore, comparative visualisation of peptide quantitation across all 50 COREAD cell lines for both reference assemblies are provided through GCT files in the respective assembly folders. These files can be visualised in the Integrative Genomics Viewer (IGV) (Robinson et al., 2011). After selection of the genome assembly the respective files can be loaded through the file selection dialog. Comparative visualisation on protein level in association with chromosome bands is provided separately in the file 'WTSI_proteomics_COREAD_50_proteome_bands.gct'.

COREAD gene expression data

The mRNA relative abundances between 45 colorectal cancer cell lines were computed from publicly available microarray gene expression data (ArrayExpress, accession: E-MTAB-3610) using robust Multi-Array Average (RMA) (Bolstad et al., 2003). Scaled values were obtained by row-mean normalization as applied to the proteomics data.

Weighted correlation network analysis

Weighted correlation network analysis was performed with the WGCNA package in RStudio using 8,295 proteins quantified in at least 80% of the cell lines. A soft threshold at power 7 was selected based on scale free topology model fit. Other parameters included: mergeCutHeight = 0.25 and minModuleSize = 3. Gene Ontology annotation of the modules was performed by the WGCNA package and enrichment for additional terms was performed with Fisher's test using GSEA, GOBP-slim, CORUM, KEGG, and Pfam terms in Perseus software (Tyanova et al., 2016).

Consensus clustering of the cell lines

Unsupervised clustering of the cell lines was performed with the ConsensusClusterPlus R package using the top 30% most variable proteins without missing values (N=2,161). Proteome clusters were derived based on k-means clustering and 1,000 resampling repetitions in the range of 2 to 10 clusters. The consensus matrices for target values k=4, 5 and 6 were visualized along with the empirical cumulative distribution function (CDF) plot which indicates the k at which the distribution reaches an approximate maximum and the cluster-consensus plot displaying the mean of all pairwise consensus values between a cluster's members at each k.

Comparison of COREAD cell lines proteomic subtypes with CPTAC colorectal cancer proteomic subtypes

To assess the overlap between the COREAD and the CPTAC colorectal cancer proteomic subtypes from Zhang et al., we tested whether our COREAD signature proteins (differentially expressed between the five subtypes, ANOVA test, permutation-based FDR<0.05, N=723) were also differentially expressed between the CPTAC subtypes. For the CPTAC data, proteins with mean intensity greater than 1.4 (median label free intensity) across tissues were considered (N=3,615), and the label free quantification values were row-mean scaled similarly to the cell line data. About 75% of the COREAD signature proteins (N=251) that were found in the scaled CPTAC subset were also differentially regulated between the CPTAC subtypes (ANOVA test, permutation-based FDR<0.1). The most variable proteins of the latter subset were visualized with hierarchical clustering and the resultant clusters were compared with the COREAD subtypes profiles.

Statistical tests and visualization

ROC curves were plotted in Python (v 2.7.10) module Seaborn (v 0.7.1) using known STRING and CORUM interactions as true positive hits. Enrichment for biological terms and pathways was performed in Perseus 1.4 software (Tyanova et al., 2016) with Fisher's test or with the 1D-annotation enrichment method (Cox and Mann, 2012). The enrichment score indicates whether the proteins in a given pathway tend to be systematically up-regulated or down-regulated based on Wilcoxon-Mann-Whitney test. The 1D-annotation enrichment method was also applied for the enrichment of KEGG pathways with low and high mRNA-to-protein correlations and for kinase enrichment analysis using known kinase-substrate associations from the PhosphoSitePlus database and non-regressed phosphorylation abundances. All terms were filtered for Benjamini-Hochberg FDR<0.05. Correlation analysis of regressed phosphorylation profiles was performed in RStudio with Benjamini-Hochberg FDR multiple testing corrections. PHOSIDA was used for enrichment of phosphorylation motifs (Gnad et al., 2011). ANOVA and Welch's tests were performed in Perseus 1.4 software. Permutation based FDR correction was applied to the ANOVA test p-values for the assessment of the impact of mutations and copy number variations on protein and mRNA abundances in Perseus 1.4 software. The web-based tool Morpheus (https://software.broadinstitute.org/morpheus/) was used for hierarchical clustering and visualization of heatmaps. Volcano plots, boxplots, distribution plots, scatter plots and bar plots were drawn in RStudio with the ggplot2 and ggrepel packages. KEGG pathway enrichment for the identified phosphoproteins was performed in DAVID (Huang et al., 2009) (https://david.ncifcrf.gov/). PROSITE protein domains (Sigrist et al., 2010) were used to assess the impact of mutated domains on protein abundances. Transcription factor enrichment analysis was performed in the Enrichr tool (http://amp.pharm.mssm.edu/Enrichr/). STRING and Cytoscape 3.2.1 (Shannon et al., 2003) were used for network analysis and visualization.

Identification of pQTL and eQTL

We performed a whole-genome protein level QTL (pQTL) mapping by testing for associations between the protein level measurements and a set of 19 variant genes and 17 CNAs representing colorectal cancer drivers, filtered for at least 5 events across the cell lines. From the original 9,489 proteins measured, we selected proteins expressed across all cell lines and aligned uniquely to one Ensemble gene (N=6,929). Proteins on X, Y and mitochondrial chromosome were excluded. To increase the robustness with respect to possible outlying protein values, all protein measures were quantile normalized to a Gaussian distribution prior to fitting a model. All associations were implemented by LIMIX using a linear regression test. Let the protein level measurements be $\boldsymbol{y}$, $\boldsymbol{x}_s$ corresponds to the binary representation of somatic variants. We can write the linear regression model as:

$$\boldsymbol{y} = \boldsymbol{1}\mu + \boldsymbol{x}_s\beta_s + \boldsymbol{\psi}, \quad \text{where} \quad \boldsymbol{\psi} \sim N(0, \sigma_e^2\boldsymbol{I}).$$

Here β_s denotes the effect size of the tested variant, μ is the intercept and $\boldsymbol{\psi}$ is the residual noise.Similarly for eQTL analysis, genes on X, Y and mitochondrial chromosome were excluded (N=16,546). We used a shared normalization, regression and multiple testing correction for eQTL as pQTL.

Drug response prediction

For the associations with drug response, we used the genomics, methylation, gene expression, proteomics, phosphoproteomics (using only phosphorylation sites of kinases/phosphatases with total protein abundance regressed-out, N=436) and drug response data from the COREAD cell lines in the GDSC1000. This encompassed the mutation status of 38 colorectal cancer genes, gains or losses in 48 copy number regions, methylation status of CpG islands in 32 gene promoters, gene expression and proteomics. For a fair comparison between data types (genomics, methylation, gene expression and proteomics), the same cross-validation folds were used for the different data types, and hence we only considered the 45 cell lines for which data was available in all data types. Given the limited number of available COREAD cell lines, we also limited the number of features. For the genomics and methylation we only used the Cancer Functional Events (CFEs), as defined by Iorio et al. (2016). For the proteomics, we used a subset of 2,161 most-variable proteins (the top 30% proteins, ranked by standard deviation) for which data was available in all cell lines. Similarly, for a fair comparison to the proteomics data, we considered the 2,161 most-variable transcripts. Finally, we used k-nearest neighbours (with k=10) to impute missing values in the phosphoproteomics data.

For each drug and each data type an Elastic Net model (Zou and Hastie, 2005) was fitted to predict the drug response (log IC50), using the implementation from the R package "glmnet" version 1.4 (2009). The hyper-parameter λ was optimized using 10-fold cross-validation and α was set to 0.5. Predictive performance was determined using Pearson correlation between the observed and the predicted IC50s, using the predictions from the cross-validation. When the difference in predictive performance between the optimal model and a model fitting only, an intercept was less than one standard deviation (i.e. when a model using λ1SE selects zero features), the predictive performance was set to zero. The ABCBs-corrected drug response data were the residuals of the linear regression model where the mean abundance of ABCB1/ABCB11 was used as the independent variable (x) and the log2(row mean-scaled) drug response data were used as the dependent variables (y).

Supplemental References

Aken, B.L., Achuthan, P., Akanni, W., Amode, M.R., Bernsdorff, F., Bhai, J., Billis, K., Carvalho-Silva, D., Cummins, C., Clapham, P., *et al.* (2017). Ensembl 2017. Nucleic acids research *45*, D635-D642.

Anders, S., Pyl, P.T., and Huber, W. (2015). HTSeq-a Python framework to work with high-throughput sequencing data. Bioinformatics *31*, 166-169.

Bolstad, B.M., Irizarry, R.A., Astrand, M., and Speed, T.P. (2003). A comparison of normalization methods for high density oligonucleotide array data based on variance and bias. Bioinformatics *19*, 185-193.

Cox, J., and Mann, M. (2012). 1D and 2D annotation enrichment: a statistical method integrating quantitative proteomics with complementary high-throughput data. BMC bioinformatics *13 Suppl 16*, S12.

Dobin, A., Davis, C.A., Schlesinger, F., Drenkow, J., Zaleski, C., Jha, S., Batut, P., Chaisson, M., and Gingeras, T.R. (2013). STAR: ultrafast universal RNA-seq aligner. Bioinformatics *29*, 15-21.

Gnad, F., Gunawardena, J., and Mann, M. (2011). PHOSIDA 2011: the posttranslational modification database. Nucleic acids research *39*, D253-260.

Huang, D.W., Sherman, B.T., and Lempicki, R.A. (2009). Systematic and integrative analysis of large gene lists using DAVID bioinformatics resources. Nature protocols *4*, 44-57.

Kent, W.J., Sugnet, C.W., Furey, T.S., Roskin, K.M., Pringle, T.H., Zahler, A.M., and Haussler, D. (2002). The human genome browser at UCSC. Genome research *12*, 996-1006.

Love, M.I., Huber, W., and Anders, S. (2014). Moderated estimation of fold change and dispersion for RNA-seq data with DESeq2. Genome biology *15*, 550.

Raney, B.J., Dreszer, T.R., Barber, G.P., Clawson, H., Fujita, P.A., Wang, T., Nguyen, N., Paten, B., Zweig, A.S., Karolchik, D., *et al.* (2014). Track data hubs enable visualization of user-defined genome-wide annotations on the UCSC Genome Browser. Bioinformatics *30*, 1003-1005.

Robinson, J.T., Thorvaldsdottir, H., Winckler, W., Guttman, M., Lander, E.S., Getz, G., and Mesirov, J.P. (2011). Integrative genomics viewer. Nature biotechnology *29*, 24-26.
Shannon, P., Markiel, A., Ozier, O., Baliga, N.S., Wang, J.T., Ramage, D., Amin, N., Schwikowski, B., and Ideker, T. (2003). Cytoscape: a software environment for integrated models of biomolecular interaction networks. Genome research *13*, 2498-2504.
Sigrist, C.J., Cerutti, L., de Castro, E., Langendijk-Genevaux, P.S., Bulliard, V., Bairoch, A., and Hulo, N. (2010). PROSITE, a protein domain database for functional characterization and annotation. Nucleic acids research *38*, D161-166.
Tyanova, S., Temu, T., Sinitcyn, P., Carlson, A., Hein, M.Y., Geiger, T., Mann, M., and Cox, J. (2016). The Perseus computational platform for comprehensive analysis of (prote)omics data. Nature methods *13*, 731-740.
Wright, J.C., Mudge, J., Weisser, H., Barzine, M.P., Gonzalez, J.M., Brazma, A., Choudhary, J.S., and Harrow, J. (2016). Improving GENCODE reference gene annotation using a high-stringency proteogenomics workflow. Nature communications *7*.
Zou, H., and Hastie, T. (2005). Regularization and variable selection via the elastic net. J Roy Stat Soc B *67*, 301-320.
